# Structure and Reactivity of N‐Heterocyclic Alkynyl Hypervalent Iodine Reagents

**DOI:** 10.1002/chem.202101475

**Published:** 2021-06-04

**Authors:** Eliott Le Du, Thibaut Duhail, Matthew D. Wodrich, Rosario Scopelliti, Farzaneh Fadaei‐Tirani, Elsa Anselmi, Emmanuel Magnier, Jerome Waser

**Affiliations:** ^1^ Laboratory of Catalysis and Organic Synthesis Ecole Polytechnique Fédérale de Lausanne EPFL SB ISIC LCSO, BCH 4306 1015 Lausanne Switzerland; ^2^ Institut Lavoisier de Versailles Université Paris-Saclay, UVSQ, CNRS, UMR 8180 7800 Versailles France; ^3^ Institute of Chemical Sciences and Engineering Ecole Polytechnique Fédérale de Lausanne EPFL SB ISIC GE, BCH 2111, 1015 Lausanne EPFL SB ISIC LCSO, BCH 4306 1015 Lausanne Switzerland; ^4^ Université de Tours Faculté des Sciences et Techniques 37200 Tours France

**Keywords:** alkynyl transfer, density functional calculations, heterocycles, hypervalent iodine, sulfoximine

## Abstract

Ethynylbenziodoxol(on)e (EBX) cyclic hypervalent iodine reagents have become popular reagents for the alkynylation of radicals and nucleophiles, but only offer limited possibilities for further structure and reactivity fine‐tuning. Herein, the synthesis of new N‐heterocyclic hypervalent iodine reagents with increased structural flexibility based on amide, amidine and sulfoximine scaffolds is reported. Solid‐state structures of the reagents are reported and the analysis of the I−C_alkyne_ bond lengths allowed assessing the *trans*‐effect of the different substituents. Molecular electrostatic potential (MEP) maps of the reagents, derived from DFT computations, revealed less pronounced σ‐hole regions for sulfonamide‐based compounds. Most reagents reacted well in the alkynylation of β‐ketoesters. The alkynylation of thiols afforded more variable yields, with compounds with a stronger σ‐hole reacting better. In metal‐mediated transformations, the N‐heterocyclic hypervalent iodine reagents gave inferior results when compared to the O‐based EBX reagents.

## Graphical abstract

**Four new N‐heterocyclic** alkynyl hypervalent iodine reagents have been synthesized. Their structures and reactivity are described and compared to already reported cyclic hypervalent iodine reagents, showing less extended σ holes for tosylated reagents. Good alkyne transfer properties towards strong nucleophiles were observed, but applications in radical and metal‐catalyzed processes were more limited.

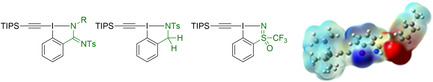

## Introduction

**Figure 1:**
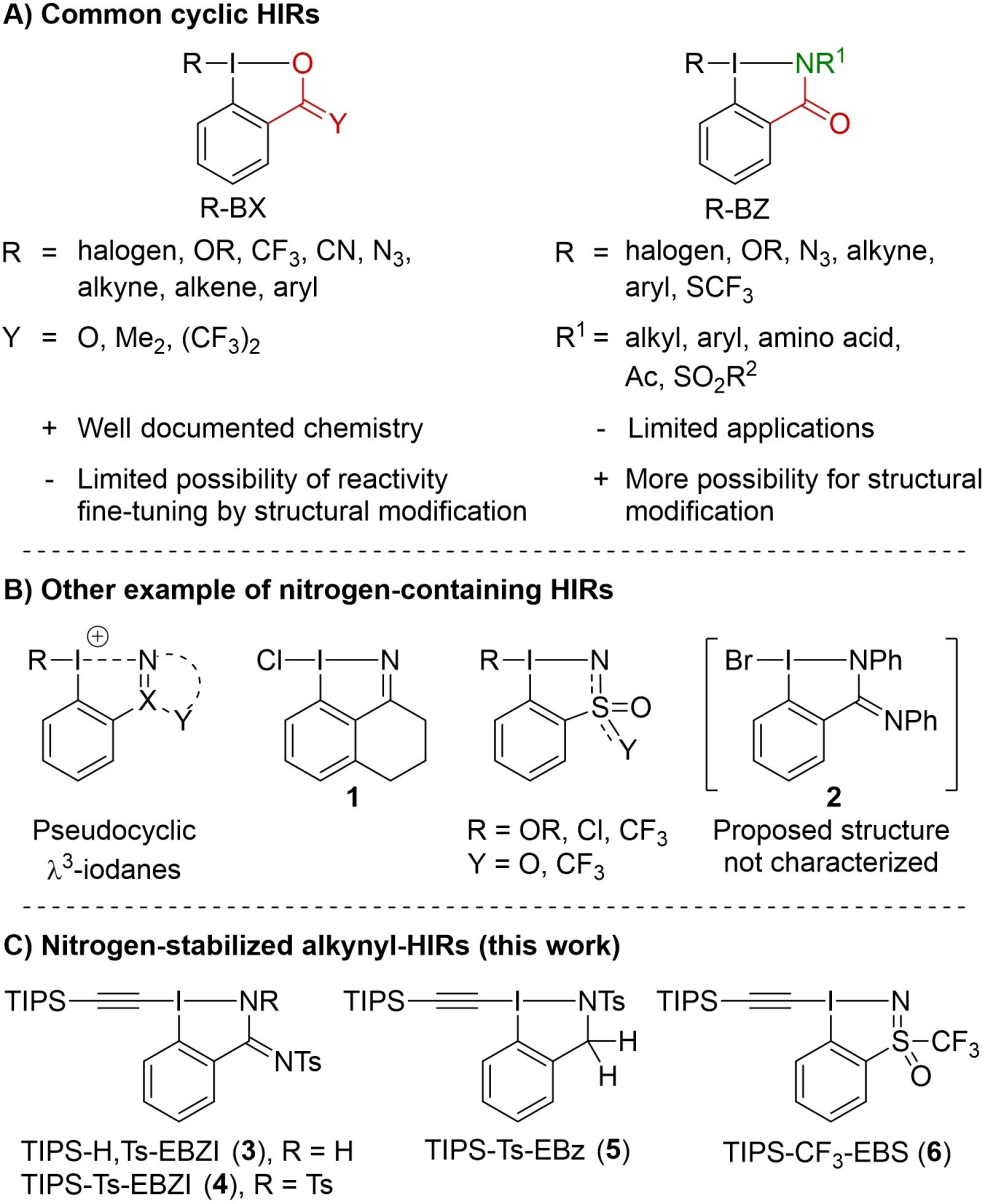
A) Established BX reagents and BZ reagents. B) Other examples of nitrogen‐stabilized HIR. C) New alkynyl reagents synthesized in this work.

In the last decades, hypervalent iodine reagents (HIR) have been established as versatile and environmentally benign oxidants and mainstream reagents for functional group transfer in organic chemistry.^[1]^ Among them, cyclic HIRs bearing a benzene ring and incorporating the iodine atom in a heterocycle exhibit higher stability. The most studied cyclic HIRs belong to the benziodoxol(on)e (BX) class of reagents characterized by an I−O bond in the iodoheterocycle (Figure 1A).^[2]^ Iodine(V) reagents, such as the Dess‐Martin Periodinane (R=(OAc)_3_, Y=O), have found widespread application as mild and stable oxidants.^[3]^ In contrast, iodine(III) reagents, such as CN−BX,^[4]^ N_3_−BX,^[5]^ CF_3_−BX (Togni's reagent),^[6]^ (hetero)Ar−BX,^[7]^ EBX (R=alkynyl),^[8]^ and VBX (R=alkene)^[9]^ have found their main applications in functional group transfer reactions. Despite their success, these reagents offer only limited options for fine‐tuning their reactivity by structural modification, as the oxygen in the heterocycle can have only one substituent. The replacement of the oxygen by a nitrogen atom in the iodoheterocycle was therefore considered by several researchers.^[10]^ The benziodazol(on)e (BZ) class of reagent was discovered in the late 60’s and most reports focused on the determination and the study of their X‐ray structure (Figure 1A).^[11]^ The first synthetic application of this type of HIR was reported only recently with a radical dehydrogenative olefination of C(sp^3^)−H bonds using an alanine derived BZ reagent.^[12]^ Later our group reported the first synthesis of EBZ (R=alkyne) reagents and studied their reactivity towards nucleophiles and in the oxyalkynylation of diazo compounds.^[13]^ Interestingly, although it had been computed that the benziodoxole‐based SCF_3_‐reagent would not be stable,^[14]^ Zhang and coworkers reported in 2020 the synthesis of SCF_3_‐BZ reagents and employed them for the trifluoromethylthiolation of various nucleophiles.^[15]^

Moreover, other types of nitrogen‐stabilized (pseudo)‐cyclic HIR have been reported in the literature (Figure 1B). The groups of Zhdankin,^[16]^ Nachtsheim^[17]^ and Tsarevsky^[18]^ studied the synthesis, the stability and the reactivity of (pseudo)cyclic N‐heterocycle hypervalent iodine compounds in oxidative transformations. Compound **1** ^[19]^ and benziodathiazole reagents (Y=O)^[20]^ were also reported in the literature but their reactivity was not investigated. More recently, the Magnier and Togni groups disclosed the synthesis of sulfoximine based reagents (Y=CF_3_) and studied their reactivity in trifluoromethylation reactions.^[21]^ Finally, Braddock and coworkers speculated on the formation of an amidine‐derived bromoiodinane reagent **2** when performing the bromolactonization of alkenes.^[22]^

Our initial goal when designing the first EBZ reagents was to develop an atom‐economical aminoalkynylation of diazo compounds that would have led to α‐amino acid derivatives.^[13]^ Unfortunately only oxyalkynylation products were obtained through the reaction of the oxygen atom on the amide. Removing the nucleophilic amide oxygen appeared therefore as a promising strategy and reagents bearing amidine, amine and sulfoximine core structures were envisioned. More generally, access to these unprecedented reagents was expected to further increase our understanding on the structure‐reactivity relationship of cyclic alkynyl hypervalent iodine reagents. Herein, we report the first synthesis of ethynylbenziodazolimine (EBZI, **3** and **4**), ethynylbenziodazole (EBz, **5**) and ethynylbenziodosulfoximine (EBS, **6**) (Figure 1C).^[23]^ Their structures as well as their reactivity in standard reactions were compared to already reported EBX and EBZ reagents. Although these reagents did not allow to develop the desired aminoalkynylation of diazo compounds, they were competent alkyne‐transfer reagents towards several nucleophiles.

## Results and Discussion

### Synthesis of the precursors

**Scheme 1:**
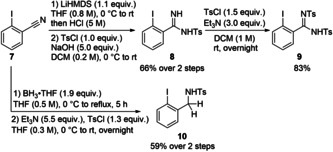
Synthesis of precursors **8**, **9** and **10** from 2‐iodobenzonitrile (**7**).

In order to access the amidine‐based and the benziodazole reagents, iodine(I) precursors **8**, **9** and **10** were obtained from commercially available 2‐iodobenzonitrile (**7**) (Scheme 1). Nucleophilic addition of LiHMDS onto the nitrile group followed by hydrolysis and tosyl protection afforded **8** in 66 % yield over two steps.^[24]^ A second tosyl protection led to compound **9** in 83 % yield. A stepwise protection procedure was favored as it would allow to finely tune the properties of the amidine‐based reagents by adding two different electron‐withdrawing groups. Reducing the electron‐density on the nitrogen atom bound to the iodine was expected to be essential to obtain stable reagents.[^11l^, ^25^] Other protecting groups such as benzoyl or Boc were introduced using similar conditions. However they were not tolerated under the reaction conditions required to form hypervalent iodine reagents and only hydrolysis of the amidine was observed in most cases. Tosyl‐protected 2‐iodobenzylamine (**10**) could be obtained in 59 % yield in two steps via borane‐reduction according to a reported procedure,^[26]^ followed by tosylation.

**Scheme 2:**
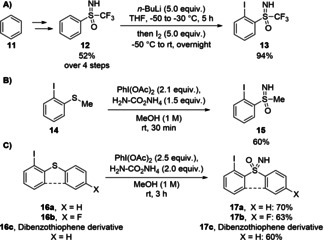
Synthesis of A) Trifluoromethyl‐substituted sulfoximine **13**, B) Methyl‐substituted sulfoximine **15**, and C) Aryl‐substituted sulfoximines **17 a**–**17 c**.

The sulfoximine precursor **12** was synthesized on multigram scale from benzene (**11**), in 52 % yield over 4 steps according to our previous methodology (Scheme 2A, see Supporting Information for details).[^21a^, ^27^] Finally, *ortho*‐iodination afforded racemic sulfoximine precursor **13** in 94 % yield. Both enantiomers of the iodosulfoximine **13** could be separated by preparative chiral HPLC and were configurationally stable (see Supporting Information for details). Four other *ortho*‐iodinated sulfoximines were synthesized from the corresponding sulfides **14** and **16 a**–**16 c** in good yields (Scheme 2B and C respectively, see Supporting Information for the preparation of sulfides).^[28]^ With both alkyl substituted sulfoximine **15** and aryl‐substituted sulfoximines **17 a**–**17 c** in hand, the influence of the group linked to the sulfur atom was then investigated.

### Synthesis and structure of the new reagents

**Scheme 3:**
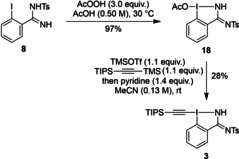
Synthesis of TIPS‐H,Ts‐EBZI (**3**).

We started our investigation on the synthesis of amidine‐based hypervalent iodine reagents by attempting the one‐pot oxidation‐alkynylation protocol reported to convert 2‐iodobenzamides into EBZs.^[13]^ Unfortunately, only degradation and hydrolysis of amidines **8** and **9** was observed under these conditions. Hence, we decided to follow a stepwise oxidation, ligand exchange strategy.[^8b^, ^11i^] Acetoxylation of monoprotected amidine **8** followed by ligand exchange after activation with trimethylsilyl triflate led to the formation of TIPS‐H,Ts‐EBZI (**3**) in 27 % yield over the two steps (Scheme 3).

**Figure 2:**
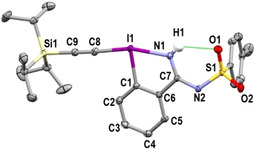
Structure of TIPS‐H,Ts‐EBZI (**3**) (see Table 1 for structural properties). H atoms (except H1) are omitted for clarity; thermal ellipsoids given at 50 % probability.

The structure of **3** was unambiguously established by X‐ray analysis (Figure 2).^[29]^ Similarly to what was observed by Zhdankin and coworkers with benziodazolone reagents, the isomer with the endocyclic free nitrogen atom was formed.^[11i]^ Moreover, the presence of a hydrogen‐bond between one of the oxygen of the sulfonyl group and the hydrogen on the endocyclic nitrogen might enhance the donating ability of the nitrogen.

**Scheme 4:**
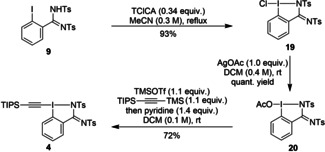
Synthesis of TIPS‐Ts‐EBZI (**4**).

Unfortunately, bis‐tosylated amidine **9** was degraded in a peracetic acid‐acetic acid solution and no hypervalent iodine reagent was detected. However, a sequence of chlorination followed by two ligand exchanges afforded TIPS‐Ts‐EBZI (**4**) in 67 % yield over three steps (Scheme 4).

**Figure 3:**
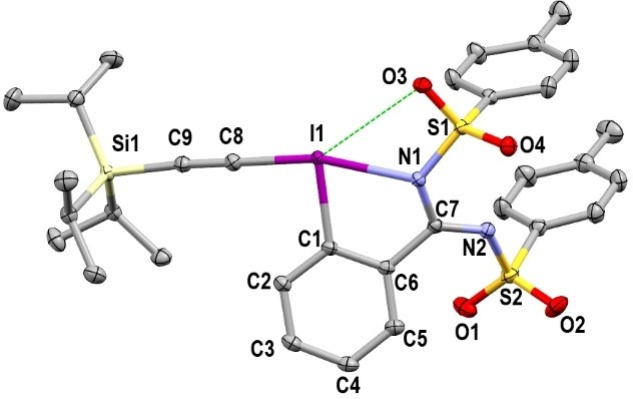
Structure of TIPS‐Ts‐EBZI (**4**) (see Table 1 for structural properties). H atoms are omitted for clarity; thermal ellipsoids given at 50 % probability.

The structure of **4** was determined by X‐ray diffraction (Figure 3).^[29]^ The presence of a potential secondary interaction between the iodine atom and an oxygen atom from a tosyl protecting group might help to stabilize the hypervalent iodine reagent.

**Scheme 5:**
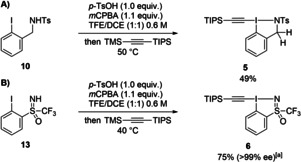
A) Synthesis of TIPS‐Ts‐EBz (**5**) B) Synthesis of TIPS‐CF_3_‐EBS (**6**).

To our delight, EthynylBenziodazole (EBz) reagent (**5**) and EthynylBenziodoSulfoximine (**6**) (EBS) could be obtained following a one‐pot two steps procedure in 49 % and 75 % yield respectively (Scheme 5).^[13]^ Moreover, starting from enantiopure sulfoximine precursor **13**, a chiral alkynyl hypervalent iodine reagent was obtained in good yield and high enantiopurity. Only few chiral alkynyl hypervalent iodine reagents have been reported so far.^[30]^ Despite our efforts, no other HIR with a sulfoximine motif was obtained starting from **15** or **17 a**–**17 c**, highlighting the essential role of the CF_3_ group of **13** for successful synthesis.

**Figure 4:**
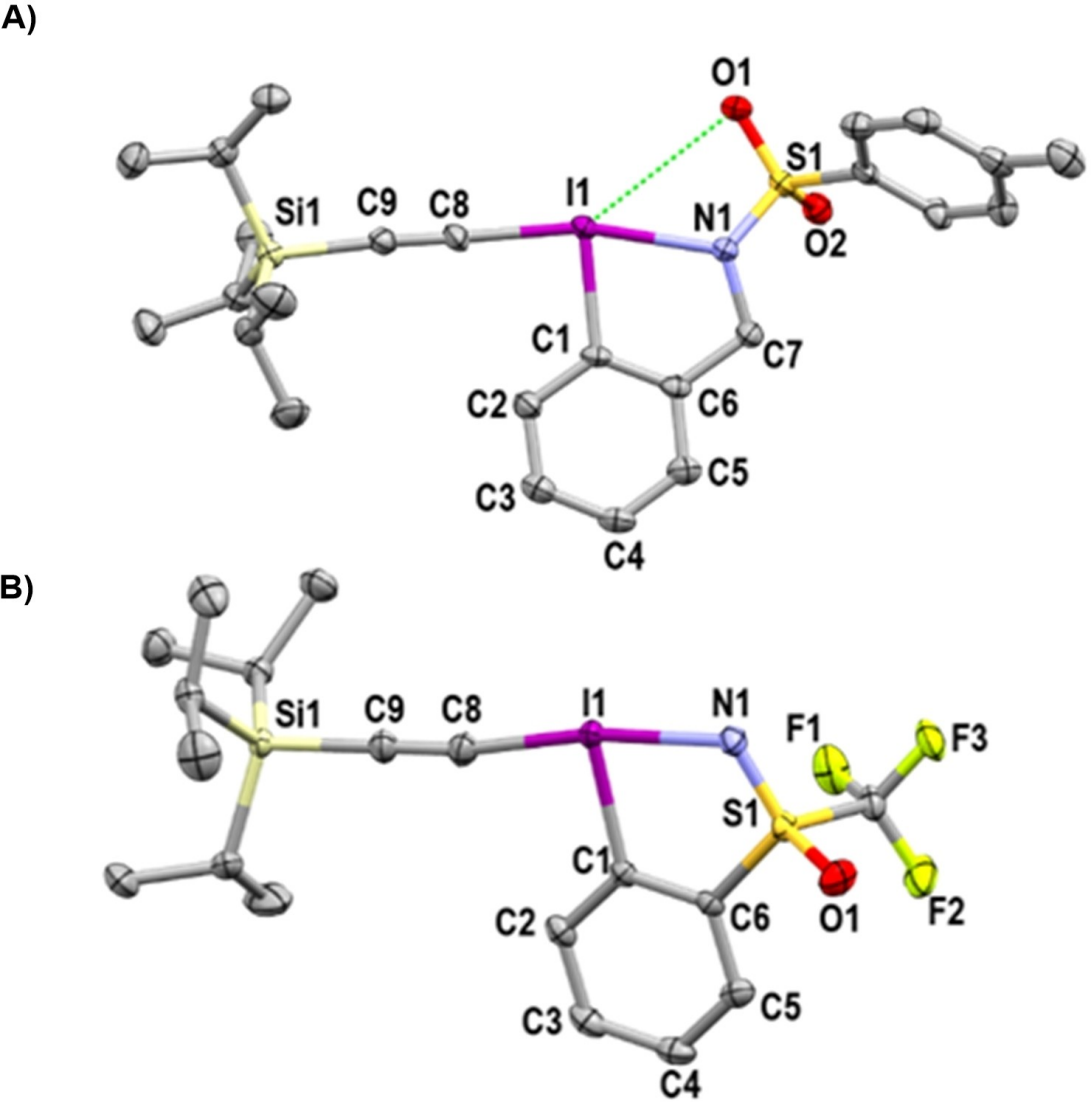
A) Structure of TIPS‐Ts‐EBz (**5**) B) Structure of TIPS‐CF_3_‐EBS (**6**) (see Table 1 for structural properties). H atoms are omitted for clarity; thermal ellipsoids given at 50 % probability.

The structure of both reagents **5** and **6** was also established by X‐ray analysis (Figure 4).^[29]^ As previously observed in reagent **4** a potential secondary interaction between an oxygen atom and the iodine atom might stabilize **5**.

The structural features of the newly obtained hypervalent iodine reagents were compared to that of the reported TIPS‐EBX (**21**) and TIPS‐Ts‐EBZ (**22**) (Table 1).^[13]^ The comparison of the I1−C8 bond lengths between the different species allows to assess the relative *trans* effects of the substituents.[^11l^, ^25^] This parameter plays an important role in the stability of hypervalent iodine species and is usually related to the donating ability of the ligands along the 3c–4e^−^ bond. The longer the I1−C8 bond, the higher the *trans* influence of the corresponding ligand. From the measured values, we can conclude that the monoprotected amidine **3** and the sulfonamide **5** are the ligands having the higher *trans* influence of the studied substituents (entries 1 and 2). The sulfoximine ligand in **6** has a slightly lower influence (entry 3, I1−C8=2.089(3) Å vs. 2.102(3) and 2.100(3) for **3** and **5**). The benziodazolone and benziodoxolone moieties have similar *trans* effect (I1−C8=2.060(9) Å and 2.054(2) for **22** and **21** respectively, entries 4 and 5). Finally the diprotected amidine has the lower *trans* influence (I1−C8=2.046(2) Å for **4**, entry 6). The iodine‐heteroatom bond length is similar for EBz (**5**), EBS (**6**) and EBX (**21**) (2.333(3), 2.337(2), 2.338(1) Å respectively, entries 2, 3 and 5). On the other hand, for the TIPS‐Ts‐EBZI (**4**) reagent, this bond is significantly longer (2.443(1) Å, entry 6), probably due to higher steric hindrance. As observed in TIPS‐EBX (**21**) and TIPS‐Ts‐EBZ (**22**) (entries 5 and 4), the hypervalent bond in the new reagents was close to linearity with X1−I1−C8 angle ranging from 164.74(9)° for **3** (entry 1) to 171.23(1)° for **6** (entry 3). The C1−I1−C8 angles between the alkyne and the benzene range from 90.0(1)° for **3** (entry 1) to 93.03(6)° for **4** (entry 6). These angles are fully coherent with the T‐shape structure expected for hypervalent iodine reagents. The torsion angle (C8−I1−C1−C2) showed that in most cases the alkynyl bond is in the plane of the aromatic ring from −8.33(2)° for **21** (entry 5) to 3.44(2)° in **3** (entry 1). In the case of TIPS‐Ts‐EBZI (**4**) the torsion angle is relatively high −16.56(1)° (entry 6). Finally, in reagents **5**, **22** and **4** the short distance between one oxygen atom of the tosyl protecting group and the iodine center (3.246(3) Å for **5**, 3.285(5) Å for **22**, 3.319(1) Å for **4**, entries 2, 4 and 6) indicated a possible interaction.

**Table 1:** Comparison of bond lengths and bond angles in alkynyl hypervalent iodine reagents obtained from single crystal X‐ray diffraction. X=O or N. The torsion angle corresponds to C8−I1−C1−C2.

Entry	Reagent	I1−C8 [Å]	I1−X1 [Å]	X1−I1−C8 [°]	C1−I1−C8 [°]	Torsion [°]	I−O_sulfonyl_ [Å]
1	TIPS‐H,Ts‐EBZI (**3**)	2.102(3)	2.317(2)	164.74(9)	90.0(1)	3.44(2)	–
2	TIPS‐Ts‐EBz (**5**)	2.100(3)	2.333(3)	165.99(1)	90.77(1)	−0.49(3)	3.246(3)
3	TIPS‐CF_3_‐EBS (**6**)	2.089(3)	2.337(2)	171.23(1)	90.31(1)	2.85(2)	−
4^[a]^	TIPS‐Ts‐EBZ (**22**)	2.060(9)	2.387(6)	165.79(3)	92.08(3)	−6.63(7)	3.285(5)
5^[a]^	TIPS‐EBX (**21**)	2.054(2)	2.338(1)	166.11(6)	91.37(7)	−8.33(2)	–
6	TIPS‐Ts‐EBZI (**4**)	2.046(2)	2.443(1)	165.51(5)	93.03(6)	−16.56(1)	3.319(1)

[a] Data taken from the literature.^[13]^

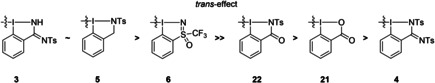

**Figure 5:**
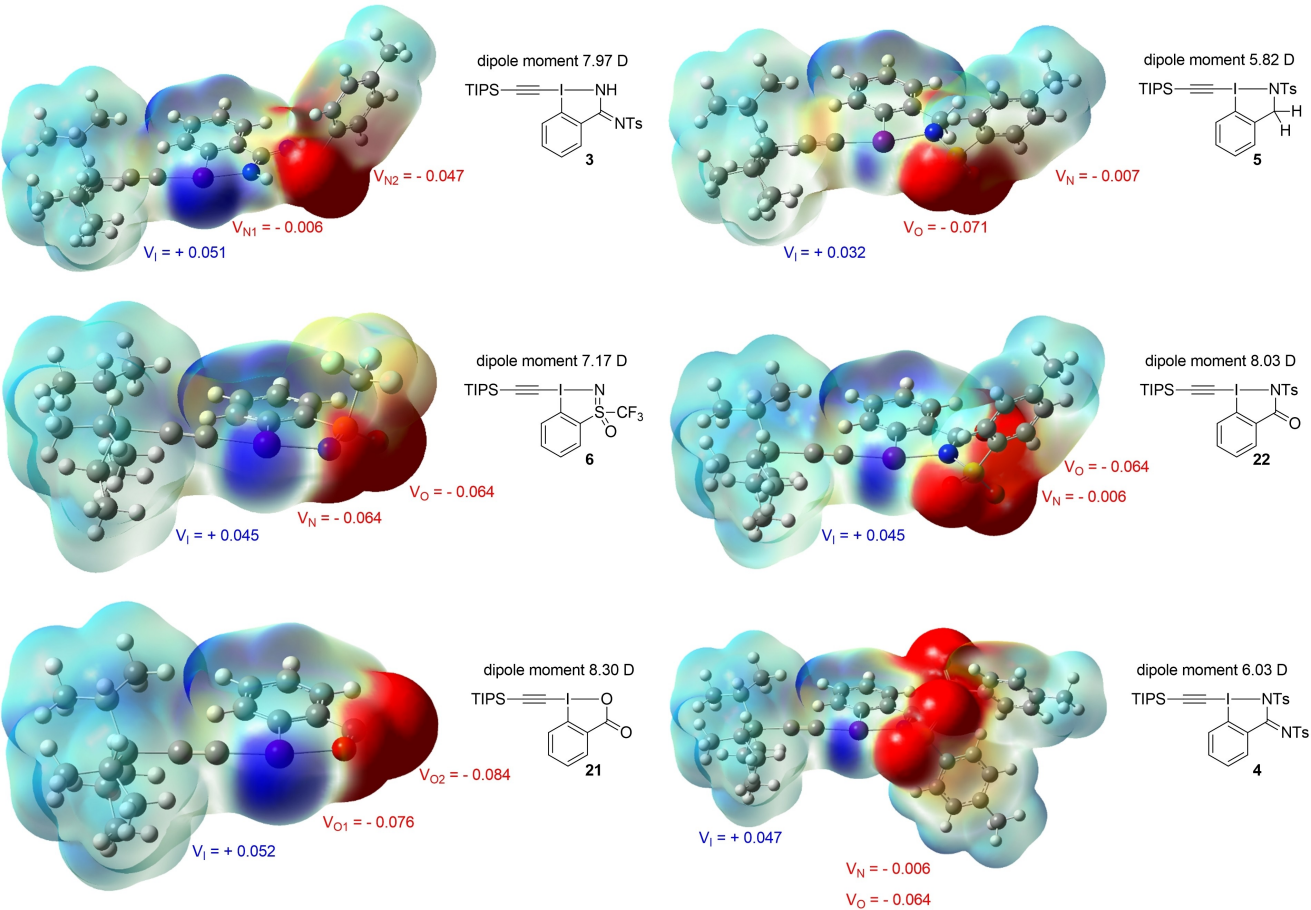
Molecular electrostatic potential (MEP) maps computed at the M06/def2‐SVP level. MEPs were mapped onto the 0.001 au isodensity surface. V_X_ represents the potential maximum around the atom X and is given in au (see Supporting Information for details).

In order to get a deeper understanding on the electronic effects of the different substituents next to the iodine atom, molecular electrostatic potential (MEP) maps were generated (Figure 5, see Supporting Information for details). A strong polarization towards the ligand is observed in almost all the reagents. The calculated molecular dipole moment is higher for **21** (8.30 D) followed by **22** (8.03 D). The new reagents have lower calculated dipole moment, 7.97 D for monoprotected amidine **3**, 7.17 D for the sulfoximine **6**, 6.03 D for the diprotected amidine **4** and finally 5.82 D for the benziodazole **5**. The MEPs allowed also to visualize the σ‐hole regions of the reagents. These regions of positive charge at the iodine atom play an important role on the strength and the directionality of reagent‐solvent or ‐substrate interactions.^[31]^ The most extended positive regions are observed for TIPS‐EBX (**21**), TIPS‐EBS (**6**) and TIPS‐H,Ts‐EBZI (**3**), while the reagents having a tosyl group on the heteroatom bound to the iodine atom (TIPS‐Ts‐EBz (**5**), TIPS‐Ts‐EBZ (**22**) and TIPS‐Ts‐EBZI (**4**)) have a less extended σ‐hole region. As observed in the X‐ray structures of these reagents, the secondary interaction between one of the oxygen of the tosyl group and the iodine atom might weaken the σ‐hole. From the MEPs plots, local potential maximum (V_x,max_) around the atoms can also be extracted. In all the reagents the potential maximum around the C(sp) bound to the iodine atom (V_c,max_) and around the iodine (V_I,max_) are similar (between −0.002 and +0.005 and +0.032 and +0.052 au respectively, see Supporting Information). The iodoheterocycle structure seems to have an impact on the spatial extension of the σ‐hole region, but not on the potential maximum on the iodine. While the oxygen atom bound to iodine from TIPS‐EBX (**21**) and the nitrogen atom bound to iodine from **6** have a significant negative electron density, the nitrogen bound to iodine in the other reagents is almost neutral. The negative charge seems in fact transferred to the oxygen of the sulfonamide for TIPS‐Ts‐EBz (**5**) and TIPS‐Ts‐EBZI (**4**) or on the other heteroatom present in the iodoheterocycle for TIPS‐H,Ts‐EBZI (**3**) and TIPS‐Ts‐EBZ (**22**). The higher electron‐density on the oxygen in TIPS‐Ts‐EBZ (**22**) reagent might explain why only oxyalkynylation was observed with diazo compounds and not the targeted aminoalkynylation.^[13]^ Overall, these observations might indicate that the major resonance structures for these reagents are not the one drawn, but instead ionic structures with a positively charged iodine atom and a negative charge on the oxygen atoms.

### Comparison of the reactivity of the new reagents

**Scheme 6:**
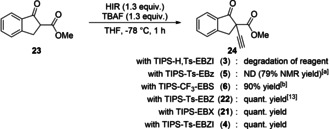
Alkynylation of keto ester **23**. ^[a]^ The desired product could not be separated from side‐products, CH_2_Br_2_ was used as internal standard. ^[b]^ No stereoinduction was observed when enantiopure reagent **6** was employed.

With the new reagents in hand, we investigated their reactivity in electrophilic alkynylations and compared them to the already existing reagents TIPS‐EBX (**21**) and TIPS‐Ts‐EBZ (**22**). First, we focused our attention on the alkynylation of β‐ketoester **23** (Scheme 6).^[32]^ In the case of the monoprotected amidine‐based reagent **3**, only degradation of the reagent was observed. In the case of reagent **5**, alkyne‐transfer was observed, but the side product *ortho*‐iodobenzylamine could not be separated from the desired product **24**. The sulfoximine‐based reagent **6** afforded **24** in 90 % yield. Unfortunately, when using enantiopure sulfoximine reagent **6**, only racemic alkyne **24** was obtained. Probably, the chiral center in the reagent is too far from the reactive site to induce any selectivity. Finally, reagents **22**, **21** and **4** afforded **24** in quantitative yield.

**Scheme 7:**
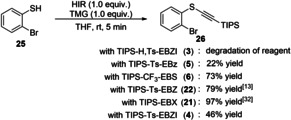
Alkynylation of thiol **25**.

Next, we studied the thioalkynylation of *ortho*‐bromothiophenol (**25**) following our reported procedure (Scheme 7).^[32]^ When using the monoprotected amidine based reagent **3**, the desired product was not detected and only degradation of the reagent was observed. Alkynylated product **26** could be obtained in low yield from TIPS‐Ts‐EBz (**5**). Reagents with more pronounced σ‐hole regions TIPS‐CF_3_‐EBS (**6**), TIPS‐Ts‐EBZ (**22**)^[13]^ and TIPS‐EBX (**21**)^[33]^ afforded **26** in good to excellent yields. Finally, TIPS‐Ts‐EBZI (**4**) led to the formation of **26** in 46 % yield. Computations suggested that the accessibility of the σ‐hole was key for success in this transformation.^[34]^ The lower yields often observed with tosylated reagents (with the exception of **22**) with a smaller σ‐hole region is in accordance with this hypothesis.

**Scheme 8:**
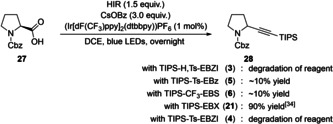
Photoredox‐catalyzed decarboxylative‐alkynylation of Cbz‐proline **27**.

The photoredox‐catalyzed decarboxylative alkynylation of carboxylic acids was then investigated (Scheme 8).^[35]^ The procedure developed for EBX reagents afforded alkynylated proline **28** in 90 % yield with TIPS‐EBX (**21**). Unfortunately, these conditions were not suitable for the amidine based reagents as only degradation was observed with **3** and **4**. When benzylamine or sulfoximine based reagents **5** and **6** were submitted to the reaction conditions, around 10 % of the desired alkynylated proline **28** was obtained.

**Scheme 9:**
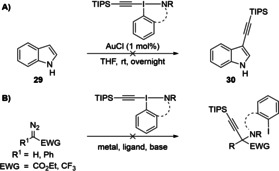
Unsuccessful A) Alkynylation of indole (**29**) and B) Aminoalkynylation of diazo compounds.

Finally, the gold‐catalyzed alkynylation of indole (**29**)^[36]^ was studied using the newly developed reagents, but no C3‐alkynylated indole **30** was isolated (Scheme 9A). Likewise, all attempts to employ these reagents in the atom‐economical aminoalkynylation of diazo compounds under the conditions developed for oxyalkynylation[^13^, ^37^] did not afford promising results (Scheme 9B). The lack of reactivity could be linked to the lack of nitrogen nucleophilicity observed on the MEP maps.

## Conclusions

In summary, we have synthesized four new N‐heterocyclic alkynyl hypervalent iodine reagents and elucidated their solid‐sate structures via X‐ray crystallography. We demonstrated the possibility to modulate the iodine‐alkyne bond length by modifying the iodoheterocycle and fine‐tuning the *trans* effect. The modification of the iodoheterocycle had also a clear impact on the distribution of the electron density on the reagents. Computed MEP maps showed a transfer of electron‐density away from the nitrogen bound to the iodine, and a diminished σ‐hole region in the case of tosylated reagents. Finally, the reactivity of the new hypervalent compounds was compared to the already established EBX and EBZ reagents. The alkynylation of β‐ketoester was successful with all new reagents, except for the unstable reagent **3** bearing an unprotected amidine group. On the other hand, only reagents with more pronounced σ‐hole regions reacted well in the thioalkynylation reaction. Transfer to radical intermediates was possible in low yield for the benzylamine and sulfoximine based reagents in the photoredox‐catalyzed decarboxylative alkynylation of proline.

## Conflict of interest

The authors declare no conflict of interest.

## Supporting information

As a service to our authors and readers, this journal provides supporting information supplied by the authors. Such materials are peer reviewed and may be re‐organized for online delivery, but are not copy‐edited or typeset. Technical support issues arising from supporting information (other than missing files) should be addressed to the authors.

Supporting Information
