# Supplementary material for: Structure and Reactivity of N‐Heterocyclic Alkynyl Hypervalent Iodine Reagents
Source: Chemistry. 2021 Jun 4;27(42):10979–86. doi: 10.1002/chem.202101475 (PMC8361724; doi:10.1002/chem.202101475)

Chemistry–A European Journal

Supporting Information

Structure and Reactivity of N-Heterocyclic Alkynyl Hypervalent Iodine Reagents

Eliott Le Du, Thibaut Duhail, Matthew D. Wodrich, Rosario Scopelliti, Farzaneh Fadaei-Tirani, Elsa Anselmi, Emmanuel Magnier,* and Jerome Waser*

Table of Contents

1. General methods 2
2. Preparation of precursors 4
3. Preparation of hypervalent iodine reagents 11
4. Reactivity investigation 15
5. X-ray crystallographic data 18
5.1. Single Crystal X-Ray Diffraction for the compound 3 18
5.2. Single Crystal X-Ray Diffraction for the compound 4 25
5.3. Single Crystal X-Ray Diffraction for the compound 5 34
5.4. Single Crystal X-Ray Diffraction for the compound 6 39
6. DFT calculations and coordinates 47
7. NMR spectra 59

1. General methods

All reactions were carried out in oven dried glassware under an atmosphere of nitrogen, unless for the oxidative decarboxylation and if stated otherwise. For flash chromatography, distilled technical grade solvents were used. THF, CH_3CN, toluene and CH_2Cl_2 were dried by passage over activated alumina under nitrogen atmosphere (H_2O content < 10 ppm, Karl-Fischer titration). All chemicals were purchased from Acros, Aldrich, Fluka, VWR, TCI, Merck or Bachem and used as such unless stated otherwise. All dipeptides starting materials were commercially available and used as received. Chromatographic purification was performed as flash chromatography using Macherey-Nagel silica 40-63, 60 Å, using the solvents indicated as eluent with 0.1-0.5 bar pressure. TLC was performed on Merck silica gel 60 F254 TLC aluminum or glass plates and visualized with UV light and $KMnO_4$ stain. ^{1}H-NMR spectra were recorded on a Brucker DPX-400 400 MHz spectrometer in chloroform-d, DMSO-d^6 or acetonitrile-d^3, all signals are reported in ppm with the internal chloroform signal at 7.26 ppm, the internal DMSO signal at 2.50 ppm or the internal acetonitrile signal at 1.94 ppm as standard. The data is being reported as (s = singlet, d = doublet, t= triplet, q = quadruplet, qi = quintet, m = multiplet or unresolved, br = broad signal, app = apparent, coupling constant(s) in Hz, integration, interpretation).^{13}C-NMR spectra were recorded with ^{1}H-decoupling on a Brucker DPX-400 100 MHz spectrometer in chloroform-d, DMSO-d^6 or acetonitrile-d^3, all signals are reported in ppm with the internal chloroform signal at 77.0 ppm, the internal DMSO signal at 39.5 ppm or the internal acetonitrile signals at 1.32 and 118.26 ppm as standard. Infrared spectra were recorded on a JASCO FT-IR B4100 spectrophotometer with an ATR PRO410-S and a ZnSe prisma and are reported as cm-1 (w = weak, m = medium, s = strong, br = broad).

High resolution mass spectrometric measurements were performed by the mass spectrometry service of ISIC at the EPFL on a MICROMASS (ESI) Q-TOF Ultima API. MS-MS analyses were performed on a LTQ Orbitrap FTMS instrument (LTQ Orbitrap Elite FTMS, Thermo Scientific, Bremen, Germany) operated in the positive mode coupled with a robotic chip-based nano-ESI source (TriVersa Nanomate, Advion Biosciences, Ithaca, NY, U.S.A.). A standard data acquisition and instrument control system was utilized (Thermo Scientific) whereas the ion source was controlled by Chipsoft 8.3.1 software (Advion BioScience). Samples were loaded onto a 96-well plate (Eppendorf, Hamburg, Germany) within an injection volume of 5µl. The experimental conditions for the ionization voltage was +1.4kV and the gas pressure was set at 0.30 psi. The temperature of ion transfer capillary was 275 °C, tube voltages. FTMS spectra were obtained in the 80-1000 *m/z* range in the reduce profile mode with a resolution set to 120,000. In all spectra one microscan was acquired with a maximum injection time value of 1000ms. Typical CID experiments were carried out using Normalized collision energy values of 26-28 and 5 Da of isolation width.

Versailles instrumentation

NMR spectra were collected on a Bruker AC-300 spectrometer operating at the denoted spectrometer frequency given in MHz for the specified nucleus. Reported coupling constants and chemicals shifts were based on a first order analysis. $CFCl_3$ (0.00 ppm) was used as internal reference for ^{19}F NMR spectra. High resolution mass spectrometry (HRMS) was recorded on a Mass Spectrometer XEVO-QTOF in the Institute Lavoisier of Versailles – University of Versailles Saint Quentin.

Photoredox catalyzed reactions were performed in test tubes (5 and 10 mL), which were hold using a rack for test tubes placed at the center of a crystallization flask. On this flask were attached the blue LEDs (RUBAN LED 5MÈTRES - 60LED/M - 3528 BLEU - IP65 with Transformateur pour Ruban LED 24W/2A/12V, bought directly on RubanLED.com). The distance between the LEDs

and the test tubes was approximatively 2 cm for the test tubes and 5 cm for the Schlenk flasks. Long irradiation resulted in temperature increasing up to 37°C during overnight reactions.

HPLC analysis on chiral stationary phase was performed on a Agilent Acquity instrument using a Daicel CHIRALPAK IA, IB-N5 and IC chiral columns. The exact conditions for the analyses are specified within the characterization section. HPLC traces were compared to racemic samples prepared by running the reactions using racemic substrates. Absolute values of enantiomeric excesses are reported.

2. Preparation of precursors

2-Iodobenzamidine hydrochloride (31)

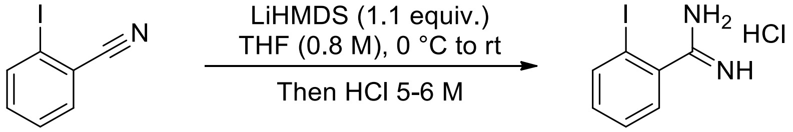

7 **31**

Following a reported procedure,[1] an oven-dried 250 mL flask was charged with LiHMDS (22 mL, 22 mmol, 1.1 equiv.) and cooled to 0 °C and a solution of 2-iodobenzonitrile (**7**) (4.6 g, 20 mmol, 1.0 equiv.) in 2.5 mL of dry THF was added dropwise and the reaction mixture was stirred at this temperature for 15 min. The reaction mixture was then stirred at room temperature for 4h. After cooling the reaction mixture to 0 °C, HCl (5 M in isopropanol, 12 mL, 60 mmol, 3.0 equiv.) was added dropwise. The reaction mixture was stirred at 0 °C and let warm up to rt. The precipitated product was filtered, washed with Et_2O and dry on the filter for 1 h to afford the title compound (**31**) as a white solid (5.1 g, 18 mmol, 90% yield).

^{1}H NMR (400 MHz, DMSO-d6) δ 8.90 (br s, 4H, N*H*$_2$), 8.01 (dd, *J* = 8.0, 1.0 Hz, 1H, Ar*H*), 7.63-7.49 (m, 2H, Ar*H*), 7.35 (ddd, *J* = 7.9, 7.2, 2.0 Hz,1H, Ar*H*). ^{13}C NMR (101 MHz, DMSO-*d*$_6$) δ 167.7, 139.4, 136.0, 132.9, 129.0, 128.4, 94.9.

The characterization data corresponded to the reported values.[2]

2-Iodo-N-tosylbenzimidamide (8)

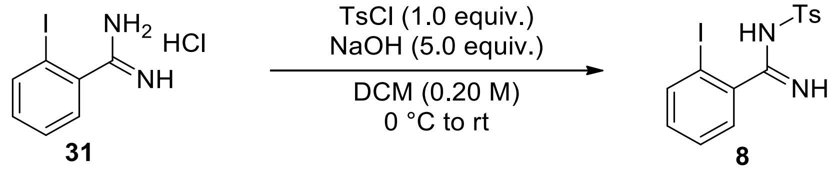

31 **8**

Following a slightly modified reported procedure,[3] a round bottom flask was loaded with 2-iodobenzimidamide.HCl (2.1 g, 7.4 mmol, 1.0 equiv.), *p*-toluenesulfonyl chloride (1.4 g, 7.4 mmol, 1.0 equiv.) and DCM (37 mL). Subsequently, the solution was cooled down to 0 °C and a 10 M aqueous solutiom of NaOH (3.7 ml, 37 mmol, 5.0 equiv.) was added slowly. The reaction mixture was stirred for 5 h at room temperature. The mixture was washed with HCl 1M (3X20 mL), the organic layer was dried over MgSO4 and concentrated under vacuum The crude mixture was purified by flash column chromatography (Pentane/EtOAC 1:2 to 1:1) to afford the title compound (**8**) as a white solid (2.2 g, 5.5 mmol, 74% yield)

R_f = 0.3 (Pentane/EtOAc 2:1). Mp: 136-138 °C. ^{1}H NMR (400 MHz, Chloroform-*d*) δ 8.37 (br s, 1H, *H*NTs), 7.88 (d, *J* = 8.2 Hz, 2H, Ar*H*), 7.81 (d, *J* = 7.8 Hz, 1H, Ar*H*), 7.37 (qd, *J* = 7.7, 1.5 Hz, 2H, Ar*H*), 7.29 (d, *J* = 8.1 Hz, 2H, Ar*H*), 7.13-7.06 (m, 1H, Ar*H*), 6.05 (br s, 1H, C=N*H*), 2.41 (s, 3H, C*H*$_3$). ^{13}C NMR (101 MHz, Chloroform-*d*) δ 165.1, 143.4, 140.2, 139.9, 138.4, 131.9, 129.5, 128.9, 128.5, 127.1, 92.9, 21.7. IR(ν_{max}, cm^{-1}) 3369 (w), 3284 (w), 3121 (w), 3057 (w), 1629 (m), 1535 (m), 1416 (w), 1297 (m), 1142 (s), 1082 (s), 1018 (w), 835 (m), 806 (m), 788 (m), 751 (s), 716 (s), 685 (s), 656 (s). HRMS (ESI/QTOF) m/z: [M + Na]$^+$ Calcd for $C_{14}H_{13}IN_2NaO_2S^+$ 422.9635; Found 422.9641.

2-Iodo-N,N'-ditosylbenzimidamide (9)

---

[1] S. Dalai, V. N. Belov, S. Nizamov, K. Rauch, D. Finsinger, A. de Meijere, *European Journal of Organic Chemistry* **2006**, *2006*, 2753–2765.

[2] T. Yao, *Tetrahedron Letters* **2015**, *56*, 4623–4626.

[3] M. Baeten, B. U. W. Maes, *Adv. Synth. Catal.* **2016**, *358*, 826–833.

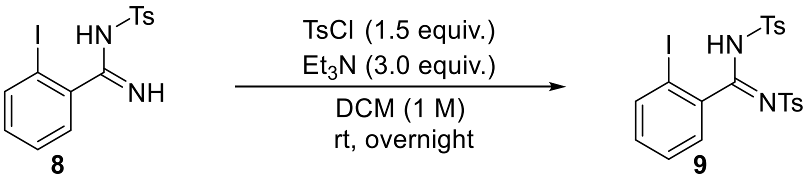

Following a slightly modified procedure,[4] an oven-dried 10 mL microwave vial was charged with 2-iodo-N-tosylbenzimidamide (1.5 g, 3.8 mmol, 1.0 equiv) and triethylamine (0.80 mL, 5.6 mmol, 1.5 equiv) and 1.9 mL of dry DCM. After 10mins a solution of *p*-toluenesulfonyl chloride (1.1 g, 5.6 mmol, 1.50 equiv) and triethylamine (0.80 mL, 5.6 mmol, 1.5 equiv) in 1.9 mL of dry DCM was added dropwise to the reaction mixture. The reaction mixture was stirred at rt overnight. The reaction mixture was then diluted with DCM (10 mL), and the mixture was washed 1M HCL (3X 10 mL). The organic phase was combined with a dichloromethane extract of the aqueous phase, dried (MgSO4), and concentrated under vacuum. The crude mixture was purified by flash column chromatography using DCM/MeOH 2% as mobile phase to afford the title compound as a yellowish solid (**9**) (1.7 g, 3.1 mmol, 83% yield).

R_f = 0.27 (DCM/MeOH 4%). Mp: 185-187 °C. ^{1}H NMR (400 MHz, Acetonitrile-*d*$_3$) δ 9.36 (s, 1H, N*H*), 7.84 (dd, *J* = 8.0, 0.7 Hz, 1H, Ar*H*), 7.62 (br s, 4H, Ar*H*), 7.47 (td, *J* = 7.6, 1.1 Hz, 1H, Ar*H*), 7.36-7.12 (m, 6H), 2.43 (s, 6H, C*H*$_3$). ^{13}C NMR (101 MHz, Acetonitrile-*d*$_3$) δ 161.8, 139.8, 138.6, 132.7, 130.3, 129.8, 128.8, 128.0, 94.1, 21.7.[5] IR (v_{max}, cm^{-1}) 3667 (w), 2978 (m), 2902 (m), 1603 (m), 1454 (m), 1352 (m), 1311 (m), 1168 (s), 1146 (s), 1083 (s), 932 (m), 818 (m), 768 (m), 720 (s), 682 (s). HRMS (APPI/LTQ-Orbitrap) m/z: [M + H]$^+$ Calcd for $C_{21}H_{20}IN_2O_4S_2^+$ 554.9904; Found 554.9902.

2-Iodobenzylamine (32)

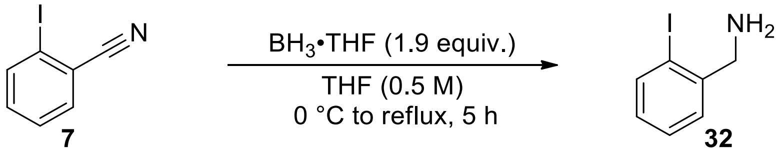

Following a slightly modified procedure,[6] in an oven dried round-bottom flask 2-iodobenzonitrile (5.0 g, 22 mmol, 1.0 equiv.) and dry THF (44 mL) were mixed together, then borane-THF complex (41 ml, 41 mmol, 1.9 equiv.) was added dropwise to the solution at 0 °C. The mixture was refluxed under stirring for 5 h, then it was hydrolyzed at 0 °C with HCl 6 N until pH ~ 1; after, it was made basic with KOH until pH ~ 13 and extracted with DCM. The combined organic phases were washed with brine, dried over $MgSO_4$ and the solvent was removed under vacuum. The crude mixture was purified by flash column chromatography (DCM to DCM/MeOH 20:1) to afford the title compound as green oil (**32**) (3.1 g, 13 mmol, 61% yield)

Rf = 0.36 (DCM/MeOH 9:1). ^{1}H NMR (400 MHz, Chloroform-*d*) δ 7.82 (d, *J* = 7.8 Hz, 1H, Ar*H*), 7.42-7.28 (m, 2H, Ar*H*), 6.95 (td, *J* = 7.7, 1.9 Hz, 1H, Ar*H*), 3.87 (s, 2H, ArC*H*$_2$NH$_2$), 1.64 (s, 2H, N*H*$_2$). ^{13}C NMR (101 MHz, Chloroform-*d*) δ 145.2, 139.6, 128.8, 128.7, 128.6, 99.1, 51.5.

The characterization data corresponded to the reported values.[7]

N-(2-iodobenzyl)-4-methylbenzenesulfonamide (10)

---

[4] A. Guzmán, M. Romero, F. X. Talamás, R. Villena, R. Greenhouse, J. M. Muchowski, *J. Org. Chem.* **1996**, *61*, 2470–2483.

[5] 2 carbons were not resolved by ^{13}C in acetonitrile-*d*$_3$

[6] L. A. Aronica, G. Albano, L. Giannotti, E. Meucci, *Eur. J. Org. Chem.* **2017**, *2017*, 955–963.

[7] T. Fukuyama, T. Bando, I. Ryu, *Synthesis* **2018**, *50*, 3015–3021.

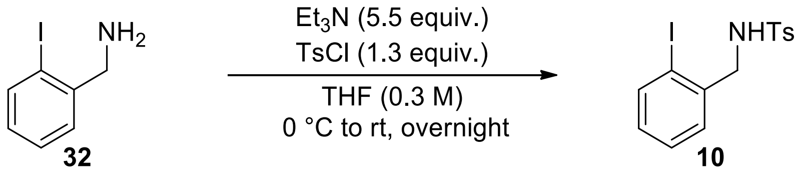

Following a slightly modified procedure,[6] in an oven dried round-bottom flask 2-iodobenzylamine (1.0 g, 4.3 mmol, 1.0 equiv.) triethylamine (3.3 ml, 24 mmol, 5.5 equiv.) and dry THF (14 mL) were mixed together, then *p*-toluenesulfonyl chloride (1.1 g, 5.6 mmol, 1.3 equiv.) was added to the solution at 0 °C. The solution was left under stirring overnight at room temperature, then it was extracted with EtOAc. The combined organic phases were washed with H_2O and brine, dried over $MgSO_4$ and the solvent was removed under vacuum. The crude mixture was purified by flash column chromatography (Pentane/EtOAc 10:1) to afford the title compound as a white solid (**10**) (1.6 g, 4.2 mmol, 97% yield).

^1H NMR (400 MHz, Chloroform-*d*) δ 7.75-7.70 (m, 3H, Ar*H*), 7.32-7.22 (m, 4H, Ar*H*), 6.94 (td, *J* = 7.6, 1.9 Hz, 1H, Ar*H*), 4.90 (t, *J* = 6.4 Hz, 1H, N*H*), 4.18 (d, *J* = 6.5 Hz, 2H, ArCH_2NHTs), 2.41 (s, 3H, CH_3). ^{13}C NMR (101 MHz, Chloroform-*d*) δ 143.6, 139.6, 138.7, 137.0, 130.2, 129.8(3C), 128.7, 127.3, 98.9, 51.9, 21.7.

The characterization data corresponded to the reported values.[6]

((trifluoromethyl)sulfinyl)benzene (33)

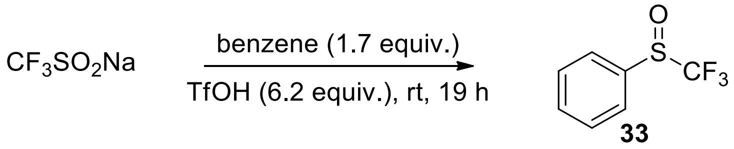

A dry 1 L, three-necked, round-bottomed flask equipped with a thermometer and a mechanical stirrer was charged with sodium trifluoromethanesulfinate (90 g, 0.58 mol, 1.0 equiv.) and dried under vacuum for 24 h prior to use. The flask is placed in a cold-water bath and trifluoromethanesulfonic acid (0.32 L, 3.6 mol, 6.2 equiv.) is added, under argon, in three portions with vigorous stirring (around 100 mL each), in order to keep the temperature under 50 °C. After the addition, the reaction is stirred for 20−30 min until the temperature decreases to room temperature. Then, benzene (90 mL, 1.0 mol, 1.7 equiv.) is added in one portion and the solution is stirred at room temperature for 19 h under an inert atmosphere. The reaction is quenched by pouring the reaction medium on ice (900 g), extracted with dichloromethane (3 × 100 mL), and washed with a saturated solution of $NaHCO_3$ (3 × 60 mL). The organic phase is dried over $MgSO_4$, filtered, and concentrated under reduced pressure. The product is purified by distillation under reduced pressure (78−80 °C at 15 mmHg) to afford the title compound as a colorless oil (**33**) (78 g, 0.40 mol, 69% yield).

^1H NMR (300 MHz, $CDCl_3$) δ 7.76 (d, *J* = 7.4 Hz, 2H, Ar*H*), 7.70−7.49 (m, 3H, Ar*H*). ^{13}C NMR (75 MHz, $CDCl_3$) δ 135.6 (q, *J* = 1.7 Hz), 133.6, 129.6, 125.9, 124.7 (q, *J* = 335 Hz, CF_3). ^{19}F NMR (282 MHz, $CDCl_3$) δ −75.0 (s, 3F).

The characterization data corresponded to the reported values.[8]

(S-(trifluoromethyl)sulfonimidoyl)benzene (12)

[8] A.-L. Barthelemy, V. Certal, G. Dagousset, E. Anselmi, L. Bertin, L. Fabien, B. Salgues, P. Courtes, C. Poma, Y. El-Ahmad, E. Magnier, *Org. Process Res. Dev.* **2020**, *24*, 704–712.

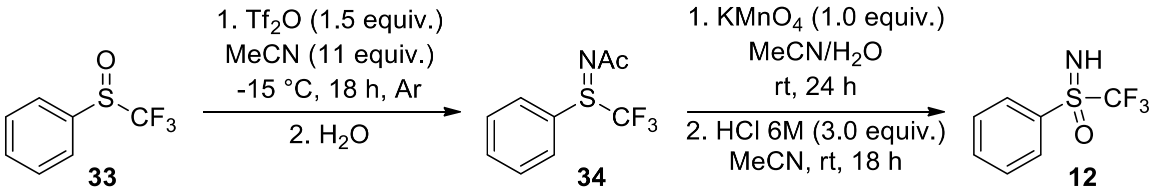

In a dry 500 mL two-necked round-bottomed flask equipped with a dropping-funnel and a thermometer, a solution of phenyl trifluoromethyl sulfoxide **33** (40.0 g, 206 mmol, 1.00 equiv.) in dry acetonitrile (120 mL, 2.28 mol, 11.0 equiv.) is cooled to −15 °C under argon. Tf_2O (52.0 mL, 309 mmol, 1.50 equiv.) is introduced into the dropping-funnel and added dropwise to the solution, keeping the temperature around −15 °C. The solution is then left at −15 °C for 18 h under argon in a freezer. The reaction is quenched by pouring the reaction media on ice (400 g), extracted with dichloromethane (3 × 80 mL), and washed with a saturated solution of NaHCO3 (3 × 40 mL). The organic phase is dried over $MgSO_4$, filtered, and concentrated under reduced pressure. To a solution of this crude product in acetonitrile (160 mL) and water (40 mL) is added $KMnO_4$ (32.6 g, 206 mmol, 1.00 equiv.) portionwise. The reaction is stirred at room temperature for 18 h and diluted with H2O (150 mL), and a saturated solution of $Na_2S_2O_4$ is added until complete discoloration of the solution. The product is extracted with dichloromethane (3 × 70 mL), and the organic phase is dried over $MgSO_4$, filtered, and concentrated under reduced pressure. The crude product is dissolved in acetonitrile (184 mL), and HCl 6 M (67.2 mL) is added. The reaction is stirred at room temperature for 18 h. Then, water (100 mL) is added and the organic phase is extracted with dichloromethane (3 × 50 mL), washed with a solution of saturated NaHCO3 (3 × 20 mL), dried over $MgSO_4$, filtered, and concentrated under reduced pressure. The product is filtered on silica (200 g) using petroleum ether/ethyl acetate 8/2 as eluent to afford the title compound as a white solid (**12**) (32.8 g, 157 mmol, 76%).

^{1}H NMR (300 MHz, $CDCl_3$) δ 8.15 (d, *J* = 7.5 Hz, 2H, Ar*H*), 7.84−7.72 (m, 1H, Ar*H*), 7.63 (dd, *J* = 8.5, 7.1 Hz, 2H, Ar*H*), 3.53 (s, br s, 1H, N*H*). ^{13}C NMR (75 MHz, $CDCl_3$) δ 135.6, 131.6, 130.7, 129.6, 121.0 (q, *J* = 333 Hz, CF_3). ^{19}F NMR (282 MHz, $CDCl_3$) δ −79.3 (s, 3F).

The characterization data corresponded to the reported values.[8]

1-iodo-2-(*S*-(trifluoromethyl)sulfonimidoyl)benzene (13)

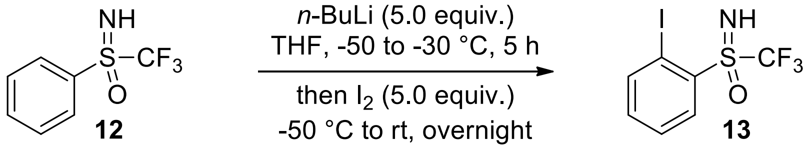

A solution of 2.5 M *n*-BuLi in hexane (96 mL, 0.24 mol, 5.0 equiv.) was added dropwise to a solution of (S-(trifluoromethyl)sulfonimidoyl)benzene **12** (10 g, 48 mmol, 1.0 equiv.) in freshly distilled THF (300 mL) at -50 °C. The reaction temperature was slowly increased to -30°C over 5 h. The reaction mixture was cooled to -50 °C, and solid I_2 (61 g, 0.24 mol, 5.0 equiv.) was added portion-wise. The reaction mixture was allowed to warm to room temperature overnight and subsequently quenched with a saturated aqueous NH_4Cl solution (200 mL). The aqueous layer was extracted with Et_2O (3 x 200 mL), dried with anhydrous $MgSO_4$, filtered, and concentrated. The residue was purified by flash column chromatography using toluene/MeOH (98/2) as eluent to give the title compound as a pale yellow solid **13** (15 g, 45 mmol, 94% yield).

^{1}H NMR (300 MHz, CD_3CN, 298 K): δ 8.40 (dd, *J* = 8.1, 1.3 Hz, 1H, Ar*H*), 8.32 (dd, *J* = 7.9, 0.9 Hz, 1H, Ar*H*), 7.72-7.67 (m, 1H, Ar*H*), 7.43 (td, *J* = 7.7, 1.5 Hz, 1H, Ar*H*), 4.98 (br. s, 1H, N*H*). ^{13}C NMR (75 MHz, CD_3CN, 298 K): δ 145.8, 137.2, 135.5, 135.2, 130.5, 121.8 (q, *J* = 333 Hz), 95.0. ^{19}F NMR (282 MHz, CD_3CN, 298 K): δ -75.4.

The characterization data corresponded to the reported values.[9]

[9] J. Kalim, T. Duhail, T.-N. Le, N. Vanthuyne, E. Anselmi, A. Togni, E. Magnier, *Chem. Sci.* **2019**, *10*, 10516–10523.

Semi-preparative separation of **13**:

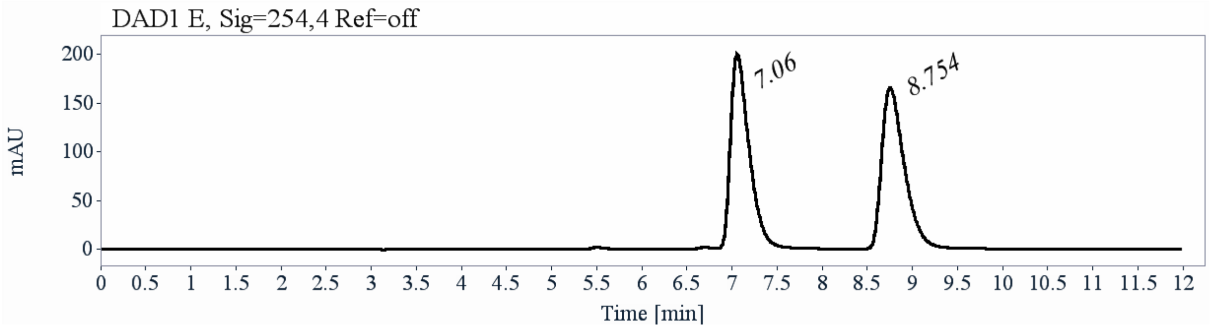

Figure S1: *HPLC trace of racemic **13**. Chromatographic conditions: Chiralpak AS-H (250 x 10 mm), n-hexane/isopropanol (80/20) as mobile phase, flow-rate = 5 mL/min, UV detection at 270 nm. Retention times: 7.06 min [(+)-**13**], 8.75 min [(–)-**13**].*

RT [min]	Area	Area%
7.06	2830	49.46
8.75	2891	50.54

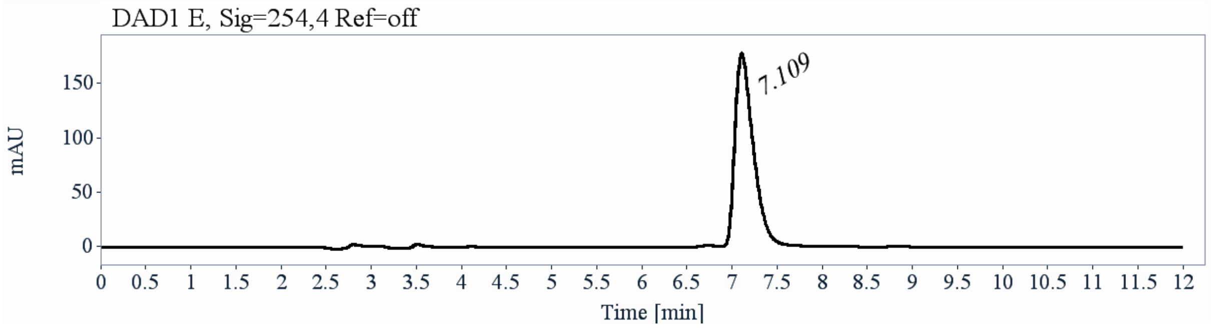

Figure S2: *HPLC trace of enantiopure (+)-**13**. Chromatographic conditions: Chiralpak AS-H (250 x 10 mm), n-hexane/isopropanol (80/20) as mobile phase, flow-rate = 5 mL/min, UV detection at 270 nm.*

RT [min]	Area	Area%
7.11	2474	100.00

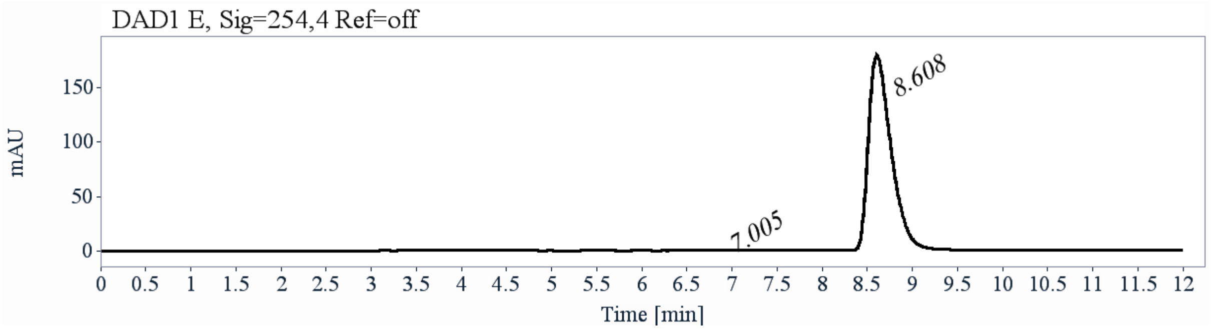

Figure S3: *HPLC trace of enantiopure (–)-**13**. Chromatographic conditions: Chiralpak AS-H (250 x 10 mm), n-hexane/isopropanol (80/20) as mobile phase, flow-rate = 5 mL/min, UV detection at 270 nm*

RT [min]	Area	Area%

7.00	7	0.23
8.61	3159	99.77

Other sulfoximines 15, 17a, 17b and 17c

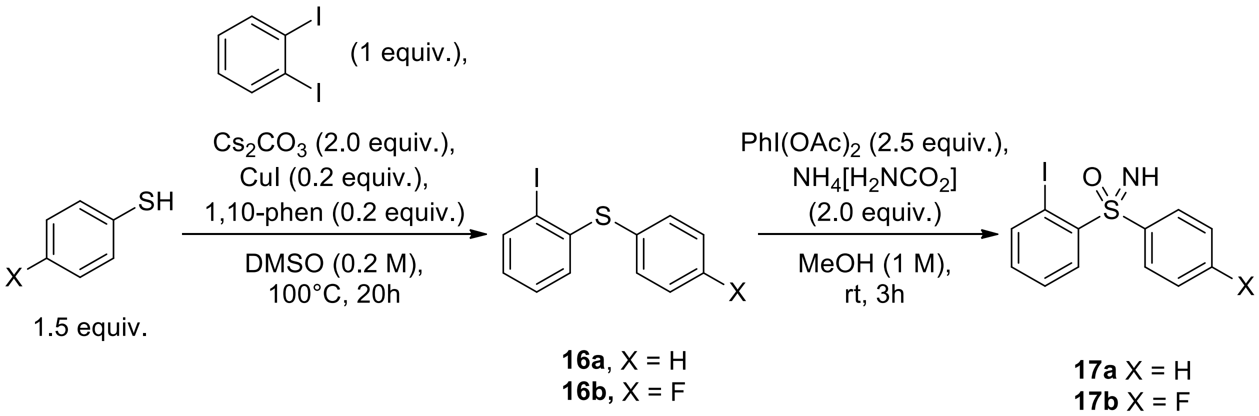

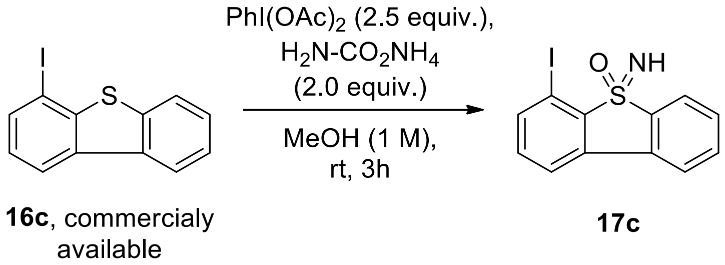

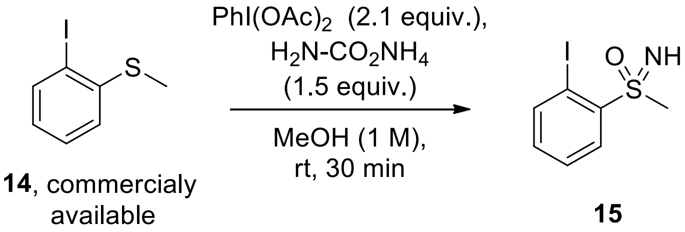

Sulfides 16a and 16b

Following a reported procedure,[10] a round bottom flask was charged with thiol aryl (1.5 equiv.), 1,2-diiodobenzene (1.0 equiv), Cs_2CO_3 (2.0 equiv.), CuI (0.2 equiv.), and 1,10-phenanthroline (0.2 equiv.) in DMSO (0.2 M) and heated at 100°C for 20h. The reaction mixture was then cooled at room temperature and diluted with water, extracted with EtOAc and dry over $MgSO_4$. The desired sulfide was isolated after column chromatography on silica gel (100% pentane) as a solid ; **16a** and **16b** were respectively obtained in 55% and 30% yield starting from thiophenol and 4-fluorothiophenol respectively. The characterization data corresponded to the reported values.[10]

Sulfoximines 15, 17a, 17b and 17c

Following a reported procedure,[11] a round bottom flask was charged with sulfide (1.0 equiv.) and MeOH (1 M). PIDA (2.5 equiv.) and ammonium carbamate (2.0 equiv.) were successively added, the flask well closed to maintain an ammoniac atmosphere and the reaction mixture stirred at room temperature for 3h (only 30 min were necessary for the transformation of **14** in **15**, and quantity of PIDA and ammonium carbamate could be reduced respectively to 1.5 equiv. and 2.1 equiv. in this case). Chromatography on silica gel (P/EtOAc 1/1) afforded the desired *ortho*-

[10] Y. Liu, H. Wang, X. Cao, Z. Feng, J.-P. Wan, *Synthesis* **2013**, *45*, 2977-2982.

[11] A. Tota, M. Zenzola, S. J. Chawner, S. St John-Campbell, C. Carlucci, G. Romanazzi, L. Degennaro, J. A. Bull, R. Luisi, *Chem. Commun.* **2017**, *53*, 348-351.

iodinated sulfoximine as solid. **15**, **17a**, **17b** and **17c** were respectively obtained in 60%, 70%, 63% and 60 yield starting from **14**, **16a**, **16b** and **16c** respectively.

1-iodo-2-(phenylsulfonimidoyl)benzene 17a

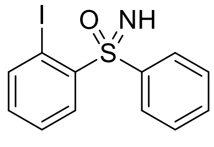

^{1}H NMR (300 MHz, CD_3CN) δ 8.46 (d, *J* = 8.0 Hz, 1H), 8.07 (d, *J* = 7.8 Hz, 1H), 7.96 (d, *J* = 7.6 Hz, 2H), 7.62 (t, *J* = 7.6 Hz, 2H), 7.54 (t, *J* = 8.1 Hz, 2H), 7.24 (t, *J* = 7.6 Hz, 1H), 3.25 – 2.20 (bs, 1H, N*H*). ^{13}C NMR (75 MHz, CD_3CN) δ 145.8, 144.2, 141.7, 134.6, 133.7, 131.6, 130.2, 129.9, 129.7, 129.6, 128.6, 93.9 (*C*-I). HRMS (ESI$^+$) m/z: [M + H]$^+$ Calcd for $C_{12}H_{11}INOS^+$ 343.9601; Found 343.9594.

1-(4-fluorophenylsulfonimidoyl)-2-iodobenzene 17b

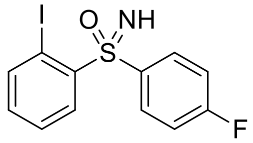

^{1}H NMR (300 MHz, $CDCl_3$) δ 8.41 (d, *J* = 8.0 Hz, 1H), 8.09 – 7.91 (m, 3H), 7.51 (t, *J* = 7.7 Hz, 1H), 7.14 (t, *J* = 8.5 Hz, 3H), 3.24 (s, 1H, *N*H). ^{13}C NMR (75 MHz, $CDCl_3$) δ 165.28 (d, *J* = 255.4 Hz, *C*-F), 144.7, 143.3, 135.90 (d, *J* = 3.1 Hz), 133.7, 132.1, 131.9, 130.6, 128.8, 116.1, 115.8, 93.2 (*C*-I). ^{19}F NMR (282 MHz, $CDCl_3$) δ -105.5 (td, *J* = 8.4, 4.2 Hz). HRMS (ESI$^+$) m/z: [M + H]$^+$ Calcd for $C_{12}H_{11}INOSF^+$ 361.9506; Found 361.9485.

5-imino-4-iododibenzo[b,d]thiophene 5-oxide 17c

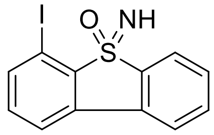

^{1}H NMR (300 MHz, $CDCl_3$) δ 7.92 (d, *J* = 7.7 Hz, 1H), 7.85 – 7.71 (m, 3H), 7.63 (t, *J* = 7.6 Hz, 1H), 7.54 (t, *J* = 7.7 Hz, 1H), 7.21 (dd, *J* = 8.5, 6.9 Hz, 1H), 3.81 (s, 1H). ^{13}C NMR (75 MHz, $CDCl_3$) δ 143.4, 140.0, 138.9, 133.9, 133.8, 133.3, 130.8, 130.8, 122.8, 121.4, 121.3, 85.7 (*C*-I). HRMS (ESI$^+$) m/z: [M + H]$^+$ Calcd for $C_{12}H_9INOS^+$ 341.9444; Found 341.9456.

1-iodo-2-(S-methylsulfonimidoyl)benzene 15

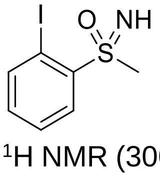

^{1}H NMR (300 MHz, $CDCl_3$) δ 8.26 (d, *J* = 7.9 Hz, 1H), 8.08 (d, *J* = 7.8 Hz, 1H), 7.50 (t, *J* = 7.6 Hz, 1H), 7.18 (t, *J* = 7.6 Hz, 1H), 3.25 (s, 3H), 2.85 (s, 1H). ^{13}C NMR (75 MHz, $CDCl_3$) δ 145.4, 143.0, 133.8, 130.5, 129.0, 93.2 (*C*-I), 42.5 (CH_3). HRMS (ESI$^+$) m/z: [M + H]$^+$ Calcd for $C_7H_9INOS^+$ 281.9444; Found 281.9446.

3. Preparation of hypervalent iodine reagents

3-(Tosylimino)-2,3-dihydro-1H-1λ3-benzo[d][1,2]iodazol-1-yl acetate (AcO-H,Ts-BZI, 15)

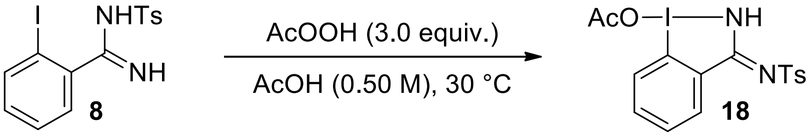

Following a slightly modified reported procedure,[12] in a round bottom flask, 2-iodo-N-tosylbenzimidamide **8** (2.0 g, 5.0 mmol, 1.0 equiv.) was dissolved in acetic acid (10 mL). The reaction mixture was cooled to 0 °C and peracetic acid (39% in acetic acid, 2.6 mL, 15 mmol, 3.0 equiv) was added dropwise to the aluminium foil covered flask. The reaction mixture was stirred at 30 °C for 2 h. The reaction was quenched by the addition of water (5 mL) and the precipitate was filtrated and washed with cold water (4X 5 mL) and with cold Et_2O (3X 5 mL). The precipitate was dried under vacuum and afforded the title compound as a white solid (**18**) (2.2 g, 4.8 mmol, 97% yield).

Mp > 166 °C (decomposition). ^{1}H NMR (400 MHz, DMSO-*d*$_6$) δ 11.97 (s, 1H, N*H*), 8.00 (d, *J* = 7.9 Hz, 1H, Ar*H*), 7.86-7.81 (m, 2H, Ar*H*), 7.73 (d, *J* = 8.2 Hz, 3H, Ar*H*), 7.22 (d, *J* = 8.0 Hz, 2H, Ar*H*), 2.32 (s, 3H, ArC*H*$_3$), 1.91 (s, 3H, OCC*H*$_3$). ^{13}C NMR (101 MHz, DMSO-*d*$_6$) δ 172.1, 169.0, 144.2, 142.5, 140.1, 132.3, 131.9, 129.6, 129.0, 128.5, 126.5, 121.0, 21.1, 20.9. IR (vmax, cm^{-1}) 3336 (w), 3064 (w), 2979 (w), 2920 (w), 1611 (w), 1576 (m), 1516 (s), 1363 (m), 1318 (s), 1158 (m), 1136 (s), 1082 (s), 1016 (m), 873 (s), 781 (s), 734 (m), 660 (s). HRMS (nanochip-ESI/LTQ-Orbitrap) m/z: [M + H]$^+$ Calcd for $C_{16}H_{16}IN_2O_4S^+$ 458.9870; Found 458.9854.

4-Methyl-N-(1-((triisopropylsilyl)ethynyl)-1,2-dihydro-3H-1λ3-benzo[d][1,2]iodazol-3-ylidene)benzenesulfonamide (TIPS-H,Ts-EBZI, 3)

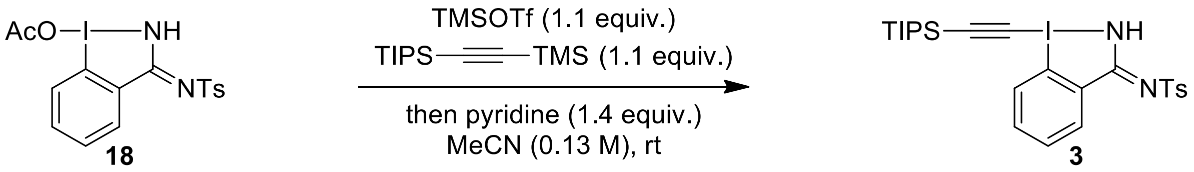

Following a reported procedure,[15] an oven-dried round-bottom flask equipped with magnetic stirring bar was charged with AcO-H,Ts-BZI **18** (1.0 g, 2.2 mmol, 1.0 equiv.) and MeCN (17 mL). TMS-OTf (0.43 mL, 2.4 mmol, 1.1 equiv.) was added to the solution and the resulting mixture was stirred at rt for 1 h. Then triisopropyl((trimethylsilyl)ethynyl)silane (0.61 g, 2.4 mmol, 1.1 equiv.) was added to the reaction mixture. After stirring at rt for 18 h, pyridine (0.25 mL, 3.1 mmol, 1.4 equiv.) was added and the reaction mixture was stirred vigorously for 1 h. The crude mixture was filtered and the precipitate washed with MeCN. The filtrate was concentrated under vacuum and purified by flash column chromatography using DCM/MeOH 99:1 as mobile phase to afford the title compound as a white solid (**3**) (0.36 g, 0.61 mmol, 28% yield).

R_f = 0.20 (DCM/MeOH 1%). Mp > 162 °C (decomposition). ^{1}H NMR (400 MHz, Chloroform-*d*) δ 8.55 (dd, *J* = 5.8, 3.2 Hz, 2H, N*H* and Ar*H*), 8.48-8.41 (m, 1H, Ar*H*), 7.86 (d, *J* = 8.0 Hz, 2H, Ar*H*), 7.71 (q, *J* = 5.2, 3.5 Hz, 2H, Ar*H*), 7.21 (d, *J* = 8.0 Hz, 2H, Ar*H*), 2.36 (s, 3H, C*H*$_3$), 1.13 (m, 21H, TIPS). ^{13}C NMR (101 MHz, Chloroform-*d*) δ 159.9, 142.0, 141.0, 134.1, 133.1, 131.4, 131.3, 129.2, 127.2, 126.4, 113.8, 110.2, 78.1, 21.6, 18.7, 11.4. IR (vmax, cm-1) 3329 (w), 2948 (w), 2870 (w), 1579 (w), 1517 (m), 1375 (m), 1271 (m), 1167 (w), 1135 (m), 1078 (m), 1002 (w), 876 (m), 812 (m), 773 (m), 662 (s). HRMS (ESI/QTOF) m/z: [M + H]$^+$ Calcd for $C_{25}H_{34}IN_2O_2SSi^+$ 581.1149; Found 581.1148.

N-(1-chloro-2-tosyl-1,2-dihydro-3H-1λ3-benzo[d][1,2]iodazol-3-ylidene)-4-methyl-

[12] V. V. Zhdankin, R. M. Arbit, B. J. Lynch, P. Kiprof, V. G. Young, *J. Org. Chem.* **1998**, *63*, 6590–6596.

-benzenesulfonamide (Cl-Ts-BZI, 19)

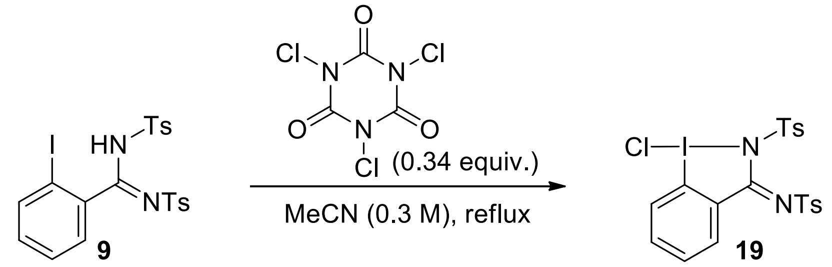

Following a reported procedure,[13] an oven-dried round-bottom flask equipped with magnetic stirring bar was charged under Ar with solid 2-iodo-N,N'-ditosylbenzimidamide (**9**) (1.1 g, 2.0 mmol, 1.0 equiv.) and anhydrous MeCN (7.0 mL) was added. The resulting stirred suspension was heated to 75 °C. A solution of trichloroisocyanuric acid (0.19 g, 0.80 mmol, 0.40 equiv, 1.2 equiv. in "Cl") in 1.0 mL of anhydrous MeCN was added dropwise. After addition was complete, the reaction mixture was refluxed for an additional 15 min. The reaction mixture was vacuum-filtered over a sintered-glass funnel and the precipitate was rinsed with additional hot MeCN (10−20 mL), the precipitate was air-dried. Then the precipitate was washed on a filter with DCM until only isocyanuric acid was left on the filter. The filtrate was concentrated under vacuum to afford the title compound (**19**) as a yellowish solid (1.1 g, 1.9 mmol, 93 %yield).

Mp > 223 °C (decomposition). ^{1}H NMR (400 MHz, Chloroform-*d*) δ 9.36 (dd, *J* = 7.4, 2.1 Hz, 1H, Ar*H*), 8.45-8.36 (m, 1H, Ar*H*), 7.88 (ddd, *J* = 6.8, 4.6, 1.7 Hz, 2H, Ar*H*), 7.84 (d, *J* = 8.3 Hz, 2H, Ar*H*), 7.40 (d, *J* = 8.1 Hz, 2H, Ar*H*), 7.32 (d, *J* = 8.3 Hz, 2H, Ar*H*), 6.94 (d, *J* = 8.1 Hz, 2H, Ar*H*), 2.53 (s, 3H, CH_3), 2.35 (s, 3H, CH_3). ^{13}C NMR (101 MHz, Chloroform-*d*) δ 153.5, 145.3, 143.3, 140.0, 136.7, 136.6, 134.0, 132.2, 130.7, 129.5, 129.4, 129.1, 128.5, 127.0, 114.9, 21.9, 21.8. IR (ν_{max}, cm^{-1}) 3086 (w), 1553 (m), 1444 (w), 1303 (m), 1256 (w), 1151 (m), 1082 (m), 1002 (w), 951 (w), 837 (m), 809 (m), 714 (s), 656 (m). Despite many attempts, the mass of the compound was not found by HRMS.

2-Tosyl-3-(tosylimino)-2,3-dihydro-1H-1λ^3-benzo[d][1,2]iodazol-1-yl acetate (AcO-Ts-BZI, 20)

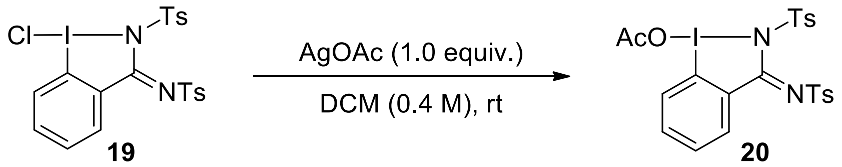

Following a reported procedure,[14] an oven-dried round-bottom flask equipped with magnetic stirring bar was charged under N_2 with N-(1-chloro-2-tosyl-1,2-dihydro-3H-1λ^3-benzo[d][1,2]iodazol-3-ylidene)-4-methylbenzenesulfonamide (**19**) (1.0 g, 1.7 mmol, 1.0 equiv.) and 8.0 mL of dry DCM was added. The flask was covered with aluminium foil to protect it from light. Silver acetate (0.28 g, 1.7 mmol, 1.0 equiv.) was added in one portion and the reaction mixture was stirred at rt for 22 h. The solution was filtered over a sintered-glass funnel and washed with DCM. The filtrate was concentrated under vacuum to afford the title compound (**20**) as a white solid (1.0 g,1.7 mmol, quant. yield).

Mp > 190 °C (decomposition). ^{1}H NMR (400 MHz, Methylene Chloride-d_2) δ 9.28 (dd, *J* = 8.0, 1.6 Hz, 1H, Ar*H*), 8.14 (dd, *J* = 8.3, 1.1 Hz, 1H, Ar*H*), 7.87 (td, *J* = 8.4, 7.9, 1.6 Hz, 1H, Ar*H*), 7.83-7.78 (m, 1H, Ar*H*), 7.75 (d, *J* = 8.3 Hz, 2H, Ar*H*), 7.41 (d, *J* = 8.0 Hz, 2H, Ar*H*), 7.33 (d, *J* = 8.4 Hz, 2H, Ar*H*), 6.97 (d, *J* = 8.1 Hz, 2H, Ar*H*), 2.53 (s, 3H, ArCH_3), 2.36 (s, 3H, ArCH_3), 2.25 (s, 3H, OCCH_3). ^{13}C NMR (101 MHz, Methylene Chloride-d_2) δ 176.7, 155.0, 145.7, 143.8, 140.7, 136.8, 136.7, 134.7, 131.8, 131.2, 130.9, 129.8(X2), 129.3, 127.1, 117.5, 22.0, 21.9, 21.0. IR (ν_{max}, cm^{-1}) 3076 (w), 2924 (w), 2751 (w), 1700 (w), 1540 (m), 1447 (w), 1326 (m), 1257 (m), 1209 (m),

[13] V. Matoušek, E. Pietrasiak, R. Schwenk, A. Togni, *J. Org. Chem.* **2013**, *78*, 6763–6768.

[14] Y. Kita, S. Akai, T. Okuno, M. Egi, T. Takada, H. Tohma, *HETEROCYCLES* **1996**, *42*, 47.

1149 (m), 1081 (m), 955 (w), 832 (m), 727 (s), 663 (s) HRMS (APPI/LTQ-Orbitrap) m/z: [M + H]+ Calcd for $C_{23}H_{22}IN_2O_6S_2^+$ 612.9959; Found 612.9946.

4-Methyl-N-(2-tosyl-1-((triisopropylsilyl)ethynyl)-1,2-dihydro-3H-1λ³-benzo[d][1,2] iodazol-3-ylidene)benzenesulfonamide (TIPS-Ts-EBZI, 4)

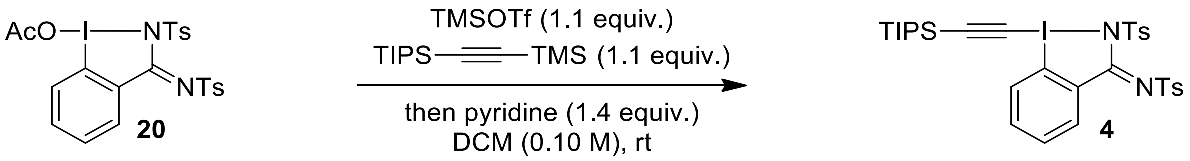

Following a reported procedure,[15] an oven-dried round-bottom flask equipped with magnetic stirring bar was charged with AcO-Ts-BZI **20** (0.61 g, 1.0 mmol, 1.0 equiv.) and DCM (7.7 mL). TMS-OTf (0.20 mL, 1.1 mmol, 1.1 equiv.) was added to the solution and the resulting mixture was stirred at rt for 1 h. Then triisopropyl((trimethylsilyl)ethynyl)silane (0.28 g, 1.1 mmol, 1.1 equiv.) was added to the reaction mixture. After stirring at rt for 3 h, pyridine (0.11 mL, 1.4 mmol, 1.4 equiv.) was added and the reaction mixture was stirred vigorously for 30 min. The crude mixture was filtered and the precipitate washed with DCM. The filtrate was concentrated under vacuum and purified by flash column chromatography using DCM/MeOH 99.5:0.5 as mobile phase to afford the title compound as a white solid (**4**) (0.53 g, 0.72 mmol, 72% yield). R_f = 0.32 (DCM/MeOH 1%). Mp > 192 °C (decomposition). ^{1}H NMR (400 MHz, Chloroform-d) δ 9.31 (dd, *J* = 7.9, 1.5 Hz, 1H, Ar*H*), 8.49 (dd, *J* = 8.4, 0.9 Hz, 1H, Ar*H*), 7.86-7.67 (m, 4H, Ar*H*), 7.35 (br d, *J* = 27.6 Hz, 4H, Ar*H*), 6.86 (br s, 2H, Ar*H*), 2.48 (br s, 3H, ArC*H*$_3$), 2.30 (br s, 3H, ArC*H*$_3$), 1.27-1.10 (m, 21H, TIPS). ^{13}C NMR (101 MHz, Chloroform-d) δ 153.0, 142.1, 141.1, 139.8, 135.6, 135.1, 134.3, 130.6, 130.5, 127.9 (X2), 127.2 (X2), 125.6, 114.3, 114.2, 67.8, 20.6 (X2), 17.5, 10.2. IR (vmax, cm^{-1}) 2949 (w), 2866 (w), 1523 (m), 1456 (w), 1351 (w), 1279 (m), 1147 (m), 1079 (s), 945 (w), 845 (m), 690 (s). HRMS (ESI/QTOF) m/z: [M + H]$^+$ Calcd for $C_{32}H_{40}IN_2O_4S_2Si^+$ 735.1238; Found 735.1248.

2-Tosyl-1-((triisopropylsilyl)ethynyl)-2,3-dihydro-1H-1λ³-benzo[d][1,2]iodazole (TIPS-Ts-EBz, 5)

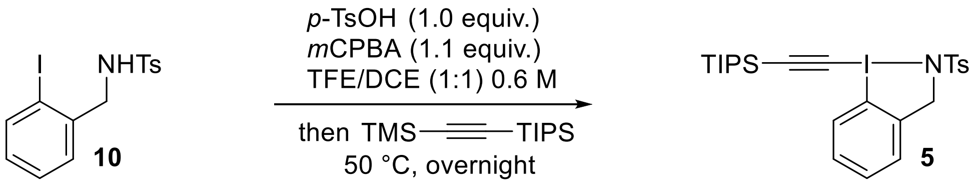

Following a slightly modified reported procedure,[16] in a sealed tube, N-(2-iodobenzyl)-4-methyl-benzenesulfonamide (1.0 g, 2.6 mmol, 1.0 equiv.), *p*-TsOH (0.49 g, 2.6 mmol, 1.0 equiv.) and *m*CPBA (0.64 g, 2.8 mmol, 1.1 equiv) were suspended in DCE:TFE (Ratio: 1:1, Volume: 4.4 mL) and heated up to 50 °C for 60 min. Triisopropyl(2-trimethylsilylethynyl)silane (0.92 g, 3.6 mmol, 1.4 equiv) was added at this temperature. The reaction mixture was stirred at this temperature overnight. The reaction mixture was concentrated under vacuum. The crude mixture was dissolved in 5 mL of DCM and washed with sat. $NaHCO_3$ (3 X 5 mL) and brine (5 mL). The organic layer was dried over $MgSO_4$ and the solvent were evaporated under vacuum. The crude mixture was purified by flash column chromatography using DCM/MeOH 99.5:0.5 as mobile phase to afford the title compound as a white solid (**5**) (0.71 g, 1.3 mmol, 49% yield).

R_f = 0.29 (DCM/MeOH 1%). Mp > 133 °C (decomposition). ^{1}H NMR (400 MHz, Chloroform-*d*) δ 8.31-8.25 (m, 1H, Ar*H*), 7.78-7.73 (m, 2H, Ar*H*), 7.49 (td, *J* = 7.3, 1.0 Hz, 1H, Ar*H*), 7.40-7.33 (m, 1H, Ar*H*), 7.25-7.17 (m, 3H, Ar*H*), 4.32 (s, 2H, ArC*H*$_2$N), 2.37 (s, 3H, ArC*H*$_3$), 1.13 (m, 21H, TIPS). ^{13}C NMR (101 MHz, Chloroform-*d*) δ 141.7, 139.1, 138.3, 130.8, 130.2, 129.7, 129.4, 128.8,

[15] V. V. Zhdankin, C. J. Kuehl, A. P. Krasutsky, J. T. Bolz, A. J. Simonsen, *J. Org. Chem.* **1996**, *61*, 6547–6551.

[16] D. P. Hari, P. Caramenti, L. Schouwey, M. Chang, S. Nicolai, D. Bachert, T. Wright, C. Orella, J. Waser, *Org. Process Res. Dev.* **2020**, *24*, 106–110.

127.0, 111.3, 109.9, 75.8, 46.5, 21.6, 18.7, 11.4. IR (ν_{max}, cm^{-1}) 2940 (m), 2864 (m), 1461 (m), 1273 (s), 1253 (m), 1151 (s), 1135 (s), 1087 (s), 999 (m), 915 (s), 883 (m), 811 (m), 747 (s), 674 (s). HRMS (ESI/QTOF) m/z: $[M + H]^+$ Calcd for $C_{25}H_{35}INO_2SSi^+$ 568.1197; Found 568.1202.

3-(Trifluoromethyl)-1-((triisopropylsilyl)ethynyl)-1H-1λ³,3λ⁴-benzo[d][1,3,2]iodathiazole 3-oxide (TIPS-CF$_3$-EBS, 6)

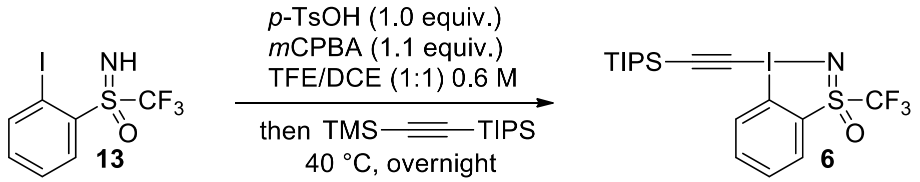

Following a slightly modified reported procedure,[16] in a sealed tube 1-iodo-2-(S-(trifluoromethyl)sulfonimidoyl)benzene **13** (1.0 g, 3.0 mmol, 1.0 equiv.), *p*-TsOH (0.57 g, 3.0 mmol, 1.0 equiv.) and *m*CPBA (0.74 g, 3.3 mmol, 1.1 equiv.) were suspended in DCE:TFE (Ratio: 1:1, Volume: 5.0 mL) and heated up to 40 °C for 60 min. Triisopropyl((trimethylsilyl)ethynyl)silane (1.1 g, 4.2 mmol, 1.4 equiv.) was added at this temperature. The reaction mixture was stirred at this temperature overnight. pyridine (0.34 mL, 4.2 mmol, 1.4 equiv.) was added and the mixture was stirred vigorously for 10 min. The reaction mixture was concentrated under vacuum. The crude mixture was dissolved in 5 mL of DCM and washed with sat. $NaHCO_3$ (3 X 5 mL) and brine (5 mL). The organic layer was dried over $MgSO_4$ and the solvent were evaporated under vacuum. The crude mixture was purified by flash column chromatography using DCM/MeOH 99:1 as mobile phase to afford the title compound as a slightly yellow solid (**6**) (1.2 g, 2.2 mmol, 75 % yield).

R_f = 0.21 (DCM/MeOH 1%). Mp > 125 °C (decomposition). ^1H NMR (400 MHz, Chloroform-*d*) δ 8.79-8.74 (m, 1H, Ar*H*), 8.22 (d, *J* = 7.3 Hz, 1H, Ar*H*), 7.95-7.84 (m, 2H, Ar*H*), 1.15 (m, 21H, TIPS). ^{13}C NMR (101 MHz, Chloroform-*d*) δ 135.5, 132.4, 131.4, 129.9, 129.0, 122.6 (q, *J* = 337.2 Hz), 120.9, 110.8, 76.1, 18.7, 11.4. ^{19}F NMR (376 MHz, Chloroform-*d*) δ -77.8. IR (ν_{max}, cm^{-1}) 3076 (m), 2945 (m), 2867 (m), 1559 (m), 1464 (m), 1434 (m), 1301 (s), 1254 (m), 1189 (s), 1169 (s), 1096 (m), 1063 (s), 883 (m), 690 (s). HRMS (ESI/QTOF) m/z: $[M + H]^+$ Calcd for $C_{18}H_{26}F_3INOSSi^+$ 516.0496; Found 516.0494.

For the enantiomer **6** prepared from (-)-**13**: $[\alpha]_\lambda^{20}$ ($CHCl_3$, c = 0.5, λ = 589 nm): +12

HPLC trace of racemic **13**, Chiralpak IB 80:20 Hexane/iPrOH, 1.0 ml/min, 31 min

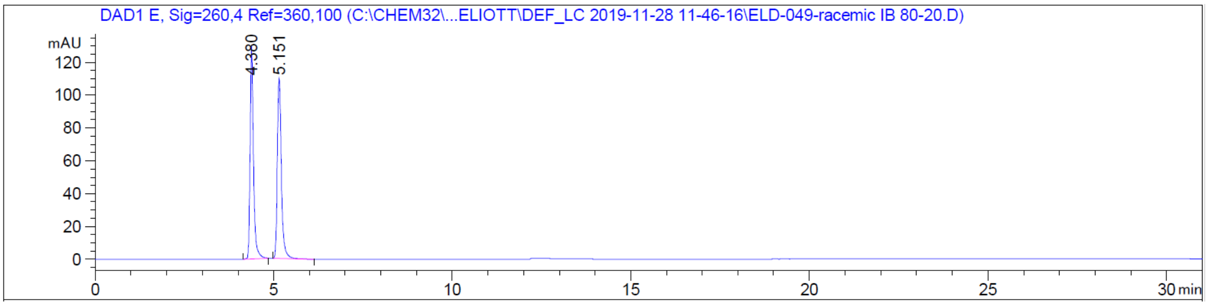

Peak #	RetTime [min]	Type	Width [min]	Area [mAU*s]	Height [mAU]	Area %
1	4.380	BB	0.0987	858.55957	130.60844	49.9553
2	5.151	BB	0.1197	860.09583	109.41880	50.0447

HPLC trace of enantiopure (+)-**6** obtained from (-)-**13**, Chiralpak IB 80:20 Hexane/iPrOH, 1.0 ml/min, 31 min

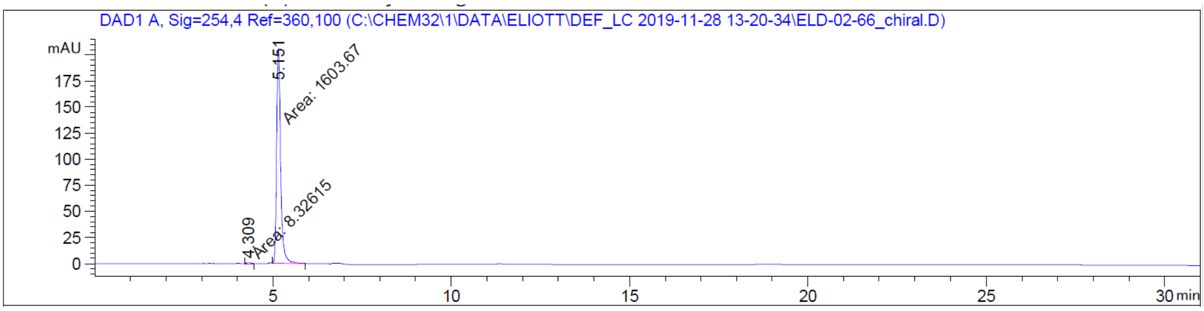

Peak #	RetTime [min]	Type	Width [min]	Area [mAU*s]	Height [mAU]	Area %
1	4.309	MM	0.1568	8.32615	8.84966e-1	0.5165
2	5.151	MM	0.1303	1603.67468	205.11935	99.4835

4. Reactivity investigation

Alkynylation of β-ketoesters

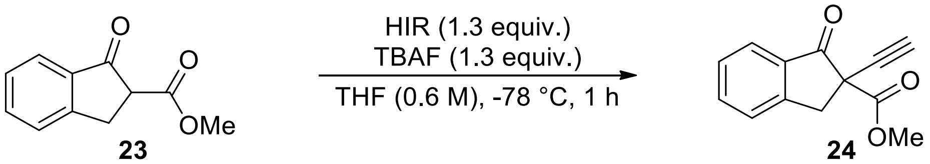

23 24

Following a reported procedure,[17] a solution of methyl 1-oxo-2,3-dihydro-1H-indene-2-carboxylate (20 mg, 0.10 mmol, 1.0 equiv.) and hypervalent iodine reagent (0.13 mmol, 1.30 equiv.) in dry THF (1.7 mL) was stirred at -78 °C for 5 min under nitrogen. After this period of time, TBAF (0.13 mL, 0.13 mmol, 1.3 equiv.) was added and the mixture was vigorously stirred at -78 °C. The reaction was monitored by TLC analysis (Pentane/EtOAc, 4:1, UV and p-anisaldehyde) and was complete at -78 °C in 1 hour. The reaction was quenched by addition of water at rt and aqueous layer was extracted with DCM. The combined layer were dried over MgSO4 and concentrated under vacuum. The crude mixture was purified by PrepTLC (Pentane/EtOAc 5/1) to afford the title **24** as a yellow oil.

Starting from TIPS-H,Ts-EBZI **3** (75 mg, 0.13 mmol, 1.3 equiv.), **24** was not observed.

Starting from TIPS-Ts-EBz **5** (74 mg, 0.13 mmol, 1.3 equiv.), **24** could not be isolated from the degradation products of **5** (79% NMR yield using CH_2Br_2 as internal standard).

Starting from racemic TIPS-EBS **6** (67 mg, 0.13 mmol, 1.3 equiv.), **24** (20 mg, 90 µmol, 90% yield) was obtained as a racemic mixture.

Starting from enantiopure TIPS-EBS **(+)-6** (67 mg, 0.13 mmol, 1.3 equiv.), **24** (20 mg, 90 µmol, 90% yield) was obtained as a racemic mixture.

Starting from TIPS-EBX **21** (56 mg, 0.13 mmol, 1.3 equiv.), **24** (21 mg, 0.10 mmol, quant. Yield) was obtained.

Starting from TIPS-Ts-EBZI **4** (96 mg, 0.13 mmol, 1.3 equiv.), **24** (21 mg, 0.10 mmol, quant. Yield) was obtained.

[17] D. Fernández González, J. P. Brand, R. Mondière, J. Waser, *Advanced Synthesis & Catalysis* **2013**, *355*, 1631–1639.

^{1}H NMR (400 MHz, Chloroform-*d*) δ 7.83 (d, *J* = 7.7 Hz, 1H, Ar*H*), 7.70-7.64 (m, 1H, Ar*H*), 7.50 (d, *J* = 7.7 Hz, 1H, Ar*H*), 7.47-7.41 (m, 1H, Ar*H*), 3.94 (d, *J* = 17.1 Hz, 1H, ArCH_2), 3.80 (s, 3H, OCH_3), 3.52 (d, *J* = 17.1 Hz, 1H, ArCH_2), 2.42 (s, 1H, CC*H*).

The ^{1}H NMR data corresponds to literature data.[17]

Alkynylation of thiol

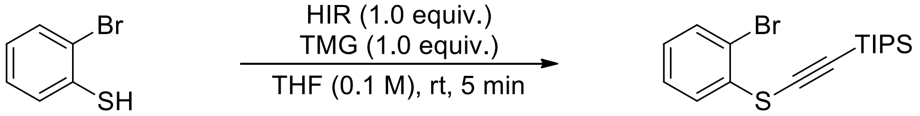

25 **26**

Following a reported procedure,[18] a 5 mL microwave vial was charged with a magnetic stir bar, 2-bromobenzenethiol (12 µL, 0.10 mmol, 1.0 equiv.), 1,1,3,3-tetramethylguanidine (13 µL, 0.10 mmol, 1.0 equiv.) and THF (1.0 mL). After stirring the resulting solution for 5 minutes at room temperature, hypervalent iodine reagent (0.10 mmol, 1.0 equiv.) was added as a solid in one portion. The resulting reaction mixture was stirred with an open flask for 5 minutes at room temperature. Next, the mixture was diluted with water (10 mL) and extracted with EtOAc (3 x 10 mL). The combined organic layers were dried over $MgSO_4$, filtered and concentrated in vacuo. The reaction mixture was purified by PrepTLC using pentane as mobile phase affording **26** as a clear colourless oil.

Starting from TIPS-H,Ts-EBZI **3** (58 mg, 0.10 mmol, 1.0 equiv.), **26** was not observed.

Starting from TIPS-Ts-EBz **5** (57 mg, 0.10 mmol, 1.0 equiv.), **26** (8 mg, 2 µmol, 22% yield)

Starting from racemic TIPS-EBS **6** (52 mg, 0.10 mmol, 1.0 equiv.), **26** (27 mg, 73 µmol, 73% yield) was obtained.

Starting from TIPS-Ts-EBZI **4** (74 mg, 0.10 mmol, 1.0 equiv.), **26** (17 mg, 46 µmol, 46% yield) was obtained.

^{1}H NMR (400 MHz, Chloroform-*d*) δ 7.75 (dd, *J* = 8.0, 1.1 Hz, 1H, Ar*H*), 7.51-7.47 (m, 1H, Ar*H*), 7.40-7.31 (m, 1H, Ar*H*), 7.08 (td, *J* = 7.9, 1.3 Hz, 1H, Ar*H*), 1.13 (m, 21H, TIPS).

The ^{1}H NMR data corresponds to literature data.[18]

Decarboxylative-alkynylation of proline

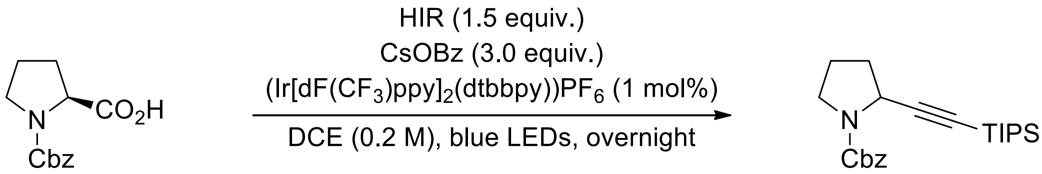

27 **28**

Following a reported procedure,[19] dry degassed DCE (0.50 mL) was added in a flame dried 1.5 mL test tube containing a teflon coated stirring bar, Cbz-Pro-OH (25 mg, 0.10 mmol, 1.0 equiv.), hypervalent iodine reagent (0.15 mmol, 1.5 equiv.), CsOBz (76 mg, 0.30 mmol, 3.0 equiv.) and Ir(dF(CF_3)ppy)$_2$(dtbbpy)PF_6 (1.1 mg, 1.0 µmol, 0.01 equiv.) under N_2. The reaction mixture was again degassed by bubbling N_2 inside the test tube via syringe for 5 min before being irradiated using blue light LEDs for 22 h at rt. The reaction mixture was filtered over celite, eluting with ethyl acetate, and evaporated under reduced pressure. The crude product was purified by preparative TLC (Pentane/Ethyl Acetate 8/2) directly without any further work-up.

Starting from TIPS-H,Ts-EBZI **3** (87 mg, 0.15 mmol, 1.5 equiv.), **28** was not observed.

[18] R. Frei, J. Waser, *J. Am. Chem. Soc.* **2013**, *135*, 9620–9623.
[19] F. Le Vaillant, T. Courant, J. Waser, *Angew. Chem. Int. Ed.* **2015**, *54*, 11200–11204.

Starting from TIPS-Ts-EBz **5** (85 mg, 0.15 mmol, 1.5 equiv.), **28** (4 mg, 10 µmol, 10% yield)

Starting from racemic TIPS-EBS **6** (77 mg, 0.15 mmol, 1.5 equiv.), **28** (4 mg, 10 µmol, 10% yield) was obtained.

Starting from TIPS-Ts-EBZI **4** (110 mg, 0.150 mmol, 1.5 equiv.), **28** was not observed.

^{1}H NMR (400 MHz, Chloroform-*d*) δ 7.45-7.27 (m, 5H, Ar*H*), 5.17 (s, 2H, OCH_2Ph), 4.66-4.52 (m, 1H, CbzNC*H*CC), 3.63-3.49 (m, 1H, CbzNCH_2), 3.48-3.29 (m, 1H, CbzNCH_2), 2.24-1.86 (m, 4H, CbzNCH_2CH_2CH_2CH), 1.03 (s, 21H, TIPS).

The ^{1}H NMR data corresponds to literature data.[19]

5. X-ray crystallographic data

5.1. Single Crystal X-Ray Diffraction for the compound 3

Crystals of the compound **3** were obtained from slow evaporation of a DCM solution.

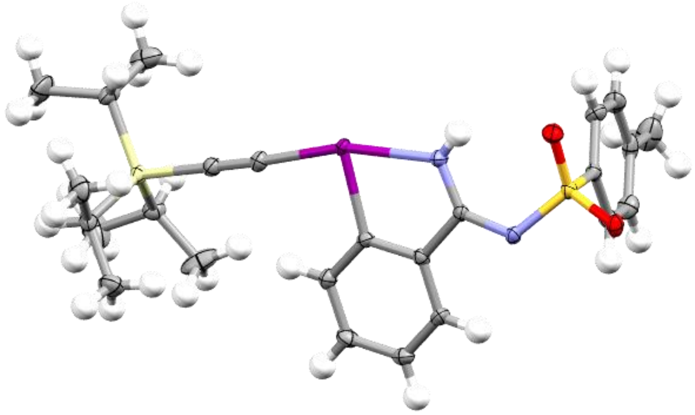

Table 1. Crystal data and structure refinement for **3.**

Identification code	CCDC 2072273	
Empirical formula	$C_{25}H_{33}IN_2O_2SSi$	
Formula weight	580.58	
Temperature	140.02(18) K	
Wavelength	0.71073 Å	
Crystal system	Monoclinic	
Space group	$P2_1/c$	
Unit cell dimensions	a = 11.6983(5) Å	α= 90°.
	b = 16.7909(6) Å	β= 99.201(3)°.
	c = 13.5441(4) Å	γ = 90°.
Volume	2626.17(17) $Å^3$	
Z	4	
Density (calculated)	1.468 Mg/m^3	
Absorption coefficient	1.368 mm^{-1}	
F(000)	1184	
Crystal size	0.297 x 0.108 x 0.044 mm^3	
Θ range for data collection	2.426 to 32.970°.	
Index ranges	$-17 \leq h \leq 17, -25 \leq k \leq 25, -20 \leq l \leq 20$	
Reflections collected	15787	
Independent reflections	15787	
Completeness to θ = 25.242°	99.2 %	
Absorption correction	Gaussian	
Max. and min. transmission	1.000 and 0.698	
Refinement method	Full-matrix least-squares on F^2	
Data / restraints / parameters	15787 / 0 / 303	
Goodness-of-fit on F^2	0.924	
Final R indices [I > 2σ(I)]	$R_1 = 0.0417, wR_2 = 0.0763$	
R indices (all data)	$R_1 = 0.0646, wR_2 = 0.0802$	

Largest diff. peak and hole	1.534 and -0.833 e.Å^{-3}

Table 2. Atomic coordinates (x 10^4) and equivalent isotropic displacement parameters (Å^{2}x 10^3) for **3**. U(eq) is defined as one third of the trace of the orthogonalized U^{ij} tensor.

	x	y	z	U(eq)
I(1)	4059(1)	2950(1)	6360(1)	16(1)
S(1)	1736(1)	2795(1)	9443(1)	16(1)
Si(1)	7029(1)	3841(1)	4150(1)	17(1)
O(1)	1211(2)	2191(1)	8754(1)	23(1)
O(2)	1646(2)	2685(1)	10488(1)	21(1)
N(1)	2987(2)	2750(2)	7636(2)	22(1)
N(2)	3082(2)	2946(1)	9413(1)	16(1)
C(1)	5013(2)	3517(2)	7631(2)	16(1)
C(2)	6015(2)	3944(2)	7589(2)	21(1)
C(3)	6553(2)	4326(2)	8449(2)	23(1)
C(4)	6095(2)	4284(2)	9324(2)	23(1)
C(5)	5097(2)	3847(2)	9359(2)	20(1)
C(6)	4541(2)	3450(2)	8512(2)	16(1)
C(7)	3445(2)	3003(2)	8518(2)	15(1)
C(8)	5319(2)	3297(2)	5501(2)	21(1)
C(9)	5986(2)	3516(2)	4973(2)	20(1)
C(10)	7205(3)	4952(2)	4302(2)	23(1)
C(11)	7308(4)	5251(2)	5379(2)	47(1)
C(12)	8213(3)	5265(2)	3814(3)	50(1)
C(13)	8391(2)	3251(2)	4531(2)	23(1)
C(14)	8986(3)	3415(2)	5605(2)	35(1)
C(15)	8145(3)	2356(2)	4405(2)	33(1)
C(16)	6306(3)	3558(2)	2846(2)	23(1)
C(17)	7122(3)	3523(3)	2070(2)	49(1)
C(18)	5256(3)	4088(2)	2489(2)	38(1)
C(19)	1077(2)	3713(2)	9046(2)	16(1)
C(20)	1459(2)	4405(2)	9560(2)	21(1)
C(21)	976(3)	5128(2)	9247(2)	26(1)
C(22)	101(3)	5183(2)	8419(2)	25(1)
C(23)	-274(3)	4485(2)	7928(2)	28(1)
C(24)	209(2)	3751(2)	8223(2)	23(1)
C(25)	-413(3)	5974(2)	8067(2)	40(1)

Table 3. Bond lengths [Å] and angles [°] for **3.**

I(1)-C(8)	2.102(3)
I(1)-C(1)	2.122(2)
I(1)-N(1)	2.317(2)
S(1)-O(1)	1.4467(19)
S(1)-O(2)	1.4471(18)
S(1)-N(2)	1.601(2)
S(1)-C(19)	1.768(3)
Si(1)-C(9)	1.863(3)
Si(1)-C(13)	1.877(3)
Si(1)-C(10)	1.884(3)
Si(1)-C(16)	1.892(3)
N(1)-C(7)	1.299(3)
N(2)-C(7)	1.351(3)
C(1)-C(2)	1.382(4)
C(1)-C(6)	1.398(3)
C(2)-C(3)	1.390(4)
C(3)-C(4)	1.379(4)
C(4)-C(5)	1.386(4)
C(5)-C(6)	1.395(3)
C(6)-C(7)	1.487(4)
C(8)-C(9)	1.196(4)
C(10)-C(11)	1.529(4)
C(10)-C(12)	1.534(4)
C(13)-C(14)	1.534(4)
C(13)-C(15)	1.535(4)
C(16)-C(17)	1.530(4)
C(16)-C(18)	1.531(4)
C(19)-C(24)	1.384(4)
C(19)-C(20)	1.391(4)
C(20)-C(21)	1.378(4)
C(21)-C(22)	1.395(4)
C(22)-C(23)	1.384(4)
C(22)-C(25)	1.504(4)
C(23)-C(24)	1.388(4)
C(8)-I(1)-C(1)	89.99(10)
C(8)-I(1)-N(1)	164.74(9)
C(1)-I(1)-N(1)	74.76(9)
O(1)-S(1)-O(2)	116.81(12)
O(1)-S(1)-N(2)	114.36(12)

O(2)-S(1)-N(2)	105.72(11)
O(1)-S(1)-C(19)	107.15(12)
O(2)-S(1)-C(19)	108.19(12)
N(2)-S(1)-C(19)	103.72(12)
C(9)-Si(1)-C(13)	106.79(13)
C(9)-Si(1)-C(10)	107.15(13)
C(13)-Si(1)-C(10)	114.83(13)
C(9)-Si(1)-C(16)	104.33(12)
C(13)-Si(1)-C(16)	110.92(13)
C(10)-Si(1)-C(16)	112.05(13)
C(7)-N(1)-I(1)	116.46(19)
C(7)-N(2)-S(1)	119.06(17)
C(2)-C(1)-C(6)	122.1(2)
C(2)-C(1)-I(1)	122.61(18)
C(6)-C(1)-I(1)	115.31(18)
C(1)-C(2)-C(3)	118.6(2)
C(4)-C(3)-C(2)	120.7(3)
C(3)-C(4)-C(5)	120.1(2)
C(4)-C(5)-C(6)	120.6(2)
C(5)-C(6)-C(1)	117.9(2)
C(5)-C(6)-C(7)	121.7(2)
C(1)-C(6)-C(7)	120.3(2)
N(1)-C(7)-N(2)	131.2(2)
N(1)-C(7)-C(6)	112.9(2)
N(2)-C(7)-C(6)	115.9(2)
C(9)-C(8)-I(1)	176.1(2)
C(8)-C(9)-Si(1)	179.1(3)
C(11)-C(10)-C(12)	110.5(3)
C(11)-C(10)-Si(1)	114.9(2)
C(12)-C(10)-Si(1)	111.7(2)
C(14)-C(13)-C(15)	109.3(2)
C(14)-C(13)-Si(1)	113.7(2)
C(15)-C(13)-Si(1)	110.6(2)
C(17)-C(16)-C(18)	111.3(3)
C(17)-C(16)-Si(1)	114.6(2)
C(18)-C(16)-Si(1)	111.4(2)
C(24)-C(19)-C(20)	120.0(3)
C(24)-C(19)-S(1)	120.9(2)
C(20)-C(19)-S(1)	119.08(19)
C(21)-C(20)-C(19)	119.9(2)
C(20)-C(21)-C(22)	121.2(3)
C(23)-C(22)-C(21)	117.7(3)

C(23)-C(22)-C(25)	121.0(3)
C(21)-C(22)-C(25)	121.2(3)
C(22)-C(23)-C(24)	122.0(3)
C(19)-C(24)-C(23)	119.1(3)

Symmetry transformations used to generate equivalent atoms:

Table 4. Anisotropic displacement parameters ($Å^2$x 10^3)for **3**. The anisotropic displacement factor exponent takes the form: $-2\pi^2[h^2a^{*2}U^{11} + ... + 2 h k a^* b^* U^{12}]$

	U^{11}	U^{22}	U^{33}	U^{23}	U^{13}	U^{12}
I(1)	17(1)	19(1)	11(1)	0(1)	4(1)	-1(1)
S(1)	16(1)	19(1)	15(1)	1(1)	4(1)	0(1)
Si(1)	20(1)	18(1)	16(1)	0(1)	6(1)	-2(1)
O(1)	23(1)	22(1)	24(1)	-3(1)	4(1)	-5(1)
O(2)	22(1)	25(1)	18(1)	5(1)	9(1)	2(1)
N(1)	18(1)	33(2)	15(1)	-3(1)	5(1)	-8(1)
N(2)	16(1)	22(1)	12(1)	2(1)	4(1)	1(1)
C(1)	16(1)	16(1)	15(1)	-2(1)	0(1)	1(1)
C(2)	22(2)	23(2)	17(1)	1(1)	6(1)	-1(1)
C(3)	18(2)	25(2)	27(1)	1(1)	2(1)	-6(1)
C(4)	22(2)	25(2)	20(1)	-3(1)	-2(1)	-2(1)
C(5)	20(1)	26(2)	13(1)	-1(1)	2(1)	0(1)
C(6)	14(1)	17(1)	16(1)	2(1)	2(1)	1(1)
C(7)	15(1)	17(1)	13(1)	0(1)	3(1)	2(1)
C(8)	22(2)	23(2)	17(1)	0(1)	5(1)	0(1)
C(9)	21(1)	21(2)	17(1)	-1(1)	4(1)	0(1)
C(10)	26(2)	20(2)	23(1)	0(1)	3(1)	-3(1)
C(11)	84(3)	23(2)	29(2)	-7(1)	-7(2)	0(2)
C(12)	46(2)	29(2)	81(3)	3(2)	27(2)	-11(2)
C(13)	20(1)	24(2)	27(1)	-2(1)	10(1)	-1(1)
C(14)	26(2)	40(2)	35(2)	-4(1)	-3(1)	6(2)
C(15)	32(2)	26(2)	40(2)	-3(1)	9(2)	6(1)
C(16)	31(2)	23(2)	16(1)	-2(1)	5(1)	-4(1)
C(17)	57(3)	72(3)	19(2)	-3(2)	14(2)	-7(2)
C(18)	47(2)	32(2)	31(2)	-5(1)	-10(2)	4(2)
C(19)	14(1)	19(2)	15(1)	2(1)	4(1)	0(1)
C(20)	21(2)	23(2)	19(1)	1(1)	0(1)	-2(1)
C(21)	33(2)	20(2)	27(2)	-2(1)	9(1)	-1(1)
C(22)	30(2)	27(2)	22(1)	7(1)	15(1)	9(1)
C(23)	25(2)	36(2)	21(1)	6(1)	2(1)	8(1)
C(24)	24(2)	28(2)	16(1)	-2(1)	1(1)	-1(1)
C(25)	53(2)	34(2)	35(2)	12(2)	14(2)	18(2)

Table 5. Hydrogen bonds for **3** [Å and °].

D-H...A	d(D-H)	d(H...A)	d(D...A)	<(DHA)
N(1)-H(1)...O(1)	0.80(4)	2.32(3)	2.915(3)	132(3)

Symmetry transformations used to generate equivalent atoms:

5.2. Single Crystal X-Ray Diffraction for the compound 4

Crystals of the compound **4** were obtained from slow evaporation of a DCM solution.

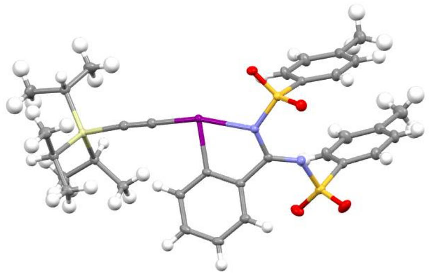

Table 1. Crystal data and structure refinement for **4**.

Identification code	CCDC 2072274	
Empirical formula	$C_{32}H_{39}IN_2O_4S_2Si$	
Formula weight	734.76	
Temperature	100.01(11) K	
Wavelength	0.71073 Å	
Crystal system	Triclinic	
Space group	*P*-1	
Unit cell dimensions	a = 11.3588(3) Å	α= 112.064(3)°.
	b = 11.8051(3) Å	β= 98.385(2)°.
	c = 14.2418(4) Å	γ = 105.326(2)°.
Volume	1641.46(8) $Å^3$	
Z	2	
Density (calculated)	1.487 Mg/m^3	
Absorption coefficient	1.178 mm^{-1}	
F(000)	752	
Crystal size	0.545 x 0.213 x 0.093 mm^3	
Θ range for data collection	2.553 to 32.959°.	
Index ranges	$-15 \leq h \leq 16, -17 \leq k \leq 13, -15 \leq l \leq 20$	
Reflections collected	19613	
Independent reflections	10888 [R_{int} = 0.0177]	

Completeness to θ = 25.242°	99.9 %
Absorption correction	Gaussian
Max. and min. transmission	1.000 and 0.407
Refinement method	Full-matrix least-squares on F^2
Data / restraints / parameters	10888 / 0 / 387
Goodness-of-fit on F^2	1.052
Final R indices [I > 2σ(I)]	R_1 = 0.0244, wR_2 = 0.0561
R indices (all data)	R_1 = 0.0276, wR_2 = 0.0576
Largest diff. peak and hole	0.617 and -0.488 e.Å^{-3}

Table 2. Atomic coordinates (x 10^4) and equivalent isotropic displacement parameters ($Å^2$x 10^3) for **4**. U(eq) is defined as one third of the trace of the orthogonalized U^{ij} tensor.

	x	y	z	U(eq)
I(1)	6733(1)	4017(1)	4753(1)	13(1)
S(2)	5691(1)	5825(1)	3530(1)	15(1)
S(3)	6788(1)	3414(1)	721(1)	19(1)
Si(1)	7675(1)	1355(1)	6694(1)	14(1)
O(1)	5449(1)	6165(1)	4556(1)	19(1)
O(2)	6128(1)	6855(1)	3227(1)	21(1)
O(3)	7338(1)	4211(1)	234(1)	29(1)
O(4)	7344(1)	2475(1)	792(1)	27(1)
N(1)	6734(1)	5121(1)	3607(1)	15(1)
N(2)	6656(1)	4381(1)	1835(1)	16(1)
C(1)	7134(1)	4505(1)	2770(1)	15(1)
C(2)	8164(1)	4066(1)	3131(1)	14(1)
C(3)	9201(1)	4063(1)	2708(1)	18(1)
C(4)	10170(1)	3723(2)	3111(1)	19(1)
C(5)	10107(1)	3340(2)	3917(1)	20(1)
C(6)	9103(1)	3360(1)	4372(1)	17(1)
C(7)	8172(1)	3753(1)	3982(1)	15(1)
C(8)	7100(1)	2987(2)	5568(1)	17(1)
C(9)	7313(1)	2391(1)	6059(1)	17(1)
C(10)	6457(1)	1116(2)	7434(1)	19(1)
C(11)	5093(2)	539(2)	6732(2)	29(1)
C(12)	6663(2)	309(2)	8030(2)	32(1)
C(13)	9312(1)	2304(1)	7634(1)	18(1)
C(14)	9337(2)	3559(2)	8520(1)	26(1)
C(15)	10342(1)	2609(2)	7086(1)	23(1)
C(16)	7560(1)	-204(1)	5569(1)	17(1)
C(17)	8217(2)	-1016(2)	5929(1)	25(1)
C(18)	8052(2)	-4(2)	4672(1)	22(1)
C(19)	4265(1)	4664(1)	2589(1)	16(1)
C(20)	3690(2)	3498(2)	2639(1)	23(1)
C(21)	2539(2)	2638(2)	1916(1)	24(1)
C(22)	1935(2)	2923(2)	1155(1)	21(1)
C(23)	2521(2)	4097(2)	1126(1)	25(1)
C(24)	3679(2)	4966(2)	1836(1)	23(1)
C(25)	656(2)	1999(2)	401(1)	31(1)
C(26)	5179(1)	2536(2)	13(1)	18(1)
C(27)	4572(2)	1386(2)	66(2)	26(1)

C(28)	3352(2)	614(2)	-579(2)	28(1)
C(29)	2736(2)	979(2)	-1270(1)	22(1)
C(30)	3339(2)	2165(2)	-1277(1)	26(1)
C(31)	4557(2)	2948(2)	-635(1)	23(1)
C(32)	1448(2)	88(2)	-2020(2)	33(1)

Table 3. Bond lengths [Å] and angles [°] for **4**.

I(1)-C(8)	2.0460(15)
I(1)-C(7)	2.1233(14)
I(1)-N(1)	2.4425(12)
S(2)-O(2)	1.4319(10)
S(2)-O(1)	1.4510(11)
S(2)-N(1)	1.6289(12)
S(2)-C(19)	1.7643(15)
S(3)-O(3)	1.4366(12)
S(3)-O(4)	1.4401(13)
S(3)-N(2)	1.6248(13)
S(3)-C(26)	1.7627(15)
Si(1)-C(9)	1.8644(15)
Si(1)-C(10)	1.8830(15)
Si(1)-C(13)	1.8839(15)
Si(1)-C(16)	1.8886(16)
N(1)-C(1)	1.3558(18)
N(2)-C(1)	1.3001(19)
C(1)-C(2)	1.499(2)
C(2)-C(7)	1.3937(19)
C(2)-C(3)	1.3983(19)
C(3)-C(4)	1.386(2)
C(4)-C(5)	1.388(2)
C(5)-C(6)	1.393(2)
C(6)-C(7)	1.387(2)
C(8)-C(9)	1.205(2)
C(10)-C(11)	1.530(2)
C(10)-C(12)	1.532(2)
C(13)-C(14)	1.534(2)
C(13)-C(15)	1.537(2)
C(16)-C(18)	1.539(2)
C(16)-C(17)	1.540(2)
C(19)-C(24)	1.387(2)
C(19)-C(20)	1.392(2)
C(20)-C(21)	1.386(2)
C(21)-C(22)	1.392(2)
C(22)-C(23)	1.389(2)
C(22)-C(25)	1.510(2)
C(23)-C(24)	1.387(2)
C(26)-C(31)	1.384(2)
C(26)-C(27)	1.386(2)

C(27)-C(28)	1.387(2)
C(28)-C(29)	1.386(2)
C(29)-C(30)	1.392(2)
C(29)-C(32)	1.508(2)
C(30)-C(31)	1.387(2)
C(8)-I(1)-C(7)	93.03(6)
C(8)-I(1)-N(1)	165.51(5)
C(7)-I(1)-N(1)	72.48(5)
O(2)-S(2)-O(1)	117.76(7)
O(2)-S(2)-N(1)	111.63(6)
O(1)-S(2)-N(1)	102.12(6)
O(2)-S(2)-C(19)	108.56(7)
O(1)-S(2)-C(19)	106.92(7)
N(1)-S(2)-C(19)	109.48(7)
O(3)-S(3)-O(4)	117.65(8)
O(3)-S(3)-N(2)	107.30(7)
O(4)-S(3)-N(2)	114.31(7)
O(3)-S(3)-C(26)	108.37(7)
O(4)-S(3)-C(26)	106.74(7)
N(2)-S(3)-C(26)	101.04(7)
C(9)-Si(1)-C(10)	106.76(7)
C(9)-Si(1)-C(13)	106.42(7)
C(10)-Si(1)-C(13)	110.61(7)
C(9)-Si(1)-C(16)	104.94(7)
C(10)-Si(1)-C(16)	113.47(7)
C(13)-Si(1)-C(16)	113.96(7)
C(1)-N(1)-S(2)	122.81(10)
C(1)-N(1)-I(1)	109.18(9)
S(2)-N(1)-I(1)	119.32(6)
C(1)-N(2)-S(3)	127.42(11)
N(2)-C(1)-N(1)	121.61(13)
N(2)-C(1)-C(2)	129.39(13)
N(1)-C(1)-C(2)	108.99(12)
C(7)-C(2)-C(3)	117.23(13)
C(7)-C(2)-C(1)	119.87(12)
C(3)-C(2)-C(1)	122.65(13)
C(4)-C(3)-C(2)	120.58(14)
C(3)-C(4)-C(5)	120.56(14)
C(4)-C(5)-C(6)	120.29(14)
C(7)-C(6)-C(5)	117.92(14)
C(6)-C(7)-C(2)	123.22(13)

C(6)-C(7)-I(1)	119.73(11)
C(2)-C(7)-I(1)	116.93(10)
C(9)-C(8)-I(1)	179.13(14)
C(8)-C(9)-Si(1)	174.04(14)
C(11)-C(10)-C(12)	110.60(13)
C(11)-C(10)-Si(1)	112.83(11)
C(12)-C(10)-Si(1)	112.60(11)
C(14)-C(13)-C(15)	110.53(12)
C(14)-C(13)-Si(1)	111.13(11)
C(15)-C(13)-Si(1)	113.24(11)
C(18)-C(16)-C(17)	109.17(13)
C(18)-C(16)-Si(1)	114.08(10)
C(17)-C(16)-Si(1)	112.99(11)
C(24)-C(19)-C(20)	120.23(14)
C(24)-C(19)-S(2)	118.93(11)
C(20)-C(19)-S(2)	120.71(11)
C(21)-C(20)-C(19)	119.06(14)
C(20)-C(21)-C(22)	121.60(15)
C(23)-C(22)-C(21)	118.27(15)
C(23)-C(22)-C(25)	120.77(15)
C(21)-C(22)-C(25)	120.93(15)
C(24)-C(23)-C(22)	121.05(15)
C(19)-C(24)-C(23)	119.78(14)
C(31)-C(26)-C(27)	120.60(14)
C(31)-C(26)-S(3)	120.40(11)
C(27)-C(26)-S(3)	118.89(12)
C(26)-C(27)-C(28)	119.31(15)
C(29)-C(28)-C(27)	120.94(15)
C(28)-C(29)-C(30)	118.87(15)
C(28)-C(29)-C(32)	120.49(15)
C(30)-C(29)-C(32)	120.62(15)
C(31)-C(30)-C(29)	120.73(15)
C(26)-C(31)-C(30)	119.43(14)

Symmetry transformations used to generate equivalent atoms:

Table 4. Anisotropic displacement parameters ($Å^2$x 10^3)for **4**. The anisotropic displacement factor exponent takes the form: $-2\pi^2[h^2a^{*2}U^{11} + ... + 2 h k a^* b^* U^{12}]$

	U^{11}	U^{22}	U^{33}	U^{23}	U^{13}	U^{12}
I(1)	13(1)	15(1)	14(1)	8(1)	5(1)	7(1)
S(2)	17(1)	15(1)	18(1)	10(1)	8(1)	8(1)
S(3)	17(1)	22(1)	16(1)	7(1)	6(1)	6(1)
Si(1)	15(1)	14(1)	15(1)	8(1)	4(1)	7(1)
O(1)	20(1)	20(1)	20(1)	9(1)	9(1)	10(1)
O(2)	25(1)	17(1)	28(1)	14(1)	10(1)	9(1)
O(3)	26(1)	36(1)	22(1)	13(1)	11(1)	2(1)
O(4)	23(1)	28(1)	25(1)	4(1)	4(1)	14(1)
N(1)	16(1)	17(1)	17(1)	10(1)	7(1)	9(1)
N(2)	18(1)	18(1)	16(1)	9(1)	6(1)	8(1)
C(1)	13(1)	14(1)	18(1)	9(1)	6(1)	5(1)
C(2)	14(1)	14(1)	16(1)	6(1)	5(1)	6(1)
C(3)	16(1)	18(1)	19(1)	7(1)	7(1)	5(1)
C(4)	16(1)	19(1)	22(1)	6(1)	7(1)	8(1)
C(5)	15(1)	18(1)	24(1)	7(1)	4(1)	9(1)
C(6)	16(1)	17(1)	19(1)	8(1)	4(1)	8(1)
C(7)	13(1)	15(1)	18(1)	7(1)	5(1)	6(1)
C(8)	18(1)	19(1)	18(1)	9(1)	6(1)	8(1)
C(9)	18(1)	18(1)	17(1)	8(1)	6(1)	8(1)
C(10)	21(1)	19(1)	20(1)	10(1)	8(1)	8(1)
C(11)	18(1)	37(1)	32(1)	16(1)	9(1)	8(1)
C(12)	35(1)	40(1)	35(1)	28(1)	16(1)	16(1)
C(13)	18(1)	17(1)	18(1)	8(1)	3(1)	7(1)
C(14)	25(1)	23(1)	21(1)	4(1)	6(1)	6(1)
C(15)	18(1)	22(1)	27(1)	9(1)	6(1)	7(1)
C(16)	16(1)	17(1)	18(1)	8(1)	3(1)	7(1)
C(17)	29(1)	21(1)	26(1)	8(1)	1(1)	14(1)
C(18)	22(1)	22(1)	20(1)	7(1)	8(1)	10(1)
C(19)	16(1)	18(1)	18(1)	10(1)	7(1)	9(1)
C(20)	21(1)	22(1)	29(1)	16(1)	2(1)	8(1)
C(21)	21(1)	22(1)	31(1)	14(1)	3(1)	7(1)
C(22)	20(1)	26(1)	17(1)	8(1)	5(1)	11(1)
C(23)	28(1)	34(1)	20(1)	17(1)	5(1)	13(1)
C(24)	27(1)	27(1)	22(1)	16(1)	7(1)	11(1)
C(25)	27(1)	35(1)	23(1)	9(1)	1(1)	10(1)
C(26)	19(1)	19(1)	16(1)	7(1)	5(1)	6(1)
C(27)	22(1)	29(1)	33(1)	21(1)	4(1)	7(1)

C(28)	22(1)	24(1)	38(1)	16(1)	5(1)	5(1)
C(29)	20(1)	23(1)	17(1)	4(1)	3(1)	7(1)
C(30)	26(1)	33(1)	22(1)	15(1)	4(1)	13(1)
C(31)	25(1)	22(1)	24(1)	13(1)	6(1)	9(1)
C(32)	24(1)	34(1)	26(1)	2(1)	0(1)	7(1)

5.3. Single Crystal X-Ray Diffraction for the compound 5

Crystals of the compound **5** were obtained from slow evaporation of a DCM solution.

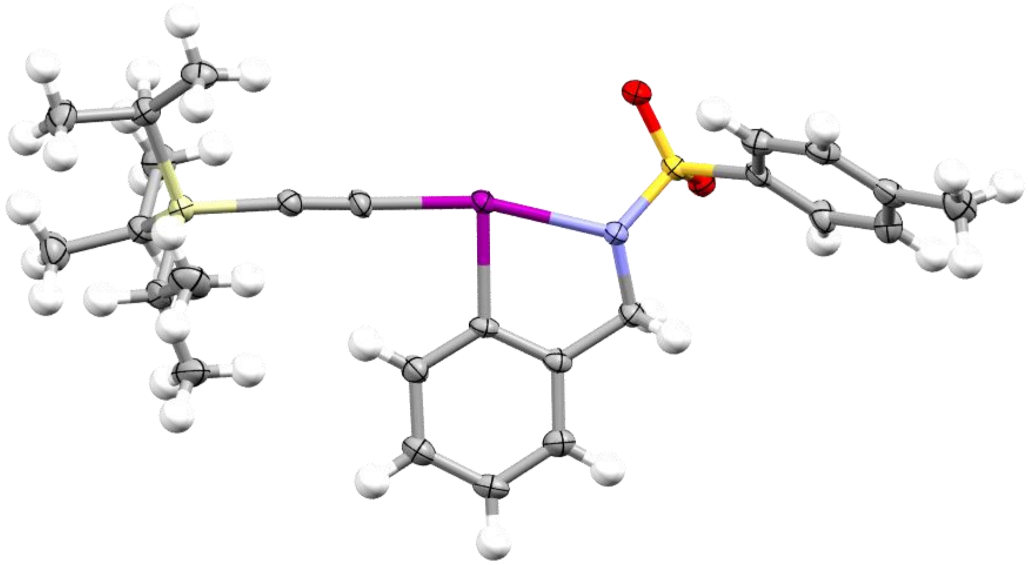

Compound	TIPS-Ts-EBz
Identification code	CCDC 2072275
Formula	$C_{25}H_{34}INO_2SSi$
$D_{calc.}$/ g cm^{-3}	1.476
μ/mm^{-1}	11.226
Formula Weight	567.58
Colour	clear colourless
Shape	plate
Size/mm^3	0.75×0.30×0.07
T/K	100.00(10)
Crystal System	triclinic
Space Group	$P\bar{1}$
a/Å	8.3150(5)
b/Å	9.5571(4)
c/Å	16.2727(8)
α/°	95.596(4)
β/°	91.566(5)
γ/°	96.709(4)
V/Å^3	1277.18(11)
Z	2
Z'	1
Wavelength/Å	1.54184
Radiation type	Cu $K\alpha$
Θ_{min}/°	2.731
Θ_{max}/°	76.215
Measured Refl's.	10120
Ind't Refl's	5160
Refl's with I > 2(I)	5087
R_{int}	0.0292
Parameters	288
Restraints	0
Largest Peak/e Å^{-3}	2.075
Deepest Hole/e Å^{-3}	-1.586
GooF	1.085
wR_2 (all data)	0.1245
wR_2	0.1242
R_1 (all data)	0.0444
R_1	0.0442

Table 1: Fractional Atomic Coordinates (×10^4) and Equivalent Isotropic Displacement Parameters (Å^2×10^3) for **5**. U_{eq} is defined as 1/3 of the trace of the orthogonalised U_{ij}.

Atom	x	y	z	U_{eq}
I1	5659.6(2)	2441.2(2)	4424.6(2)	19.74(12)
S1	6563.5(11)	1786.3(9)	6388.6(5)	22.85(19)
Si1	3192.6(12)	2995.4(10)	1545.0(6)	22.9(2)
O1	5870(3)	2555(3)	7073.0(16)	27.5(5)
O2	5584(4)	547(3)	5977.2(17)	29.8(6)
N1	7138(4)	2744(3)	5681.9(19)	22.6(6)
C1	6827(4)	4574(3)	4508(2)	20.8(6)
C2	6638(5)	5404(4)	3865(2)	25.8(7)
C3	7477(5)	6760(4)	3928(2)	28.0(7)
C4	8486(5)	7237(4)	4611(3)	26.7(8)
C5	8614(5)	6405(4)	5252(2)	26.2(7)
C6	7770(4)	5044(3)	5220(2)	21.1(6)
C7	7864(4)	4185(4)	5943(2)	23.8(7)
C8	8338(4)	1225(4)	6798(2)	23.1(7)
C9	9033(5)	119(4)	6385(2)	29.6(8)
C10	10461(5)	-269(4)	6687(3)	29.7(8)
C11	11259(5)	438(4)	7404(3)	29.2(8)
C12	10549(5)	1550(4)	7812(2)	30.3(8)
C13	9095(5)	1948(4)	7515(2)	27.3(7)

Atom	x	y	z	U_{eq}
C14	12835(5)	48(5)	7728(3)	38.4(9)
C15	4632(5)	2656(4)	3257(2)	23.9(7)
C16	4059(4)	2735(4)	2576(2)	23.3(7)
C17	4870(5)	3896(4)	953(2)	29.8(8)
C18	5596(6)	5332(5)	1398(3)	42.0(10)
C19	6218(6)	2971(6)	757(3)	43.6(10)
C20	1595(5)	4218(4)	1754(2)	27.8(7)
C21	936(6)	4788(5)	986(3)	38.8(9)
C22	206(5)	3545(5)	2246(3)	35.8(9)
C23	2305(5)	1213(4)	1019(2)	27.6(7)
C24	1980(6)	1261(5)	88(3)	38.8(9)
C25	3313(5)	2(4)	1158(3)	33.2(8)

Table 2: Anisotropic Displacement Parameters (×10^4) for **5**. The anisotropic displacement factor exponent takes the form: $-2\pi^2[h^2a^{*2} \times U_{11}+ ... +2hka^* \times b^* \times U_{12}]$

Atom	U_{11}	U_{22}	U_{33}	U_{23}	U_{13}	U_{12}
I1	22.37(16)	16.17(16)	21.29(16)	3.46(9)	-0.64(9)	3.98(9)
S1	26.1(4)	18.5(4)	24.4(4)	5.9(3)	-2.8(3)	2.6(3)
Si1	24.4(5)	22.5(5)	22.0(4)	4.8(3)	-1.9(3)	2.0(4)
O1	25.7(12)	29.6(13)	28.5(12)	5.9(10)	-0.7(9)	6.2(10)
O2	36.5(14)	19.7(12)	32.4(13)	7.1(10)	-6.6(11)	-2.6(11)
N1	27.8(15)	16.3(15)	23.1(14)	4.4(11)	-5.2(11)	0.4(12)
C1	22.8(15)	12.6(14)	27.5(16)	3.5(12)	3.6(12)	2.3(12)
C2	31.8(18)	22.9(17)	23.0(16)	4.1(13)	1.4(13)	3.5(14)
C3	32.6(19)	22.5(17)	30.5(17)	7.4(13)	6.8(14)	5.2(14)
C4	28.4(18)	17.7(17)	34.3(19)	4.0(14)	9.6(14)	0.9(14)
C5	23.7(17)	22.6(18)	32.1(18)	1.7(13)	2.8(13)	1.9(14)
C6	21.2(15)	15.5(15)	27.5(16)	3.2(12)	2.7(12)	5.2(12)
C7	26.5(17)	19.0(16)	25.9(16)	3.0(12)	-4.0(12)	4.0(13)
C8	29.1(17)	17.4(15)	24.4(16)	8.3(12)	0.6(13)	4.0(13)
C9	34(2)	20.7(17)	34.2(19)	2.2(14)	2.2(14)	1.8(15)
C10	29.5(18)	18.9(16)	42(2)	6.7(14)	4.0(15)	4.5(14)
C11	25.8(18)	26.4(19)	39(2)	15.1(15)	7.8(15)	6.2(15)
C12	30.8(19)	35(2)	26.1(17)	4.8(14)	-1.8(14)	7.7(16)
C13	32.3(19)	24.3(18)	27.3(17)	3.2(13)	-1.4(14)	11.7(15)
C14	27.2(19)	43(2)	50(2)	16.7(19)	4.1(17)	11.8(17)
C15	29.8(18)	21.3(17)	22.1(16)	6.7(12)	-1.7(13)	5.9(14)
C16	23.9(17)	19.4(16)	27.2(17)	5.0(13)	-1.0(13)	3.1(13)
C17	34.9(19)	30.7(19)	23.0(16)	6.6(14)	0.9(14)	-3.2(16)
C18	52(3)	36(2)	34(2)	3.4(17)	2.0(18)	-15(2)
C19	34(2)	47(3)	51(3)	13(2)	13.8(19)	1.0(19)
C20	30.5(18)	23.6(17)	29.2(17)	3.5(13)	-3.4(14)	3.1(14)
C21	43(2)	37(2)	39(2)	9.6(17)	-8.6(17)	13.4(18)
C22	32(2)	38(2)	38(2)	3.3(17)	3.1(16)	7.9(17)
C23	27.3(17)	26.2(18)	28.3(17)	1.5(13)	-1.3(13)	0.8(14)
C24	45(2)	38(2)	31(2)	-2.1(16)	-8.9(16)	3.3(19)
C25	37(2)	24.3(18)	38(2)	0.6(15)	3.4(16)	4.1(16)

Table 3: Bond Lengths in Å for **5**.

Atom	Atom	Length/Å
I1	N1	2.333(3)
I1	C1	2.143(3)
I1	C15	2.101(4)
S1	O1	1.445(3)
S1	O2	1.450(3)
S1	N1	1.588(3)
S1	C8	1.763(4)
Si1	C16	1.858(4)
Si1	C17	1.889(4)
Si1	C20	1.885(4)

Atom	Atom	Length/Å
Si1	C23	1.888(4)
N1	C7	1.456(5)
C1	C2	1.389(5)
C1	C6	1.388(5)
C2	C3	1.391(5)
C3	C4	1.381(6)
C4	C5	1.381(6)
C5	C6	1.399(5)
C6	C7	1.505(5)
C8	C9	1.389(5)

Atom	Atom	Length/Å
C8	C13	1.390(5)
C9	C10	1.377(6)
C10	C11	1.399(6)
C11	C12	1.397(6)
C11	C14	1.499(5)
C12	C13	1.397(5)
C15	C16	1.206(6)
C17	C18	1.537(6)
C17	C19	1.528(6)
C20	C21	1.524(5)
C20	C22	1.539(6)
C23	C24	1.539(5)
C23	C25	1.537(6)

Table 4: Bond Angles in ° for **5**.

Atom	Atom	Atom	Angle/°
C1	I1	N1	75.34(12)
C15	I1	N1	165.99(14)
C15	I1	C1	90.77(14)
O1	S1	O2	117.43(18)
O1	S1	N1	113.44(17)
O1	S1	C8	105.69(16)
O2	S1	N1	106.12(16)
O2	S1	C8	107.65(17)
N1	S1	C8	105.81(17)
C16	Si1	C17	107.69(17)
C16	Si1	C20	105.63(16)
C16	Si1	C23	108.48(17)
C20	Si1	C17	109.67(18)
C20	Si1	C23	112.00(17)
C23	Si1	C17	113.02(17)
S1	N1	I1	118.84(17)
C7	N1	I1	115.4(2)
C7	N1	S1	117.0(2)
C2	C1	I1	120.1(3)
C6	C1	I1	116.5(2)
C6	C1	C2	123.4(3)
C1	C2	C3	118.2(3)
C4	C3	C2	119.9(3)
C5	C4	C3	120.5(4)
C4	C5	C6	121.4(4)
C1	C6	C5	116.5(3)
C1	C6	C7	123.1(3)
C5	C6	C7	120.4(3)
N1	C7	C6	108.2(3)
C9	C8	S1	120.4(3)
C9	C8	C13	119.8(3)
C13	C8	S1	119.7(3)
C10	C9	C8	120.1(4)
C9	C10	C11	121.6(4)
C10	C11	C14	122.1(4)
C12	C11	C10	117.7(4)
C12	C11	C14	120.2(4)
C11	C12	C13	121.2(4)
C8	C13	C12	119.5(3)
C16	C15	I1	177.7(3)
C15	C16	Si1	175.9(3)
C18	C17	Si1	112.2(3)
C19	C17	Si1	113.0(3)
C19	C17	C18	109.8(4)
C21	C20	Si1	114.3(3)
C21	C20	C22	110.8(4)
C22	C20	Si1	111.9(3)
C24	C23	Si1	111.9(3)
C25	C23	Si1	114.2(3)
C25	C23	C24	109.7(3)

Table 5: Torsion Angles in ° for **5**.

Atom	Atom	Atom	Atom	Angle/°
I1	N1	C7	C6	-12.9(3)
I1	C1	C2	C3	-177.1(3)
I1	C1	C6	C5	176.1(2)
I1	C1	C6	C7	-5.9(4)
S1	N1	C7	C6	-160.1(2)
S1	C8	C9	C10	176.8(3)
S1	C8	C13	C12	-176.5(3)
O1	S1	N1	I1	-108.64(19)
O1	S1	N1	C7	37.4(3)
O1	S1	C8	C9	160.5(3)
O1	S1	C8	C13	-23.2(3)
O2	S1	N1	I1	21.8(2)
O2	S1	N1	C7	167.8(3)
O2	S1	C8	C9	34.3(3)
O2	S1	C8	C13	-149.5(3)
N1	S1	C8	C9	-78.9(3)
N1	S1	C8	C13	97.4(3)
C1	C2	C3	C4	1.1(5)

Atom	Atom	Atom	Atom	Angle/˚
C1	C6	C7	N1	12.7(4)
C2	C1	C6	C5	-3.0(5)
C2	C1	C6	C7	175.0(3)
C2	C3	C4	C5	-3.0(6)
C3	C4	C5	C6	1.9(6)
C4	C5	C6	C1	1.0(5)
C4	C5	C6	C7	-177.0(3)
C5	C6	C7	N1	-169.4(3)
C6	C1	C2	C3	2.0(5)
C8	S1	N1	I1	135.94(18)
C8	S1	N1	C7	-78.0(3)
C8	C9	C10	C11	-0.8(6)
C9	C8	C13	C12	-0.2(6)
C9	C10	C11	C12	0.6(6)
C9	C10	C11	C14	-178.5(4)
C10	C11	C12	C13	-0.2(6)
C11	C12	C13	C8	0.0(6)
C13	C8	C9	C10	0.6(6)
C14	C11	C12	C13	178.9(4)
C16	Si1	C17	C18	-59.2(4)
C16	Si1	C17	C19	65.6(3)
C16	Si1	C20	C21	169.5(3)
C16	Si1	C20	C22	-63.4(3)
C16	Si1	C23	C24	-164.5(3)
C16	Si1	C23	C25	-39.1(3)
C17	Si1	C20	C21	53.7(3)
C17	Si1	C20	C22	-179.2(3)
C17	Si1	C23	C24	-45.2(3)
C17	Si1	C23	C25	80.2(3)
C20	Si1	C17	C18	55.3(4)
C20	Si1	C17	C19	-179.9(3)
C20	Si1	C23	C24	79.3(3)
C20	Si1	C23	C25	-155.3(3)
C23	Si1	C17	C18	-179.0(3)
C23	Si1	C17	C19	-54.2(4)
C23	Si1	C20	C21	-72.6(3)
C23	Si1	C20	C22	54.5(3)

Table 6: Hydrogen Fractional Atomic Coordinates (×10[4]) and Equivalent Isotropic Displacement Parameters (Å[2]×10[3]) for **5**. U_{eq} is defined as 1/3 of the trace of the orthogonalised U_{ij}.

Atom	x	y	z	U_{eq}
H2	5954.44	5054.22	3394.55	31
H3	7356.31	7356.14	3502.18	34
H4	9097.72	8146.18	4639.93	32
H5	9288.2	6763.8	5723.98	31
H7A	9009.03	4185.94	6127.55	29
H7B	7274.23	4596.55	6409.93	29
H9	8521.76	-370.31	5892.56	36
H10	10915.91	-1034.68	6400.92	36
H12	11063.88	2045.12	8301.91	36
H13	8626.86	2706.67	7799.49	33
H14A	13686.96	840.17	7701.63	58
H14B	13119.99	-787.91	7390.94	58
H14C	12726.62	-164.31	8302.27	58
H17	4378.91	4085.64	413.54	36
H18A	6444.77	5767.9	1065.52	63
H18B	6064.27	5189.39	1938.58	63
H18C	4743.45	5953.45	1473.81	63
H19A	6762.1	2798.19	1272.8	65
H19B	7004.68	3456.4	410.97	65
H19C	5751.08	2066.13	461.47	65
H20	2131.77	5057.23	2115.85	33
H21A	1826.8	5306.45	716.81	58
H21B	132.3	5426.75	1145.54	58
H21C	424.56	3997.48	601.93	58
H22A	-426.74	2770.52	1895.13	54
H22B	-497.47	4262.59	2424.2	54
H22C	656.04	3173.86	2731.48	54
H23	1229.46	972.47	1264.11	33
H24A	1270.27	1988.27	3.06	58
H24B	1455.41	336.8	-155.98	58
H24C	3009.04	1489.38	-177.66	58
H25A	4312.61	120.67	853.49	50
H25B	2685.3	-906.78	960.36	50
H25C	3585.51	18.5	1749.08	50

5.4. Single Crystal X-Ray Diffraction for the compound 6

Crystals of the compound **6** were obtained from slow evaporation of a DCM solution.

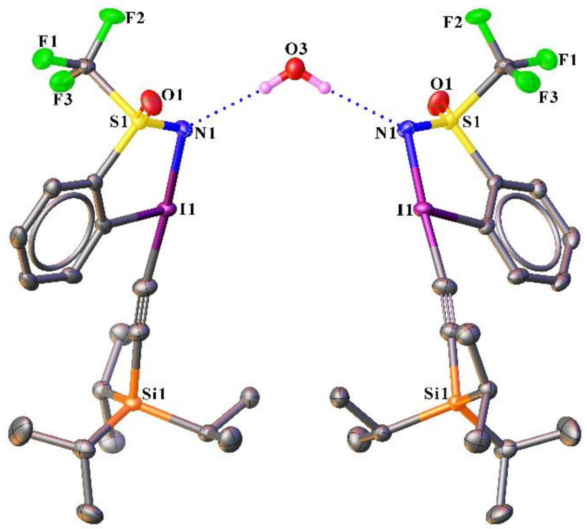

Compound	TIPS-CF3-EBS
Identification code	CCDC 2072276
Formula	$C_{72}H_{102}F_{12}I_4N_4O_5S_4Si_4$
$D_{calc.}$/ g cm^{-3}	1.610
μ/mm^{-1}	1.681
Formula Weight	2079.77
Colour	clear colourless
Shape	prism
Size/mm^3	0.79×0.39×0.29
T/K	140.00(10)
Crystal System	tetragonal
Flack Parameter	-0.003(5)
Space Group	$I4_1$
a/Å	19.07771(12)
b/Å	19.07771(12)
c/Å	23.5685(2)
α/°	90
β/°	90
γ/°	90
V/Å^3	8577.97(14)
Z	4
Z'	0.5
Wavelength/Å	0.71073
Radiation type	Mo $K\alpha$
Θ_{min}/°	2.539
Θ_{max}/°	32.974
Measured Refl's.	52957
Ind't Refl's	14808
Refl's with I > 2(I)	13940
R_{int}	0.0271
Parameters	490
Restraints	1
Largest Peak/e Å^{-3}	0.412
Deepest Hole/e Å^{-3}	-0.496
GooF	1.022
wR_2 (all data)	0.0478
wR_2	0.0465
R_1 (all data)	0.0263
R_1	0.0228

Table 7: Fractional Atomic Coordinates (×10^4) and Equivalent Isotropic Displacement Parameters (Å^2×10^3) for **6**. U_{eq} is defined as 1/3 of the trace of the orthogonalised U_{ij}.

Atom	x	y	z	U_{eq}
I1	3852.3(2)	6136.9(2)	4292.4(2)	18.24(4)
S1	2931.5(3)	5223.6(3)	5161.7(3)	17.47(12)
Si1	4120.7(4)	7403.1(4)	2380.8(3)	18.00(14)
F1	1828.5(10)	5661.6(11)	5773.2(8)	36.8(4)
F2	2831.1(10)	5712.8(11)	6194.8(7)	34.5(4)
F3	2568.8(11)	6464.7(10)	5550.2(8)	35.5(5)
O1	2699.8(12)	4536.8(11)	5340.6(10)	29.9(5)
N1	3675.1(12)	5429.4(13)	5085.2(10)	21.5(5)
C1	2789.2(13)	5845.6(13)	4129.6(11)	17.2(5)
C2	2452.2(13)	5481.5(13)	4554.4(11)	16.6(5)
C3	1744.6(14)	5296.6(15)	4494.8(12)	22.2(5)
C4	1389.7(15)	5501.7(17)	4009.4(13)	27.4(6)
C5	1743.0(17)	5857.4(16)	3583.1(13)	27.1(6)
C6	2450.8(16)	6033.7(14)	3635.0(12)	22.3(6)
C7	2507.0(15)	5797.3(15)	5701.1(12)	24.1(5)
C8	3871.4(17)	6686.7(16)	3524.0(12)	27.1(6)
C9	3953.8(16)	6981.6(16)	3081.0(12)	25.1(6)

Atom	x	y	z	U_{eq}
C10	4256.3(15)	8373.4(15)	2506.9(12)	21.9(5)
C11	4639.9(18)	8553.4(17)	3058.1(14)	31.1(7)
C12	4610.0(19)	8734.9(17)	2002.5(15)	33.3(7)
C13	3328.8(15)	7233.2(16)	1924.1(12)	25.8(6)
C14	2701.1(18)	7681(3)	2102.0(19)	53.8(12)
C15	3470.6(19)	7324(2)	1287.5(14)	38.0(8)
C16	4907.7(15)	6935.4(15)	2084.7(12)	22.7(5)
C17	5569.8(16)	7052.2(17)	2435.4(13)	28.6(6)
C18	4759.5(19)	6144.5(17)	2020.9(16)	35.0(7)
I2	3994.7(2)	3555.5(2)	5697.6(2)	17.93(4)
S2	4825.4(3)	2565.3(3)	4828.2(3)	16.80(12)
Si2	2772.3(4)	4065.3(4)	7590.8(3)	17.12(14)
F4	3553.4(9)	2352.3(11)	4432.4(8)	35.1(4)
F5	4333.5(11)	2561.9(10)	3796.2(7)	33.8(4)
F6	4273.4(11)	1541.9(10)	4182.9(8)	36.9(5)
O2	5482.4(10)	2260.0(11)	4648.7(10)	27.9(5)
N2	4700.0(12)	3325.5(12)	4908.5(10)	20.2(4)
C19	4179.0(13)	2472.0(13)	5854.8(11)	15.9(5)
C20	4520.3(13)	2102.4(14)	5433.2(11)	16.4(5)
C21	4658.1(15)	1389.3(15)	5500.9(13)	22.6(5)
C22	4431.2(16)	1060.9(15)	5991.9(13)	26.1(6)
C23	4091.2(15)	1437.8(16)	6415.6(13)	25.5(6)
C24	3963.7(14)	2150.3(15)	6350.3(11)	20.7(5)
C25	4210.3(15)	2225.9(15)	4279.4(13)	24.0(5)
C26	3450.1(15)	3638.6(16)	6463.8(12)	23.9(6)
C27	3183.4(15)	3809.8(15)	6906.0(12)	23.7(6)
C28	3270.4(15)	4840.1(16)	7878.3(12)	23.1(6)
C29	4051.7(18)	4660(2)	7962.6(16)	40.0(8)
C30	3195(2)	5507.0(18)	7528.1(15)	37.0(8)
C31	1818.6(14)	4245.8(14)	7439.3(12)	20.5(5)
C32	1444.6(17)	4601.0(18)	7942.7(13)	29.8(6)
C33	1679.8(17)	4651.6(17)	6886.8(13)	29.8(6)
C34	2876.9(16)	3293.3(15)	8081.3(12)	24.1(6)
C35	2803.1(19)	3484.1(18)	8712.4(13)	31.9(7)
C36	2365(2)	2705.5(18)	7936.1(18)	47.0(10)
O3	5000	5000	5677.3(15)	27.5(6)

Table 8: Anisotropic Displacement Parameters (×10^4) for **6**. The anisotropic displacement factor exponent takes the form: $-2\pi^2[h^2a^{*2} \times U_{11}+ ... +2hka^* \times b^* \times U_{12}]$

Atom	U_{11}	U_{22}	U_{33}	U_{23}	U_{13}	U_{12}
I1	20.68(8)	19.96(8)	14.06(7)	0.21(6)	0.56(6)	-4.30(6)
S1	18.4(3)	16.6(3)	17.4(3)	4.1(2)	-0.3(2)	2.1(2)
Si1	19.4(3)	19.7(3)	14.9(3)	2.5(3)	1.2(3)	-2.1(3)
F1	24.2(9)	59.7(13)	26.5(10)	0.6(9)	5.3(8)	6.4(8)
F2	36.6(11)	52.3(13)	14.5(8)	3.4(8)	-3.4(7)	1.1(9)
F3	54.9(13)	23.6(9)	28.1(10)	-1.7(7)	4.4(9)	10.9(9)
O1	31.6(11)	19.6(10)	38.5(12)	13.4(9)	-1.0(10)	1.3(8)
N1	18.5(10)	28.7(12)	17.3(10)	5.1(9)	-1.2(9)	1.1(9)
C1	19.3(12)	14.2(11)	18.1(12)	-4.2(9)	-4.8(9)	0.6(9)
C2	19.0(11)	14.0(11)	16.9(11)	-1.8(9)	-2.3(9)	1.7(9)
C3	18.8(12)	23.5(13)	24.3(13)	-4.4(11)	0.1(10)	-0.1(10)
C4	18.0(13)	32.5(15)	31.7(15)	-12.3(13)	-8.1(11)	3.6(11)
C5	32.1(15)	26.4(15)	22.6(14)	-6.1(11)	-12.0(12)	8.5(12)
C6	33.8(15)	17.3(12)	16.0(12)	-1.6(10)	-4.9(11)	2.9(11)
C7	25.7(13)	30.7(14)	15.9(11)	3.7(12)	0.2(11)	2.8(11)
C8	34.7(16)	25.3(14)	21.2(13)	1.6(11)	4.5(12)	-5.4(12)
C9	28.0(14)	25.4(14)	21.8(14)	0.5(11)	2.2(11)	-2.5(11)
C10	22.5(13)	21.1(12)	22.0(13)	1.8(10)	0.5(10)	-0.6(10)
C11	36.4(17)	26.9(15)	30.0(16)	-3.1(12)	-4.0(13)	-2.3(13)
C12	41.9(19)	24.1(14)	33.9(16)	7.1(13)	3.4(14)	-5.2(13)
C13	23.1(13)	30.7(15)	23.6(14)	1.4(11)	-3.3(11)	-8.5(11)
C14	19.5(16)	90(3)	52(2)	-21(2)	-3.0(16)	2.1(18)

Atom	U_{11}	U_{22}	U_{33}	U_{23}	U_{13}	U_{12}
C15	37.6(19)	52(2)	24.5(15)	1.7(15)	-10.3(14)	-2.7(16)
C16	25.4(13)	25.3(13)	17.3(12)	0.2(11)	1.3(11)	2.5(11)
C17	25.3(14)	33.4(16)	27.1(15)	-1.0(13)	-0.3(12)	6.4(12)
C18	39.6(18)	25.4(15)	39.9(19)	-7.2(14)	-1.0(15)	2.5(13)
I2	21.17(8)	18.42(8)	14.21(7)	-0.63(6)	2.39(6)	3.74(6)
S2	15.7(3)	18.2(3)	16.5(3)	0.2(2)	4.6(2)	-0.7(2)
Si2	18.6(3)	18.7(3)	14.0(3)	-1.5(3)	2.0(3)	1.4(3)
F4	21.2(8)	56.9(13)	27.3(9)	-4.9(9)	-0.3(7)	-6.6(8)
F5	48.7(12)	38.5(11)	14.3(8)	1.9(7)	2.7(8)	-5.5(9)
F6	57.6(13)	25.3(9)	27.7(10)	-7.3(8)	2.9(9)	-12.1(8)
O2	19.7(9)	28.8(11)	35.1(12)	0.5(9)	12.1(9)	3.3(8)
N2	24.6(11)	19.1(11)	16.8(10)	0.6(9)	4.9(9)	-1.3(9)
C19	13.5(11)	18.6(11)	15.6(11)	0.8(9)	-2.4(9)	-1.2(9)
C20	15.1(11)	18.2(11)	15.9(11)	2.1(9)	-1.5(9)	-1.1(9)
C21	21.9(13)	19.2(12)	26.7(14)	0.6(11)	-2.8(11)	1.8(10)
C22	27.9(15)	19.8(13)	30.7(15)	7.7(12)	-5.1(12)	-2.6(11)
C23	23.8(14)	30.1(15)	22.6(13)	9.5(12)	-2.8(11)	-7.0(11)
C24	18.0(12)	27.1(14)	17.0(12)	3.4(10)	0.2(10)	-2.1(10)
C25	30.6(14)	25.5(13)	16.0(11)	-2.2(11)	2.6(12)	-5.3(11)
C26	23.7(13)	27.1(14)	21.0(13)	-3.2(11)	0.4(11)	3.7(11)
C27	25.2(13)	27.2(14)	18.5(13)	-1.8(11)	1.1(10)	3.5(11)
C28	23.9(14)	28.7(14)	16.6(12)	-2.4(11)	1.5(10)	-5.1(11)
C29	25.9(16)	54(2)	40.4(19)	-4.1(17)	-3.1(14)	-7.8(15)
C30	48(2)	30.7(17)	32.1(17)	0.6(14)	-2.4(15)	-16.8(15)
C31	20.3(12)	21.0(12)	20.2(12)	-0.6(10)	0.9(10)	1.2(10)
C32	25.8(15)	35.9(17)	27.5(15)	-2.6(13)	4.8(12)	4.7(12)
C33	30.3(15)	33.4(16)	25.8(15)	4.7(13)	-5.7(12)	4.7(12)
C34	28.7(14)	21.3(13)	22.3(14)	1.1(10)	0.0(11)	6.7(11)
C35	42.7(19)	32.9(17)	20.0(14)	7.2(12)	2.1(13)	3.3(14)
C36	73(3)	19.8(15)	48(2)	4.2(15)	-15(2)	-4.4(16)
O3	27.4(15)	25.4(14)	29.8(15)	0	0	5.0(12)

Table 9: Bond Lengths in Å for **6**.

Atom	Atom	Length/Å
I1	N1	2.330(2)
I1	C1	2.138(3)
I1	C8	2.093(3)
S1	O1	1.446(2)
S1	N1	1.483(2)
S1	C2	1.768(3)
S1	C7	1.863(3)
Si1	C9	1.863(3)
Si1	C10	1.893(3)
Si1	C13	1.883(3)
Si1	C16	1.881(3)
F1	C7	1.331(3)
F2	C7	1.327(3)
F3	C7	1.327(3)
C1	C2	1.378(4)
C1	C6	1.380(4)
C2	C3	1.402(4)
C3	C4	1.386(4)
C4	C5	1.387(5)
C5	C6	1.397(4)
C8	C9	1.196(4)
C10	C11	1.530(4)
C10	C12	1.531(4)
C13	C14	1.530(5)
C13	C15	1.534(4)
C16	C17	1.526(4)
C16	C18	1.543(4)

Atom	Atom	Length/Å
I2	N2	2.337(2)
I2	C19	2.129(3)
I2	C26	2.090(3)
S2	O2	1.445(2)
S2	N2	1.482(2)
S2	C20	1.775(3)
S2	C25	1.863(3)
Si2	C27	1.859(3)
Si2	C28	1.883(3)
Si2	C31	1.886(3)
Si2	C34	1.883(3)
F4	C25	1.326(3)
F5	C25	1.328(3)
F6	C25	1.330(3)
C19	C20	1.382(4)
C19	C24	1.382(4)
C20	C21	1.395(4)
C21	C22	1.385(4)
C22	C23	1.391(5)
C23	C24	1.389(4)
C26	C27	1.205(4)
C28	C29	1.542(4)
C28	C30	1.523(4)
C31	C32	1.541(4)
C31	C33	1.538(4)
C34	C35	1.538(4)
C34	C36	1.526(5)

Table 10: Bond Angles in ° for **6**.

Atom	Atom	Atom	Angle/°
C1	I1	N1	81.70(9)
C8	I1	N1	170.68(10)
C8	I1	C1	89.51(11)
O1	S1	N1	124.61(13)
O1	S1	C2	109.28(13)
O1	S1	C7	101.57(13)
N1	S1	C2	108.82(13)
N1	S1	C7	110.07(14)
C2	S1	C7	99.45(12)
C9	Si1	C10	107.84(13)
C9	Si1	C13	107.15(13)
C9	Si1	C16	105.09(13)
C13	Si1	C10	111.58(13)
C16	Si1	C10	114.40(13)
C16	Si1	C13	110.29(13)
S1	N1	I1	112.93(12)
C2	C1	I1	116.33(18)
C2	C1	C6	121.8(3)
C6	C1	I1	121.9(2)
C1	C2	S1	119.1(2)
C1	C2	C3	120.2(2)
C3	C2	S1	120.6(2)
C4	C3	C2	118.8(3)
C3	C4	C5	119.9(3)
C4	C5	C6	121.6(3)
C1	C6	C5	117.6(3)
F1	C7	S1	113.3(2)
F2	C7	S1	108.93(19)
F2	C7	F1	108.5(2)
F3	C7	S1	110.0(2)
F3	C7	F1	107.9(2)
F3	C7	F2	108.1(2)
C9	C8	I1	173.2(3)
C8	C9	Si1	176.7(3)
C11	C10	Si1	114.7(2)
C11	C10	C12	110.3(3)
C12	C10	Si1	112.3(2)
C14	C13	Si1	112.0(2)
C14	C13	C15	110.1(3)
C15	C13	Si1	113.5(2)
C17	C16	Si1	113.0(2)
C17	C16	C18	110.3(2)
C18	C16	Si1	110.7(2)

Atom	Atom	Atom	Angle/°
C19	I2	N2	82.02(9)
C26	I2	N2	171.23(10)
C26	I2	C19	90.31(11)
O2	S2	N2	124.87(13)
O2	S2	C20	108.60(13)
O2	S2	C25	101.71(14)
N2	S2	C20	109.36(13)
N2	S2	C25	109.09(14)
C20	S2	C25	100.27(12)
C27	Si2	C28	107.77(13)
C27	Si2	C31	106.88(13)
C27	Si2	C34	106.44(13)
C28	Si2	C31	114.31(13)
C34	Si2	C28	109.86(13)
C34	Si2	C31	111.18(13)
S2	N2	I2	112.22(12)
C20	C19	I2	116.63(18)
C20	C19	C24	121.4(2)
C24	C19	I2	121.9(2)
C19	C20	S2	118.6(2)
C19	C20	C21	120.3(2)
C21	C20	S2	121.0(2)
C22	C21	C20	118.5(3)
C21	C22	C23	120.8(3)
C24	C23	C22	120.5(3)
C19	C24	C23	118.4(3)
F4	C25	S2	110.1(2)
F4	C25	F5	108.2(3)
F4	C25	F6	108.1(2)
F5	C25	S2	108.44(19)
F5	C25	F6	108.1(2)
F6	C25	S2	113.7(2)
C27	C26	I2	168.0(3)
C26	C27	Si2	179.5(3)
C29	C28	Si2	111.0(2)
C30	C28	Si2	114.4(2)
C30	C28	C29	110.3(3)
C32	C31	Si2	112.4(2)
C33	C31	Si2	114.7(2)
C33	C31	C32	110.5(2)
C35	C34	Si2	113.5(2)
C36	C34	Si2	111.7(2)
C36	C34	C35	109.4(3)

Table 11: Torsion Angles in ° for **6**.

Atom	Atom	Atom	Atom	Angle/°
I1	C1	C2	S1	-5.2(3)
I1	C1	C2	C3	177.14(19)
I1	C1	C6	C5	-176.0(2)
S1	C2	C3	C4	-179.2(2)
O1	S1	N1	I1	140.17(14)
O1	S1	C2	C1	-142.1(2)
O1	S1	C2	C3	35.5(3)
O1	S1	C7	F1	-49.0(2)
O1	S1	C7	F2	71.9(2)

Atom	Atom	Atom	Atom	Angle/˚
O1	S1	C7	F3	-169.9(2)
N1	S1	C2	C1	-3.1(2)
N1	S1	C2	C3	174.6(2)
N1	S1	C7	F1	177.2(2)
N1	S1	C7	F2	-61.9(2)
N1	S1	C7	F3	56.4(2)
C1	C2	C3	C4	-1.6(4)
C2	S1	N1	I1	8.94(17)
C2	S1	C7	F1	63.1(2)
C2	S1	C7	F2	-176.1(2)
C2	S1	C7	F3	-57.8(2)
C2	C1	C6	C5	1.6(4)
C2	C3	C4	C5	2.7(4)
C3	C4	C5	C6	-1.7(4)
C4	C5	C6	C1	-0.4(4)
C6	C1	C2	S1	177.1(2)
C6	C1	C2	C3	-0.6(4)
C7	S1	N1	I1	-99.07(14)
C7	S1	C2	C1	112.0(2)
C7	S1	C2	C3	-70.3(2)
C9	Si1	C10	C11	35.8(3)
C9	Si1	C10	C12	162.8(2)
C9	Si1	C13	C14	73.9(3)
C9	Si1	C13	C15	-160.7(2)
C9	Si1	C16	C17	-64.9(2)
C9	Si1	C16	C18	59.4(2)
C10	Si1	C13	C14	-43.9(3)
C10	Si1	C13	C15	81.5(3)
C10	Si1	C16	C17	53.2(2)
C10	Si1	C16	C18	177.5(2)
C13	Si1	C10	C11	153.2(2)
C13	Si1	C10	C12	-79.8(2)
C13	Si1	C16	C17	179.9(2)
C13	Si1	C16	C18	-55.7(2)
C16	Si1	C10	C11	-80.7(2)
C16	Si1	C10	C12	46.3(3)
C16	Si1	C13	C14	-172.3(3)
C16	Si1	C13	C15	-46.9(3)
I2	C19	C20	S2	4.2(3)
I2	C19	C20	C21	-179.6(2)
I2	C19	C24	C23	178.7(2)
S2	C20	C21	C22	177.5(2)
O2	S2	N2	I2	-141.17(14)
O2	S2	C20	C19	143.9(2)
O2	S2	C20	C21	-32.2(3)
O2	S2	C25	F4	169.0(2)
O2	S2	C25	F5	-72.8(2)
O2	S2	C25	F6	47.5(2)
N2	S2	C20	C19	4.6(3)
N2	S2	C20	C21	-171.4(2)
N2	S2	C25	F4	-57.5(2)
N2	S2	C25	F5	60.8(2)
N2	S2	C25	F6	-178.9(2)
C19	C20	C21	C22	1.5(4)
C20	S2	N2	I2	-10.11(17)
C20	S2	C25	F4	57.3(2)
C20	S2	C25	F5	175.5(2)
C20	S2	C25	F6	-64.1(2)
C20	C19	C24	C23	-0.7(4)
C20	C21	C22	C23	-1.8(4)
C21	C22	C23	C24	1.0(4)
C22	C23	C24	C19	0.3(4)

Atom	Atom	Atom	Atom	Angle/°
C24	C19	C20	S2	-176.3(2)
C24	C19	C20	C21	-0.2(4)
C25	S2	N2	I2	98.64(14)
C25	S2	C20	C19	-109.9(2)
C25	S2	C20	C21	74.0(2)
C27	Si2	C28	C29	-58.6(2)
C27	Si2	C28	C30	67.1(3)
C27	Si2	C31	C32	-168.8(2)
C27	Si2	C31	C33	-41.4(2)
C27	Si2	C34	C35	160.2(2)
C27	Si2	C34	C36	-75.6(3)
C28	Si2	C31	C32	-49.7(2)
C28	Si2	C31	C33	77.7(2)
C28	Si2	C34	C35	43.8(3)
C28	Si2	C34	C36	168.0(2)
C31	Si2	C28	C29	-177.2(2)
C31	Si2	C28	C30	-51.6(3)
C31	Si2	C34	C35	-83.8(3)
C31	Si2	C34	C36	40.5(3)
C34	Si2	C28	C29	57.0(2)
C34	Si2	C28	C30	-177.3(2)
C34	Si2	C31	C32	75.4(2)
C34	Si2	C31	C33	-157.2(2)

Table 12: Hydrogen Fractional Atomic Coordinates (×10^4) and Equivalent Isotropic Displacement Parameters (Å^2×10^3) for **6.** U_{eq} is defined as 1/3 of the trace of the orthogonalised U_{ij}.

Atom	x	y	z	U_{eq}
H3	1512.74	5035.38	4782.1	27
H4	905.08	5398.86	3968.68	33
H5	1497.7	5984.06	3247.63	32
H6	2690.58	6274.5	3340.41	27
H10	3778.12	8584.14	2539.62	26
H11A	4403.52	8325.5	3377.88	47
H11B	4636.61	9062.38	3113.91	47
H11C	5125.27	8387.48	3035.5	47
H12A	5082.73	8543.98	1951.43	50
H12B	4639.85	9239.9	2074.79	50
H12C	4333.87	8652.28	1658.29	50
H13	3192.69	6732.49	1984.28	31
H14A	2616.2	7622.13	2509.3	81
H14B	2284.97	7533.61	1889.59	81
H14C	2800.1	8175.42	2021.12	81
H15A	3613.54	7807.98	1211.69	57
H15B	3043.35	7216.88	1073.49	57
H15C	3845.65	7003.47	1170.76	57
H16	4996.82	7127.84	1696.71	27
H17A	5489.22	6893.85	2825.57	43
H17B	5687.55	7552.2	2436.6	43
H17C	5957.95	6785.49	2269.32	43
H18A	5158.9	5917.6	1834.72	52
H18B	4337.13	6076.96	1790.65	52
H18C	4688.43	5936.47	2396.68	52
H21	4902.28	1134.38	5216.57	27
H22	4508.57	572.53	6039.75	31
H23	3945.08	1206.28	6752.29	31
H24	3733.84	2410.16	6639.51	25
H28	3073.32	4943.44	8262.42	28
H29A	4092.42	4223.27	8179.31	60
H29B	4282.44	5040.24	8170.45	60
H29C	4276.54	4601.38	7591.8	60

Atom	x	y	z	U_{eq}
H30A	3341.57	5414.61	7136.55	56
H30B	3491.52	5875.58	7690.71	56
H30C	2705.04	5659.76	7531.78	56
H31	1590.39	3777.67	7391.13	25
H32A	1488.47	4305.28	8280.83	45
H32B	947.62	4665.09	7851.2	45
H32C	1659.96	5058.47	8016.09	45
H33A	1860.34	5130.31	6923.73	45
H33B	1174.33	4667.06	6813.73	45
H33C	1916.28	4415.9	6570.75	45
H34	3361.16	3104.94	8025.12	29
H35A	3154.15	3838.09	8812.44	48
H35B	2875.17	3063.97	8944.41	48
H35C	2332.79	3671.78	8782.39	48
H36A	2427.48	2567.66	7538.74	70
H36B	1883.49	2869.96	7994.02	70
H36C	2454.71	2301.35	8182.25	70
H3A	5340(20)	4870(20)	5490(17)	49(13)

Table 13: Hydrogen Bond information for **6**.

D	H	A	d(D-H)/Å	d(H-A)/Å	d(D-A)/Å	D-H-A/deg
O3	H3A	N1[1]	0.82(4)	2.18(4)	3.001(3)	172(4)

––––

[1]1-x,1-y,+

6. DFT calculations and coordinates

Geometries of the hypervalent iodine reagents were first optimized at the M06/def2-SVP level in Gaussian09.[20] Electrostatic potential maps and corresponding surface values were obtained using GaussView 5.0.9 via mapping onto the isodensity surface at 0.001au.[21] Reported dipole moments were obtained from the M06/def2-SVP computations on the optimized structures.

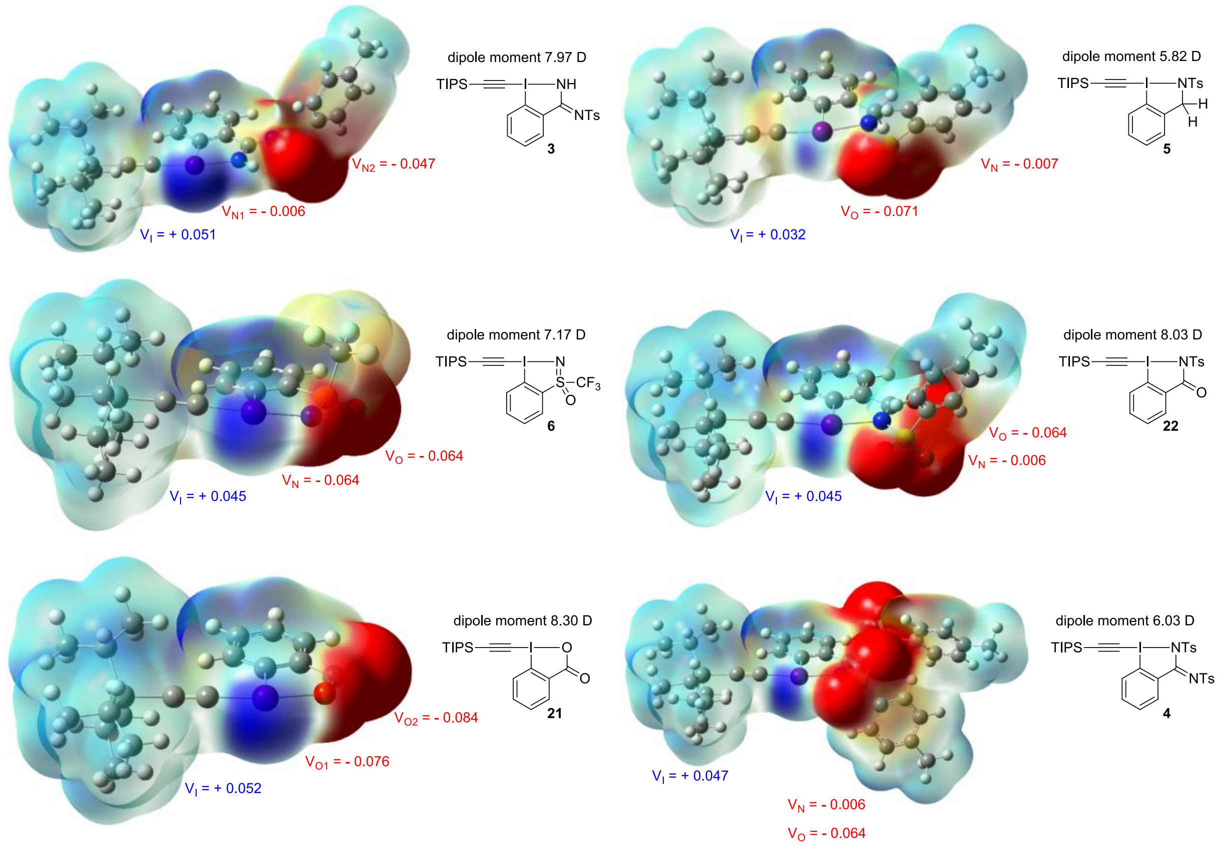

[20] (a) Zhao, Y.; Truhlar, D. G. The M06 Suite of Density Functionals for Main Group Thermochemistry, Thermochemical Kinetics, Noncovalent Interactions, Excited States, and Transition Elements: Two New Functionals and Systematic Testing of Four M06-Class Functionals and 12 Other Functionals. *Theor. Chem. Acc.* **2008**, *120*, 215–241. (b) Zhao, Y.; Truhlar, D. G. Density Functionals with Broad Applicability in Chemistry. *Acc. Chem. Res.* **2008**, *41*, 157–167. (c) Weigend, F.; Ahlrichs, R. Balanced Basis Sets of Split Valence, Triple Zeta Valence and Quadruple Zeta Valence Quality for H to Rn: Design and Assessment of Accuracy. *Phys. Chem. Chem. Phys.* **2005**, *7*, 3297–3305. (d) Frisch, M. J.; Trucks, G. W.; Schlegel, H. B.; Scuseria, G. E.; Robb, M. A.; Cheeseman, J. R.; Scalmani, G.; Barone, V.; Petersson, G. A.; Nakatsuji, H.; Li, X.; Caricato, M.; Marenich, A.; Bloino, J.; Janesko, B. G.; Gomperts, R.; Mennucci, B.; Hratchian, H. P.; Ortiz, J. V.; Izmaylov, A. F.; Sonnenberg, J. L.; Williams-Young, D.; Ding, F.; Lipparini, F.; Egidi, F.; Goings, J.; Peng, B.; Petrone, A.; Henderson, T.; Ranasinghe, D.; Zakrzewski, V. G.; Gao, J.; Rega, N.; Zheng, G.; Liang, W.; Hada, M.; Ehara, M.; Toyota, K.; Fukuda, R.; Hasegawa, J.; Ishida, M.; Nakajima, T.; Honda, Y.; Kitao, O.; Nakai, H.; Vreven, T.; Throssell, K.; Montgomery, Jr., J. A.; Peralta, J. E.; Ogliaro, F.; Bearpark, M.; Heyd, J. J.; Brothers, E.; Kudin, K. N.; Staroverov, V. N.; Keith, T.; Kobayashi, R.; Normand, J.; Raghavachari, K.; Rendell, A.; Burant, J. C.; Iyengar, S. S.; Tomasi, J.; Cossi, M.; Millam, J. M.; Klene, M.; Adamo, C.; Cammi, R.; Ochterski, J. W.; Martin, R. L.; Morokuma, K.; Farkas, O.; Foresman, J. B.; Fox, D. J. *Gaussian09, Revision D.01*; Gaussian, Inc.: Wallingford CT, 2016.

[21] Dennington, R.; Keith, T. A.; Millam, J. M. *GaussView 5.0.9*; Semichem Inc.: Shawnee Mission, KS, 2009.

Entry	Reagent	$V_{C,max}$ (au)
1	TIPS-H,Ts-EBZI **3**	+0.003
2	TIPS-Ts-EBz **5**	-0.001
3	TIPS-CF_3-EBS **6**	-0.002
4	TIPS-Ts-EBZ[a] **22**	-0.001
5	TIPS-EBX[a] **21**	+0.005
6	TIPS-Ts-EBZI **4**	+0.003

Table S1. Molecular electrostatic potential (MEP) maps computed at the M06/def2-SVP level. MEPs were mapped onto the 0.001 au isodensity surface. V_X represents the potential maximum around the atom X and is given in au.

Cartesian coordinate

TIPS-H,Ts-EBZI 3

I	-0.472028	-1.183486	0.031751
C	0.098391	0.762558	-0.619019
C	1.462638	0.911244	-0.851468
C	1.914538	2.160660	-1.283516
C	1.016931	3.205660	-1.467327
C	-0.823236	1.782111	-0.794340
C	-0.344247	3.018674	-1.224986
C	2.385894	-0.238980	-0.636831
H	2.987529	2.266548	-1.466993
H	1.379875	4.179835	-1.806089
H	-1.887984	1.620934	-0.600745
H	-1.049982	3.841210	-1.371072
C	-2.514316	-0.583112	0.154293
N	1.750199	-1.330190	-0.237310
C	-3.708085	-0.324438	0.283153
N	3.659153	-0.009656	-0.870700
Si	-5.512553	0.088600	0.468670
C	-5.657650	1.612144	1.610512
C	-6.184090	0.448374	-1.272124
C	-6.310955	-1.407716	1.329794
C	-7.829147	-1.280232	1.403605
C	-5.891622	-2.762028	0.769751
H	-5.907887	-1.333438	2.361748
H	-7.226641	0.797254	-1.122658
C	-5.394121	1.568666	-1.943165
C	-6.209741	-0.792367	-2.157459
C	-6.932045	2.413265	1.354867
H	-5.730974	1.169008	2.625551

C	-4.435000	2.524264	1.591101
H	-8.276063	-2.123869	1.958889
H	-8.154155	-0.354424	1.907667
H	-8.284105	-1.284641	0.396552
H	-6.272024	-3.584103	1.401654
H	-6.293294	-2.926950	-0.244376
H	-4.795994	-2.866638	0.709185
H	-6.536592	-0.542692	-3.182565
H	-5.205249	-1.246817	-2.239494
H	-6.893708	-1.568121	-1.776759
H	-5.808134	1.806390	-2.939085
H	-5.394651	2.503491	-1.357868
H	-4.340036	1.273039	-2.095596
H	-4.538866	3.340113	2.328320
H	-3.503646	1.980841	1.819189
H	-4.301613	3.002663	0.603466
H	-7.054774	3.213088	2.106404
H	-6.905569	2.906318	0.367186
H	-7.846511	1.797303	1.384768
H	2.356890	-2.126442	-0.014387
S	4.772785	-1.216925	-0.740016
O	5.251497	-1.574548	-2.069332
O	4.308726	-2.294302	0.155992
C	6.092075	-0.369208	0.099517
C	6.088839	-0.294056	1.490124
C	7.120558	0.380310	2.131170
C	8.158749	0.976809	1.402738
C	7.109375	0.209265	-0.650653
C	8.135428	0.881212	0.007497
H	5.284896	-0.773558	2.055768
H	7.128947	0.444891	3.224825
C	9.279343	1.672851	2.110200
H	7.088172	0.119127	-1.740055
H	8.941715	1.341714	-0.573590
H	9.836702	2.338486	1.434229
H	8.913092	2.272657	2.958470
H	10.001001	0.946081	2.521750

TIPS-Ts-EBZI 4

I	-1.266280	0.616041	-0.546623
C	-0.920032	-1.278108	0.387459
C	0.421181	-1.637651	0.595844
C	0.655051	-2.829222	1.294343
C	-0.401669	-3.631896	1.709422
C	-1.987695	-2.061331	0.791627
C	-1.716391	-3.260018	1.450130
C	1.518586	-0.746765	0.110697
H	1.678136	-3.124018	1.537719
H	-0.188320	-4.558668	2.247785
H	-3.018267	-1.747385	0.604009
H	-2.548181	-3.893995	1.770349
C	-3.351000	0.335677	-0.472820
N	1.016871	0.419205	-0.350900
C	-4.574694	0.253157	-0.424943
N	2.799232	-0.937988	0.118479
Si	-6.423540	0.115395	-0.237288
C	-6.948478	-1.642652	-0.766176
C	-6.784905	0.433130	1.601987
C	-7.188643	1.386163	-1.425094
C	-8.696033	1.504196	-1.220932
C	-6.513281	2.752652	-1.413117
H	-7.009212	0.931766	-2.422047
H	-7.869490	0.238960	1.733726
C	-6.008822	-0.541360	2.484124
C	-6.496754	1.872388	2.014042
C	-8.225720	-2.098140	-0.064875
H	-7.172734	-1.534857	-1.847534
C	-5.846780	-2.688037	-0.625025
H	-9.143035	2.214764	-1.938359
H	-9.218477	0.541390	-1.351681
H	-8.936734	1.875566	-0.208301
H	-6.913684	3.396412	-2.216045
H	-6.683849	3.285965	-0.462503

H	-5.422837	2.680457	-1.556962
H	-6.641515	2.010291	3.100211
H	-5.451303	2.152435	1.788431
H	-7.151189	2.596684	1.502857
H	-6.234666	-0.377971	3.552732
H	-6.236317	-1.597127	2.260672
H	-4.918724	-0.404721	2.361304
H	-6.187193	-3.673407	-0.989913
H	-4.940699	-2.418465	-1.191836
H	-5.547935	-2.825311	0.430466
H	-8.583369	-3.058680	-0.476072
H	-8.059291	-2.256914	1.015070
H	-9.053702	-1.375909	-0.161917
S	1.936548	1.557132	-1.171280
O	2.718412	0.958062	-2.239558
O	0.920168	2.567219	-1.486137
C	3.033325	2.224626	0.049829
C	2.517571	3.108003	0.996815
C	3.369471	3.629960	1.960423
C	4.728175	3.284683	1.986784
C	4.376565	1.863586	0.045512
C	5.215360	2.403347	1.015872
H	1.460022	3.385730	0.963479
H	2.979578	4.328846	2.708437
C	5.627437	3.837684	3.046942
H	4.749545	1.165765	-0.710384
H	6.276270	2.131166	1.022851
H	6.689000	3.703926	2.791605
H	5.445655	4.911501	3.212272
H	5.455596	3.332747	4.013144
S	3.672063	-2.318024	0.316283
O	3.829108	-2.654005	1.734493
O	3.196690	-3.349249	-0.603063
C	5.232864	-1.711809	-0.271782
C	5.383459	-1.456970	-1.634776
C	6.591436	-0.949515	-2.091024
C	7.654785	-0.701379	-1.208511

C	6.262967	-1.475946	0.628204
C	7.472523	-0.973424	0.150039
H	4.548397	-1.641928	-2.316248
H	6.721795	-0.739411	-3.158169
C	8.951403	-0.163722	-1.728217
H	6.108232	-1.688341	1.689421
H	8.296040	-0.789696	0.848590
H	9.682663	-0.008380	-0.921302
H	9.401759	-0.852275	-2.462497
H	8.807336	0.799088	-2.246092

TIPS-Ts-EBz 5

I	0.377718	-0.323221	-0.249244
C	0.302202	1.813452	-0.076826
C	1.488826	2.482861	-0.365529
C	1.470453	3.878497	-0.273270
C	0.315800	4.561158	0.090537
C	-0.865507	2.466816	0.295581
C	-0.852011	3.856557	0.379051
C	2.732792	1.744803	-0.773731
H	2.392462	4.427685	-0.494236
H	0.327470	5.652513	0.155502
H	-1.773170	1.899271	0.521904
H	-1.762181	4.385817	0.675341
C	-1.723247	-0.425673	0.064092
N	2.544929	0.350417	-0.490671
C	-2.935010	-0.555488	0.216271
Si	-4.783132	-0.663407	0.373893
C	-5.305304	0.213851	1.989269
C	-5.501695	0.208417	-1.156401
C	-5.222875	-2.509102	0.502671
C	-6.730160	-2.732672	0.436029
C	-4.476711	-3.407327	-0.476978
H	-4.879361	-2.772071	1.525147
H	-6.601063	0.224096	-1.006702
C	-5.004868	1.649215	-1.245102

C	-5.198458	-0.540418	-2.449166
C	-6.720798	0.781500	1.914373
H	-5.304357	-0.601411	2.742250
C	-4.321047	1.279567	2.461801
H	-6.982977	-3.802128	0.546298
H	-7.274531	-2.189136	1.226790
H	-7.143712	-2.402938	-0.534320
H	-4.669687	-4.473013	-0.260161
H	-4.793907	-3.231634	-1.518774
H	-3.386437	-3.250271	-0.437081
H	-5.551228	0.026641	-3.329033
H	-4.111384	-0.694247	-2.576919
H	-5.679559	-1.531112	-2.485368
H	-5.456542	2.174012	-2.105688
H	-5.237924	2.240893	-0.343449
H	-3.909312	1.680963	-1.387790
H	-4.633227	1.704468	3.432598
H	-3.300581	0.881336	2.583611
H	-4.261505	2.123753	1.750316
H	-7.040185	1.182967	2.892510
H	-6.783313	1.614662	1.192053
H	-7.472593	0.034134	1.610291
S	3.508162	-0.739981	-1.214802
O	3.959502	-0.293453	-2.532977
O	2.788623	-2.012866	-1.072954
C	4.955525	-0.816437	-0.176170
C	4.840801	-1.377834	1.096074
C	5.957848	-1.424708	1.916614
C	7.195136	-0.919866	1.486821
C	6.166893	-0.309072	-0.629080
C	7.280240	-0.362979	0.208830
H	3.876647	-1.777141	1.425159
H	5.879996	-1.866734	2.916307
C	8.389547	-0.990761	2.385862
H	6.227085	0.111341	-1.636796
H	8.240591	0.031044	-0.140869
H	9.272176	-0.519231	1.929104

H	8.197747	-0.489683	3.349198
H	8.650505	-2.036536	2.619964
H	2.931760	1.935563	-1.850783
H	3.596740	2.175716	-0.221712

TIPS-CF_3-EBS 6

I	1.009131	-1.287240	-0.419930
C	1.559970	0.803625	-0.428349
C	2.918121	1.053510	-0.480110
C	3.396120	2.359299	-0.551196
C	2.482504	3.409278	-0.537845
C	0.633884	1.835192	-0.420611
C	1.114899	3.144975	-0.469867
S	4.037915	-0.366607	-0.543353
H	4.474363	2.530026	-0.620567
H	2.837665	4.441839	-0.588606
H	-0.440070	1.629048	-0.382713
H	0.398290	3.971395	-0.464223
C	-1.018050	-0.675036	-0.110907
N	3.179760	-1.585379	-0.738421
C	-2.206636	-0.426533	0.075988
O	5.229170	-0.003052	-1.312174
Si	-4.001679	-0.006403	0.307333
C	-4.119199	1.449688	1.538140
C	-4.673577	0.467127	-1.407549
C	-4.824518	-1.538244	1.079485
C	-6.340727	-1.393215	1.158593
C	-4.423348	-2.865347	0.445715
H	-4.422155	-1.529067	2.114348
H	-5.716048	0.809383	-1.242587
C	-3.875578	1.623042	-2.005063
C	-4.696987	-0.720691	-2.362384
C	-5.382030	2.281024	1.328560
H	-4.196579	0.953251	2.527673
C	-2.880785	2.340148	1.564910
H	-6.802605	-2.266422	1.652619

H	-6.652609	-0.499886	1.725698
H	-6.791090	-1.319199	0.152190
H	-4.799370	-3.715442	1.042171
H	-4.842909	-2.978180	-0.568369
H	-3.329504	-2.973465	0.362293
H	-5.027243	-0.415606	-3.371300
H	-3.691480	-1.167191	-2.471016
H	-5.376875	-1.518308	-2.022398
H	-4.263970	1.903722	-3.000224
H	-3.901732	2.530030	-1.377695
H	-2.814156	1.344516	-2.139724
H	-2.976441	3.128763	2.332448
H	-1.963196	1.769225	1.782362
H	-2.727406	2.853266	0.597298
H	-5.491038	3.046118	2.117561
H	-5.353323	2.819284	0.365014
H	-6.304761	1.676706	1.333320
C	4.676354	-0.321359	1.225436
F	5.526198	-1.309357	1.398762
F	5.270982	0.825458	1.511879
F	3.644262	-0.480718	2.044956

TIPS-EBX 21

I	-1.845691	-1.392926	-0.171230
C	-2.513024	0.627011	0.014079
C	-3.894271	0.713958	0.071633
C	-4.456918	1.984585	0.202294
C	-3.638656	3.107353	0.267533
C	-1.660130	1.716711	0.074537
C	-2.250256	2.974755	0.202644
C	-4.758287	-0.530074	-0.008578
H	-5.548437	2.046343	0.247561
H	-4.083183	4.101266	0.369613
H	-0.573681	1.599321	0.023213
H	-1.610134	3.860419	0.251375
C	0.162605	-0.785442	-0.170353

O	-4.057795	-1.614608	-0.130683
C	1.348038	-0.468457	-0.177388
O	-5.963700	-0.442596	0.041881
Si	3.137210	0.051674	-0.146420
C	3.369318	1.450257	-1.426247
C	3.493568	0.649288	1.622099
C	4.144879	-1.464459	-0.692058
C	5.644542	-1.226521	-0.542707
C	3.718560	-2.778128	-0.046532
H	3.912531	-1.533798	-1.775375
H	4.519524	1.070527	1.594108
C	2.527990	1.760028	2.026328
C	3.460503	-0.483458	2.641618
C	4.520906	2.384444	-1.063093
H	3.647160	0.908696	-2.354228
C	2.100492	2.245616	-1.714260
H	6.225678	-2.095351	-0.898997
H	5.993172	-0.349468	-1.113760
H	5.924650	-1.065415	0.514115
H	4.246601	-3.631850	-0.506985
H	3.953351	-2.800868	1.030953
H	2.636479	-2.960976	-0.149908
H	3.596457	-0.097275	3.667390
H	2.489882	-1.012353	2.621171
H	4.249803	-1.232715	2.467654
H	2.757613	2.138503	3.038091
H	2.556154	2.623673	1.341182
H	1.486938	1.389722	2.050195
H	2.272988	2.994691	-2.507410
H	1.267438	1.601346	-2.038974
H	1.760811	2.802012	-0.821466
H	4.721573	3.101133	-1.878890
H	4.284735	2.983823	-0.166226
H	5.465394	1.853088	-0.858201

TIPS-Ts-EBZ 22

I	0.289822	-0.256924	-0.573182
C	0.306662	1.816342	-0.043805
C	1.553766	2.436711	-0.112302
C	1.629086	3.789750	0.222814
C	0.488375	4.486456	0.605456
C	-0.848627	2.484762	0.329433
C	-0.744487	3.836574	0.656962
C	2.793144	1.701222	-0.541110
H	2.614412	4.262287	0.165095
H	0.556617	5.546604	0.863679
H	-1.811993	1.967651	0.363939
H	-1.644461	4.383048	0.953872
C	-1.772948	-0.379240	-0.147029
N	2.477960	0.412103	-0.836615
C	-2.968902	-0.509560	0.097374
O	3.883116	2.236845	-0.577712
Si	-4.800017	-0.648906	0.413241
C	-5.202360	0.313443	2.013931
C	-5.658017	0.110891	-1.102950
C	-5.152890	-2.496367	0.689694
C	-6.648147	-2.788519	0.771598
C	-4.454756	-3.427267	-0.295513
H	-4.710760	-2.678348	1.691877
H	-6.739892	0.130861	-0.858838
C	-5.186262	1.544369	-1.328827
C	-5.459868	-0.726959	-2.361015
C	-6.643603	0.816780	2.041854
H	-5.090155	-0.450076	2.811330
C	-4.230485	1.448877	2.317552
H	-6.835114	-3.855284	0.987720
H	-7.153428	-2.206573	1.560841
H	-7.158065	-2.562687	-0.182530
H	-4.582393	-4.483006	0.002307
H	-4.868191	-3.330749	-1.313627
H	-3.372706	-3.228137	-0.361784
H	-5.918431	-0.239697	-3.239627
H	-4.386473	-0.861549	-2.588369

H	-5.906932	-1.730294	-2.271987
H	-5.684751	1.996164	-2.204671
H	-5.387542	2.200148	-0.465493
H	-4.098642	1.577439	-1.523099
H	-4.469175	1.927442	3.284104
H	-3.184217	1.105573	2.365058
H	-4.284290	2.241129	1.548770
H	-6.889045	1.260765	3.022786
H	-6.808908	1.604354	1.285659
H	-7.385250	0.023512	1.850032
S	3.549799	-0.699823	-1.457483
O	4.238302	-0.178152	-2.624231
O	2.714918	-1.902739	-1.562332
C	4.739409	-0.960511	-0.165479
C	4.504369	-1.962066	0.774904
C	5.432015	-2.162792	1.788975
C	6.590776	-1.379222	1.873024
C	5.878727	-0.162514	-0.109170
C	6.795071	-0.380239	0.914678
H	3.606217	-2.580181	0.693256
H	5.262201	-2.948910	2.532911
C	7.595776	-1.627437	2.953667
H	6.027268	0.622417	-0.854694
H	7.694529	0.241599	0.973199
H	8.292458	-0.783277	3.063409
H	7.109467	-1.801749	3.926675
H	8.199198	-2.524911	2.733440

7. NMR spectra

^{1}H-NMR (400 MHz, Chloroform-*d*) (8)

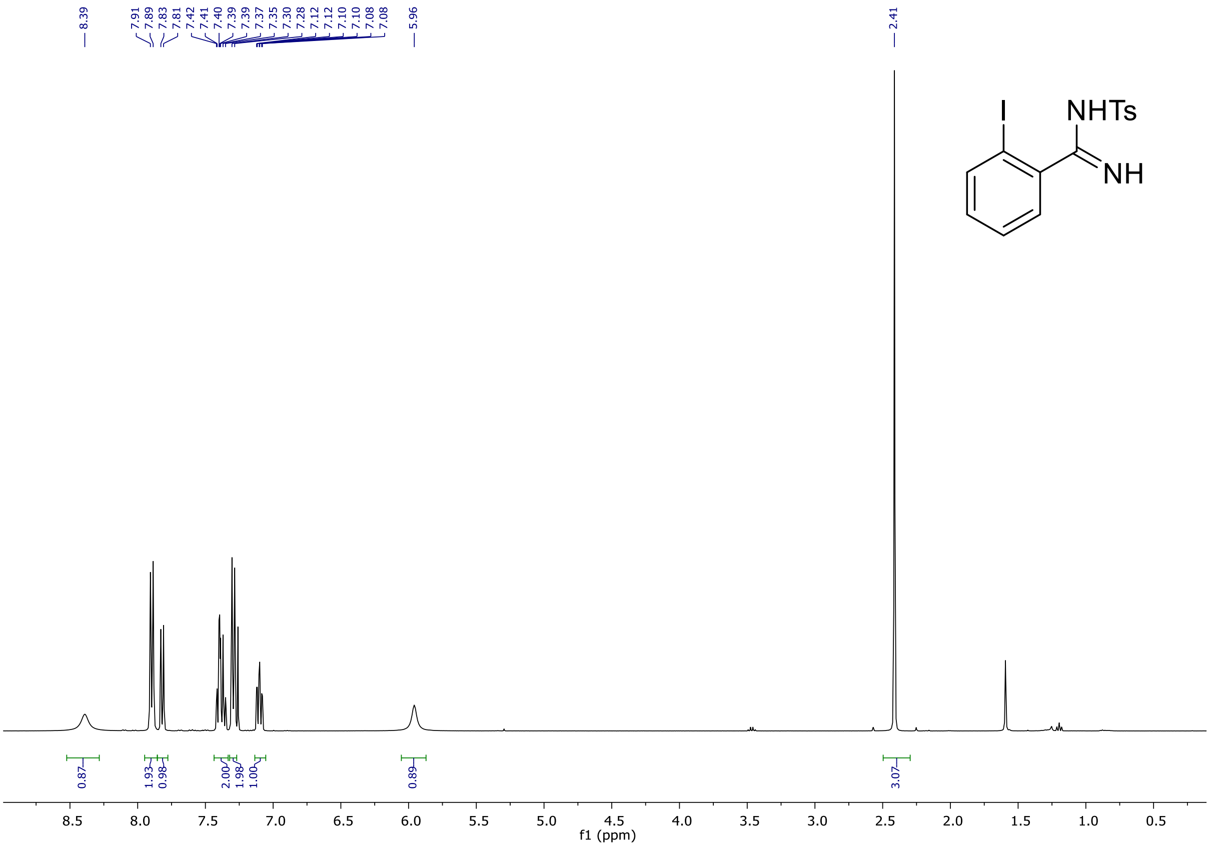

^{13}C-NMR (101 MHz, Chloroform-*d*) (8)

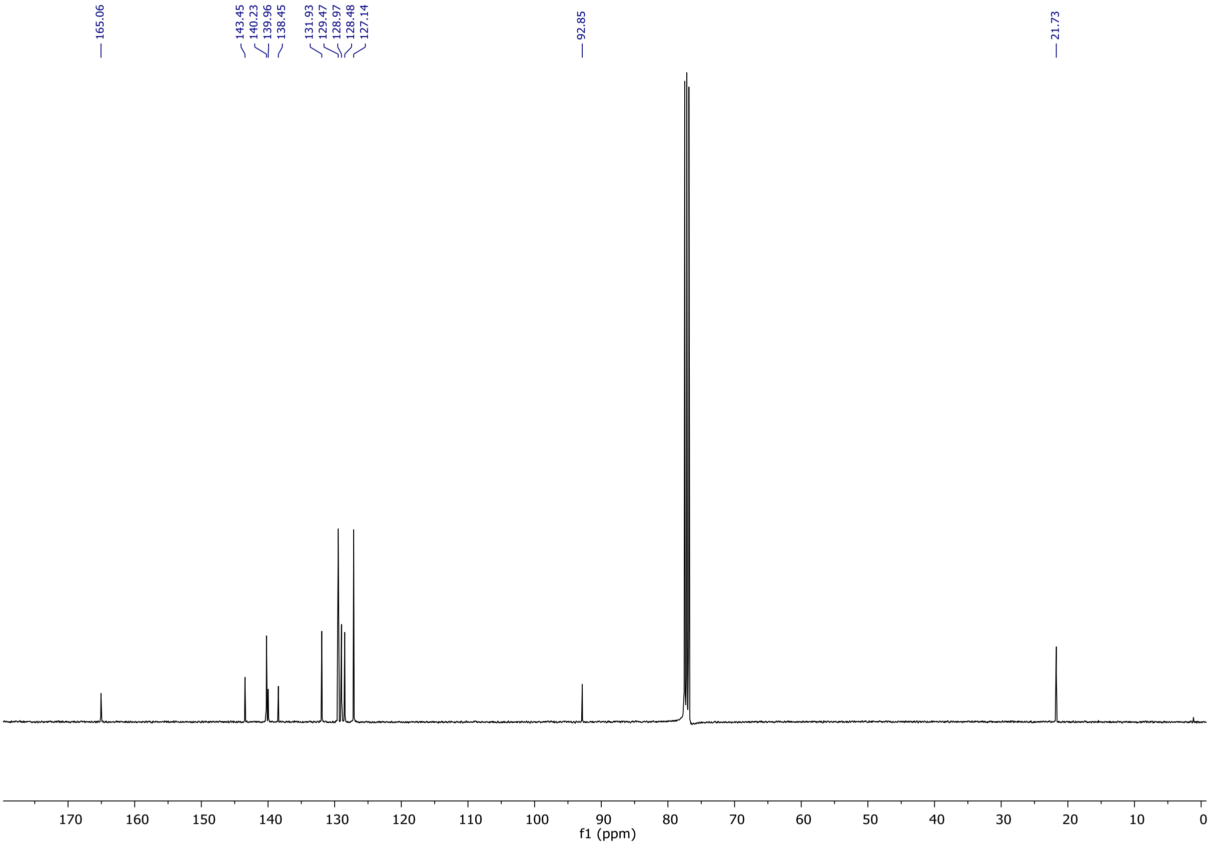

^{1}H-NMR (400 MHz, acetonitrile-d_3) (9)

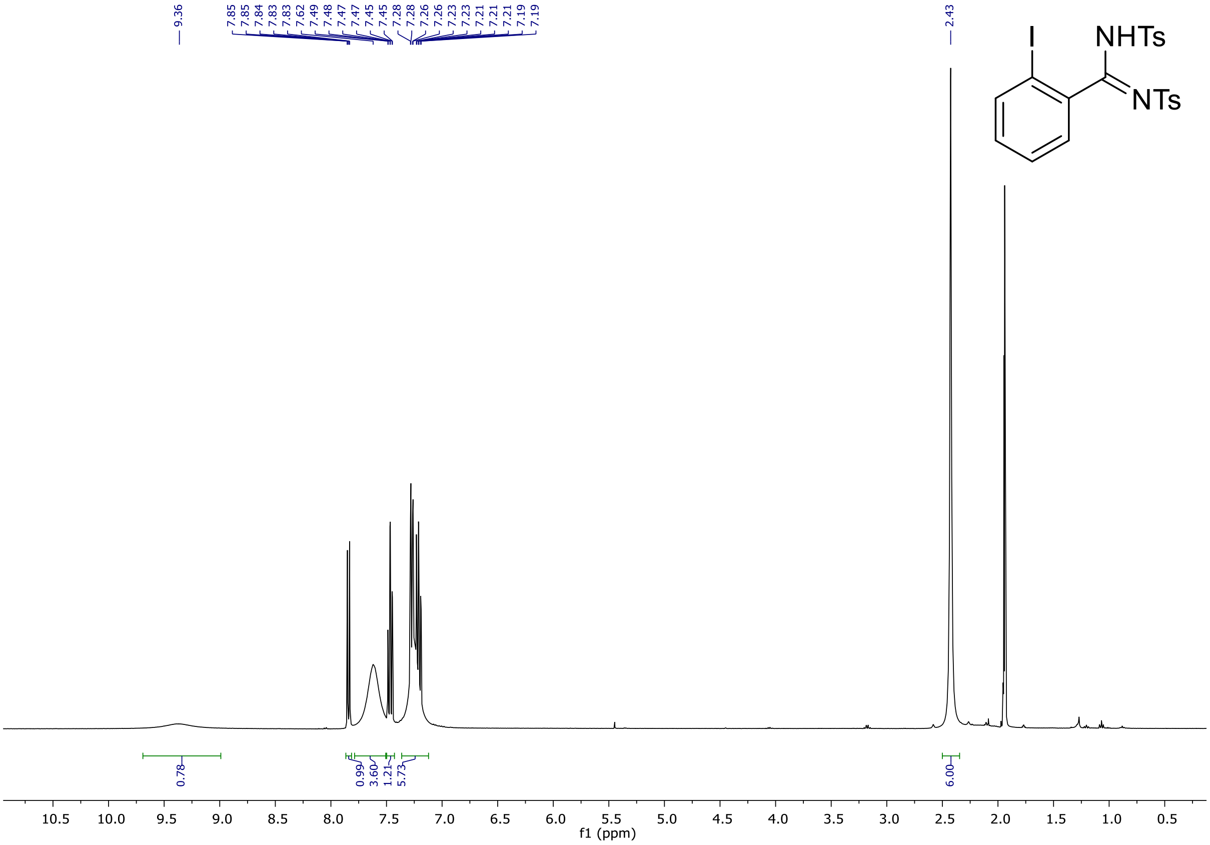

^{13}C-NMR (101 MHz, acetonitrile-d_3) (9)

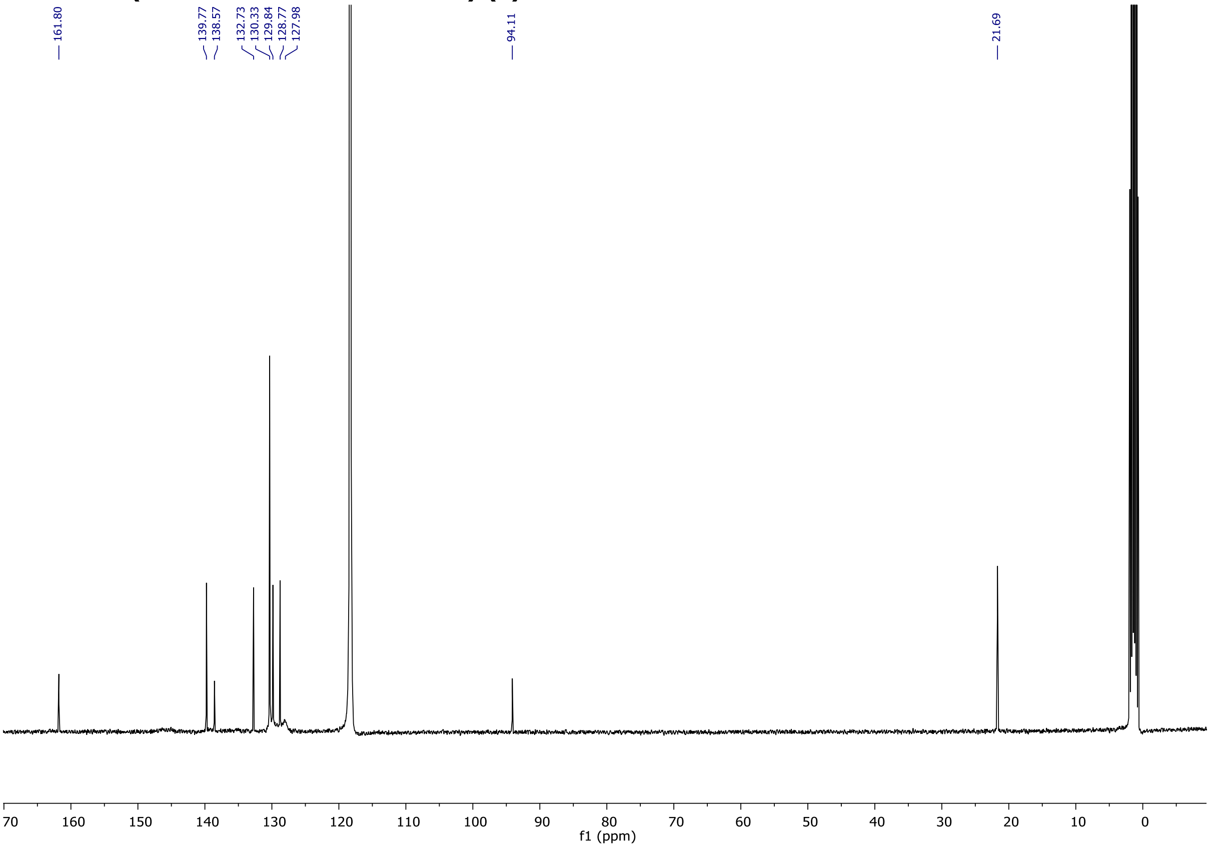

^{1}H-NMR (400 MHz, DMSO-d_6) (18)

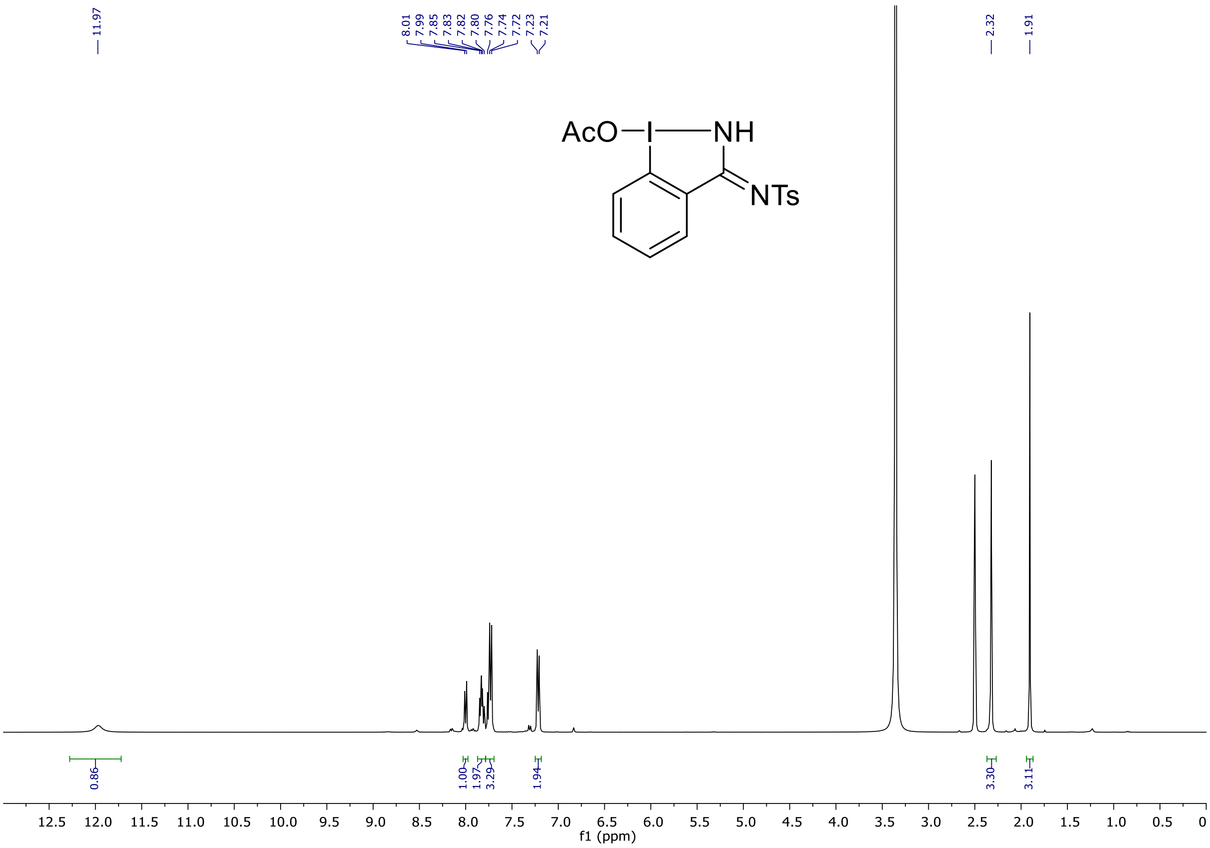

^{13}C-NMR (101 MHz, DMSO-d_6) (18)

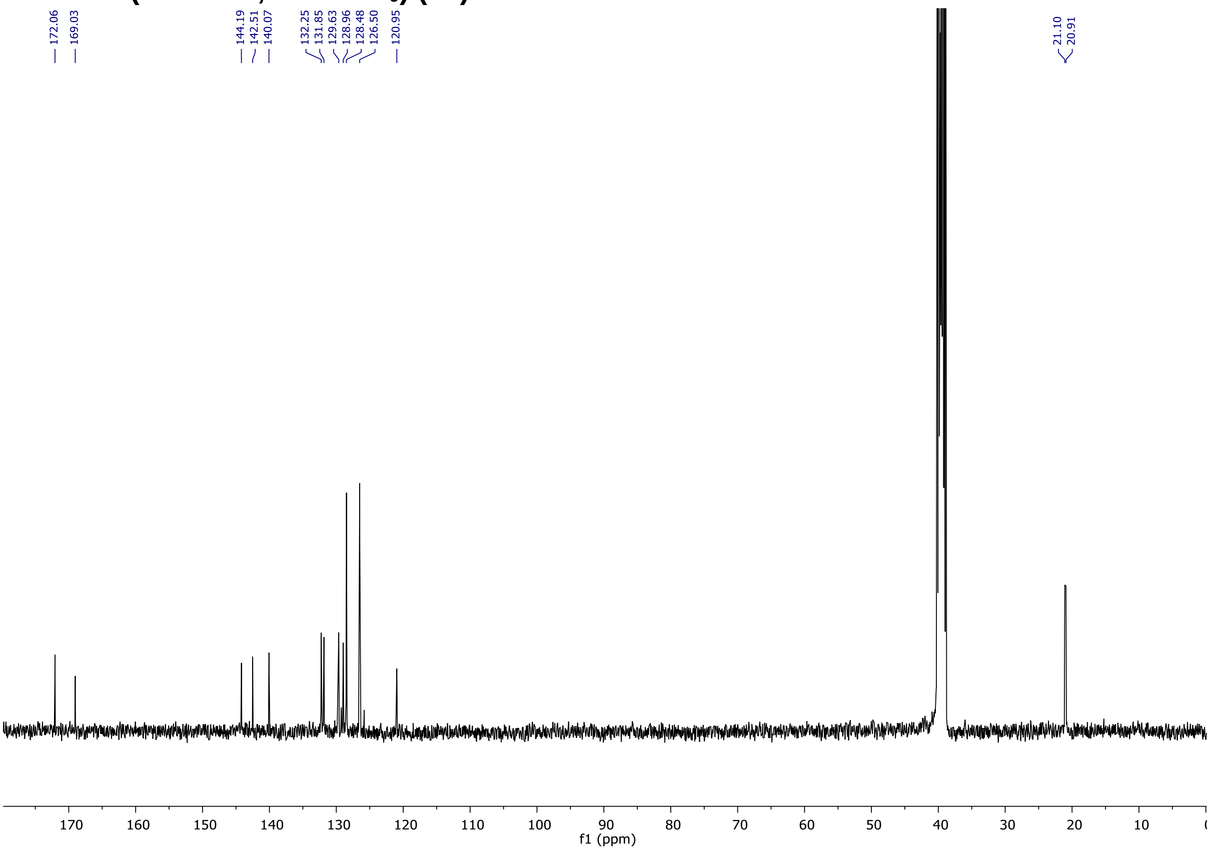

^{1}H-NMR (400 MHz, Chloroform-*d*) (3)

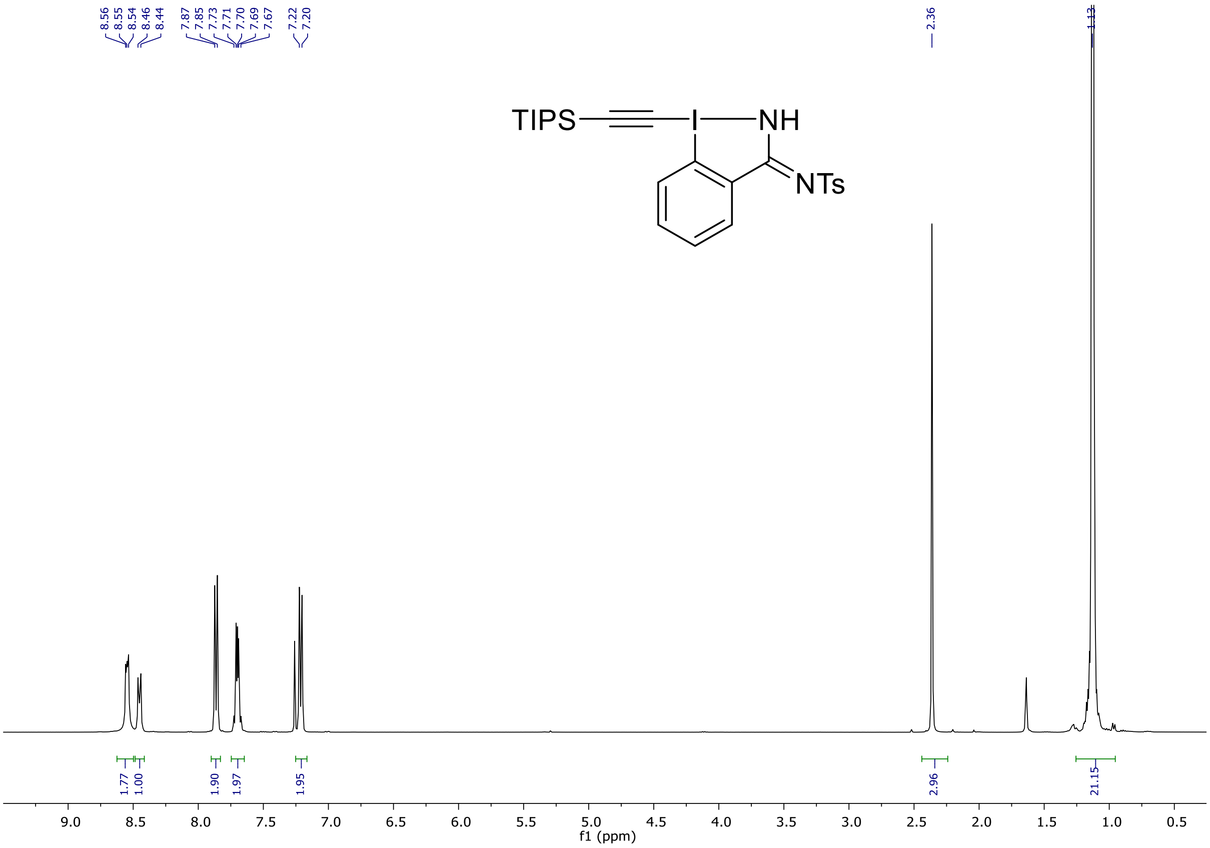

^{13}C-NMR (101 MHz, Chloroform-*d*) (3)

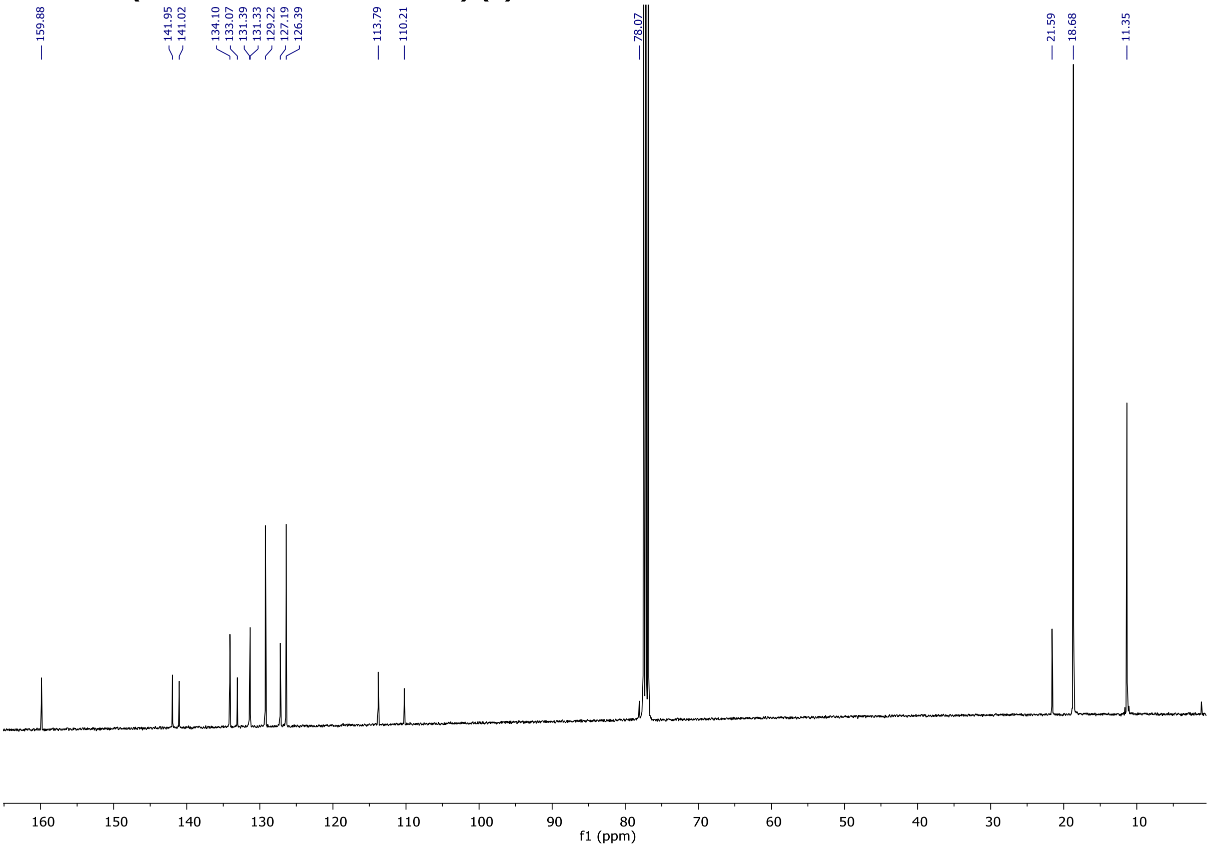

1H-NMR (400 MHz, chloroform-*d*) (19)

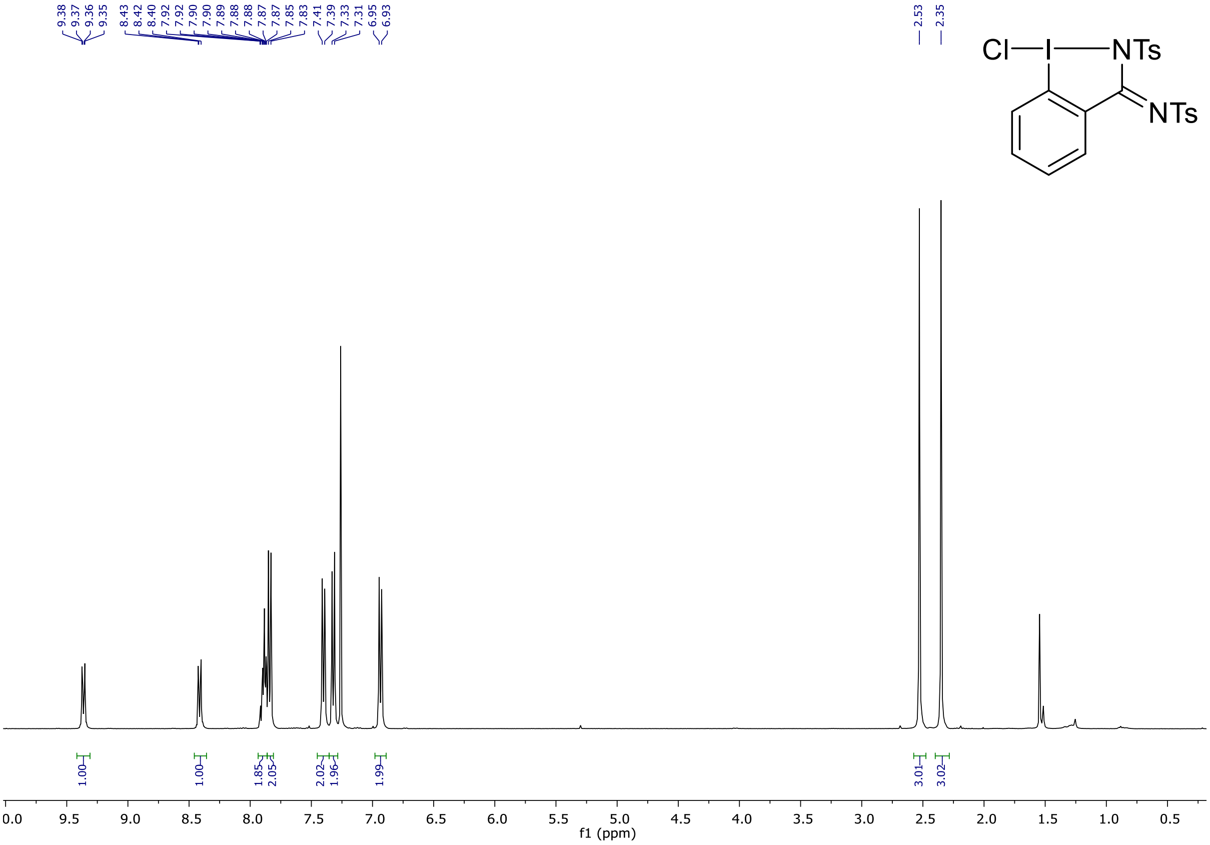

13C-NMR (101 MHz, chloroform-*d*) (19)

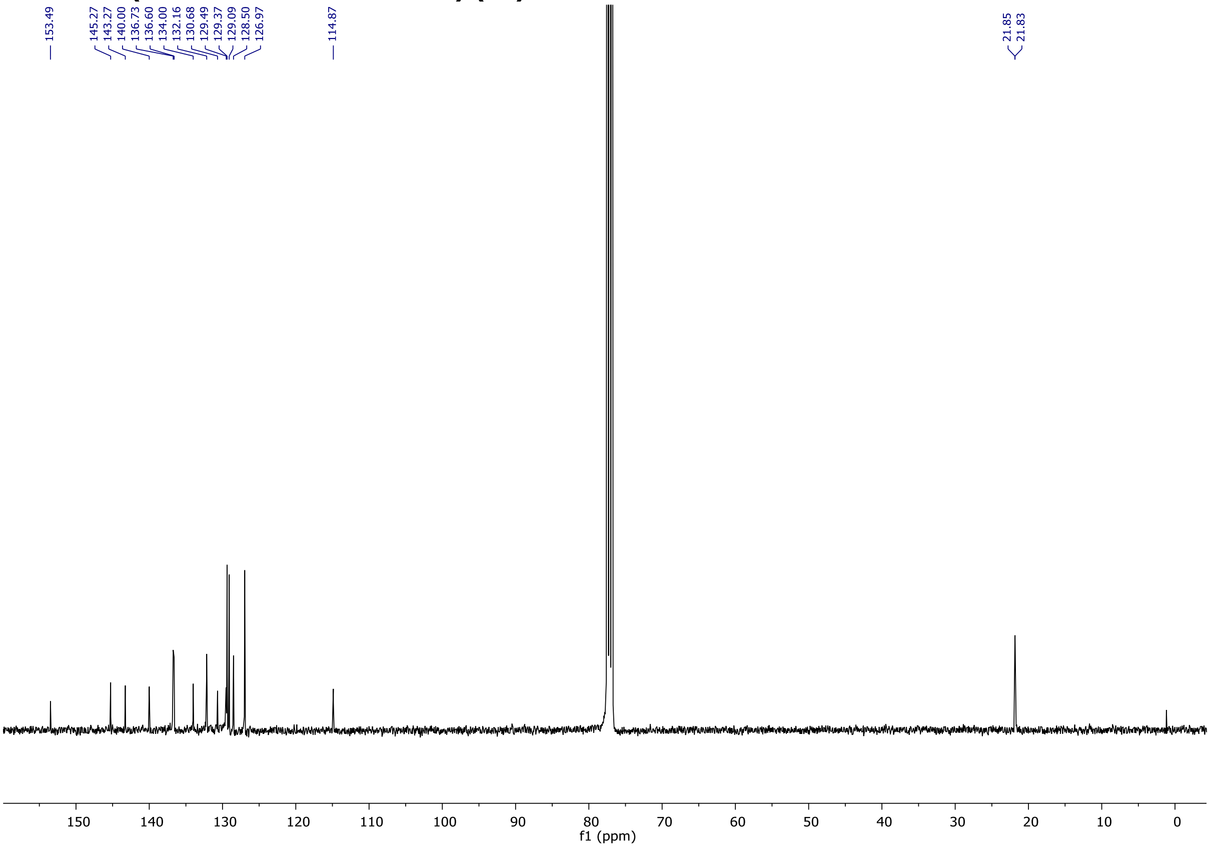

^{1}H-NMR (400 MHz, methylene chloride-d_2) (20)

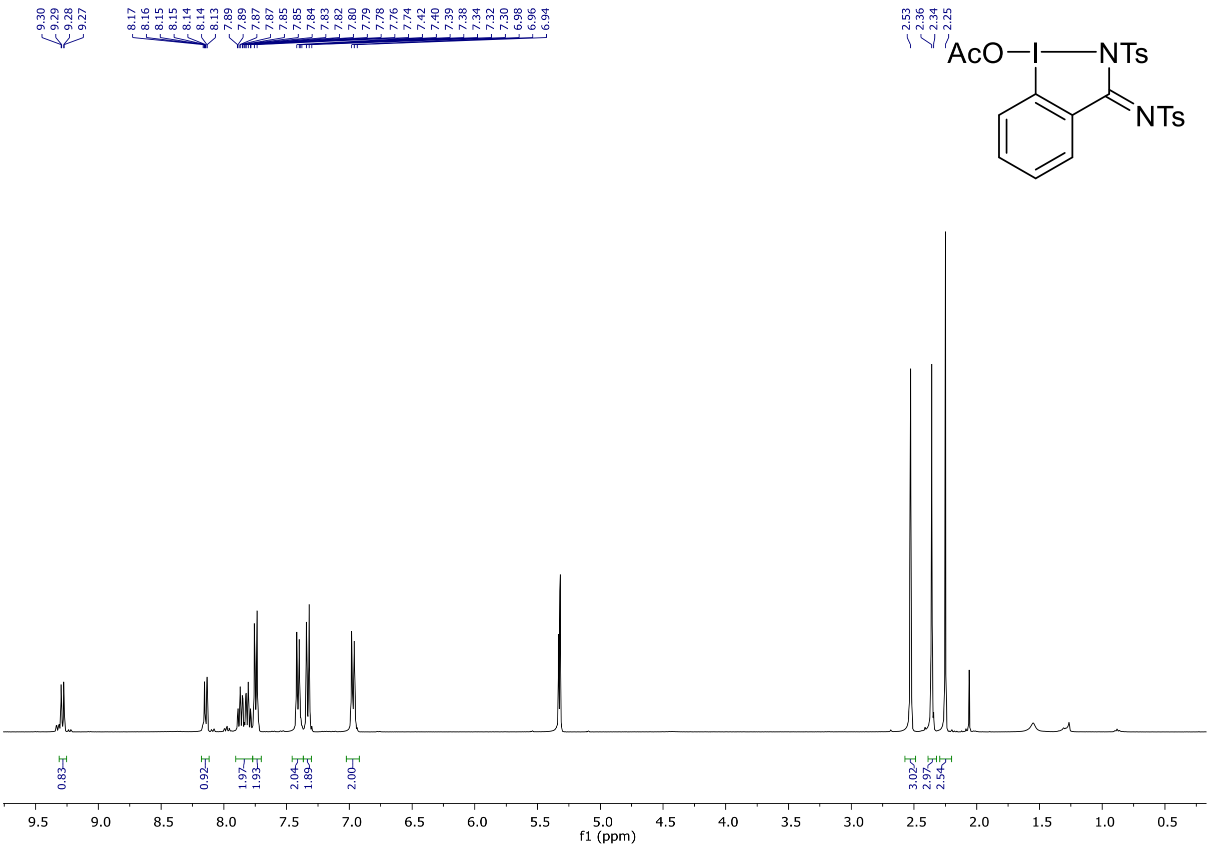

^{13}C-NMR (101 MHz, methylene chloride-d_2) (20)

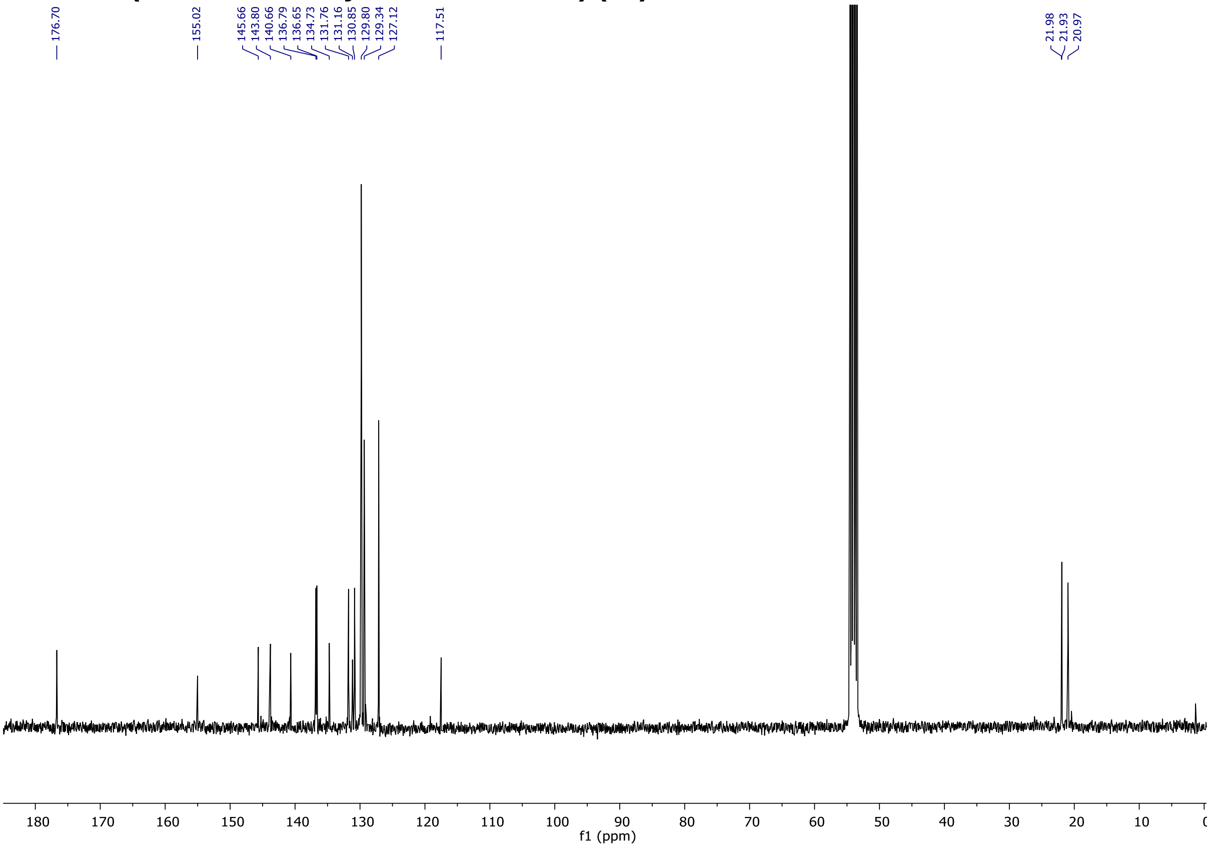

^{1}H-NMR (400 MHz, chloroform-*d*) (4)

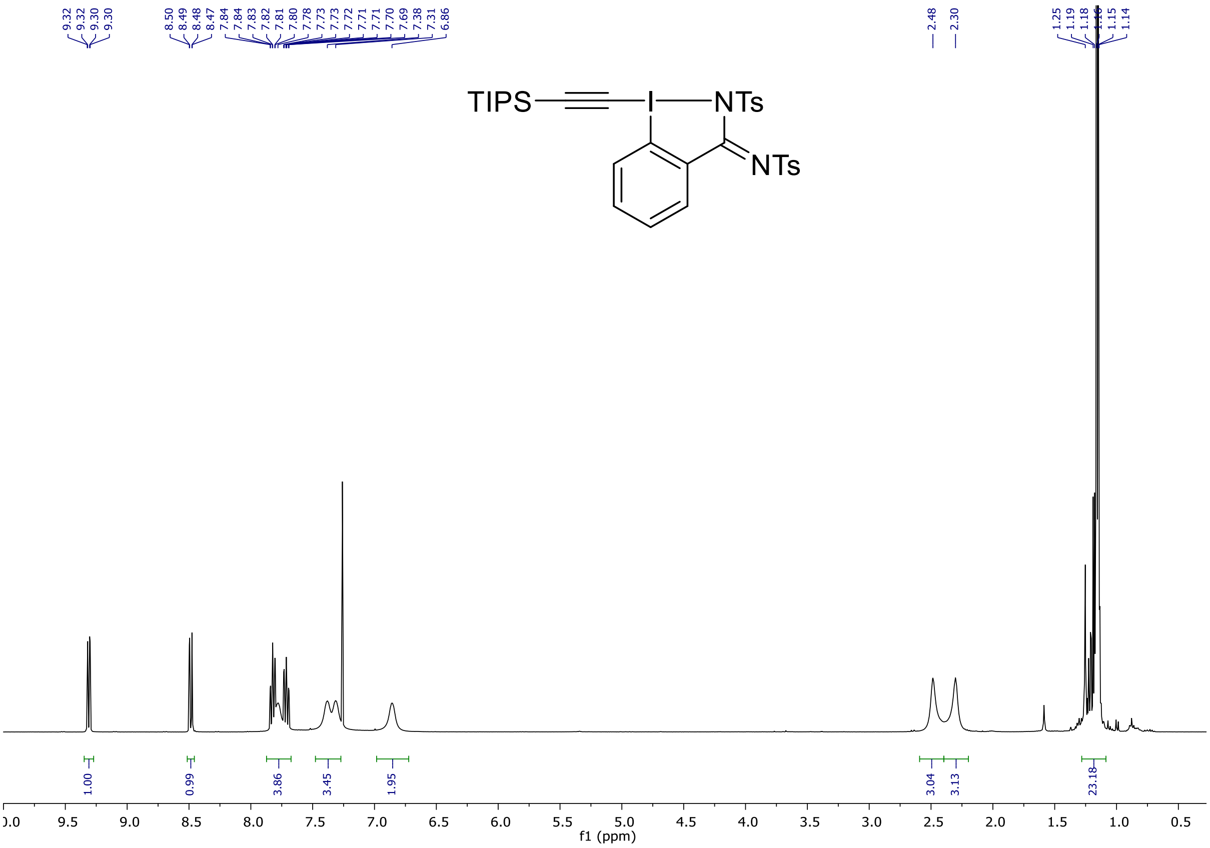

^{13}C-NMR (101 MHz, chloroform-*d*) (4)

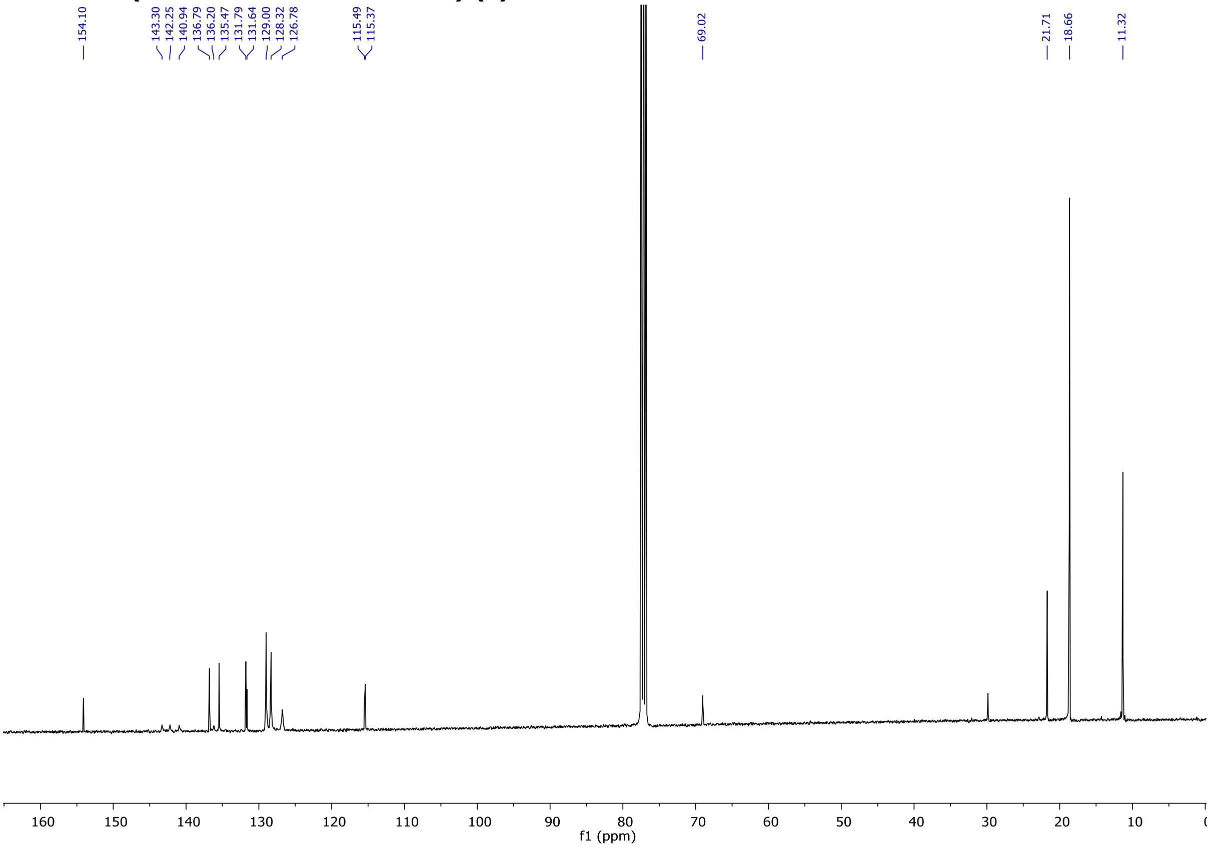

^{1}H-NMR (400 MHz, Chloroform-*d*) (5)

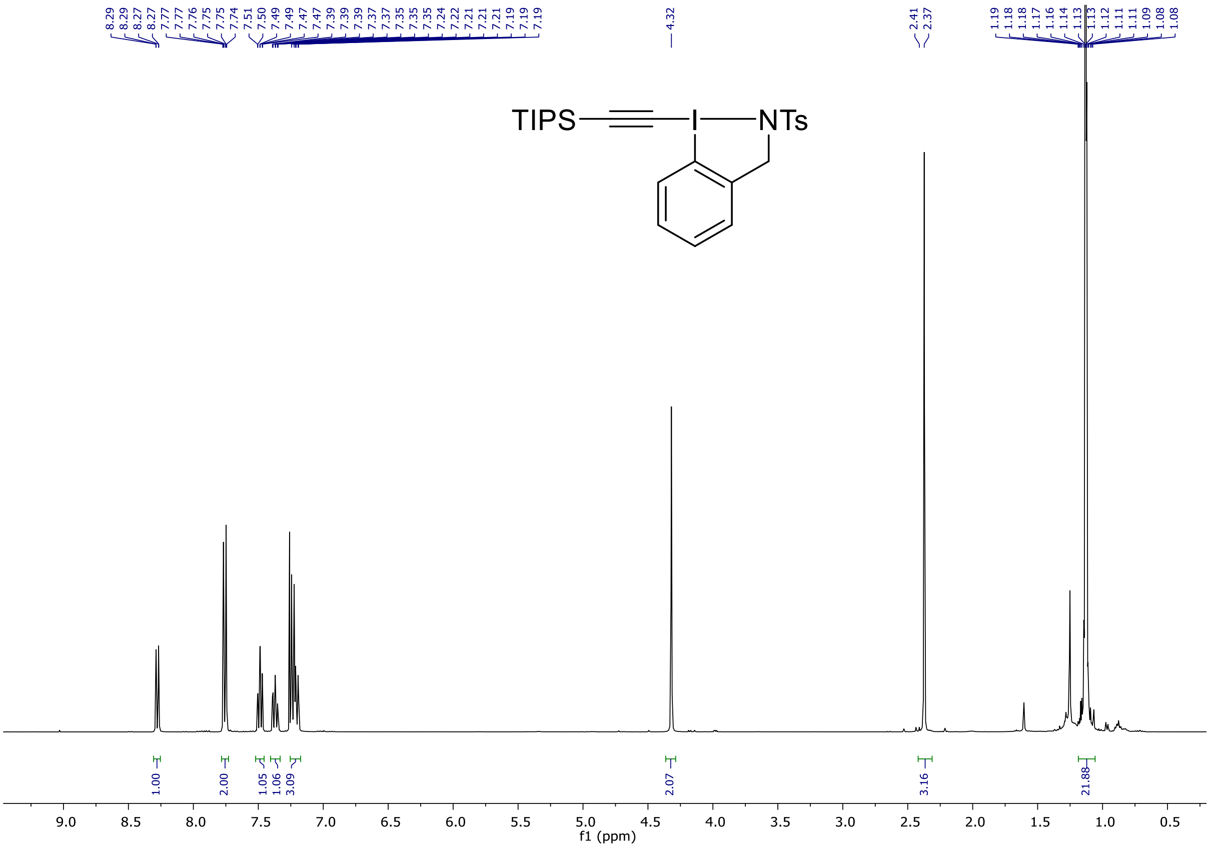

^{13}C-NMR (101 MHz, Chloroform-*d*) (5)

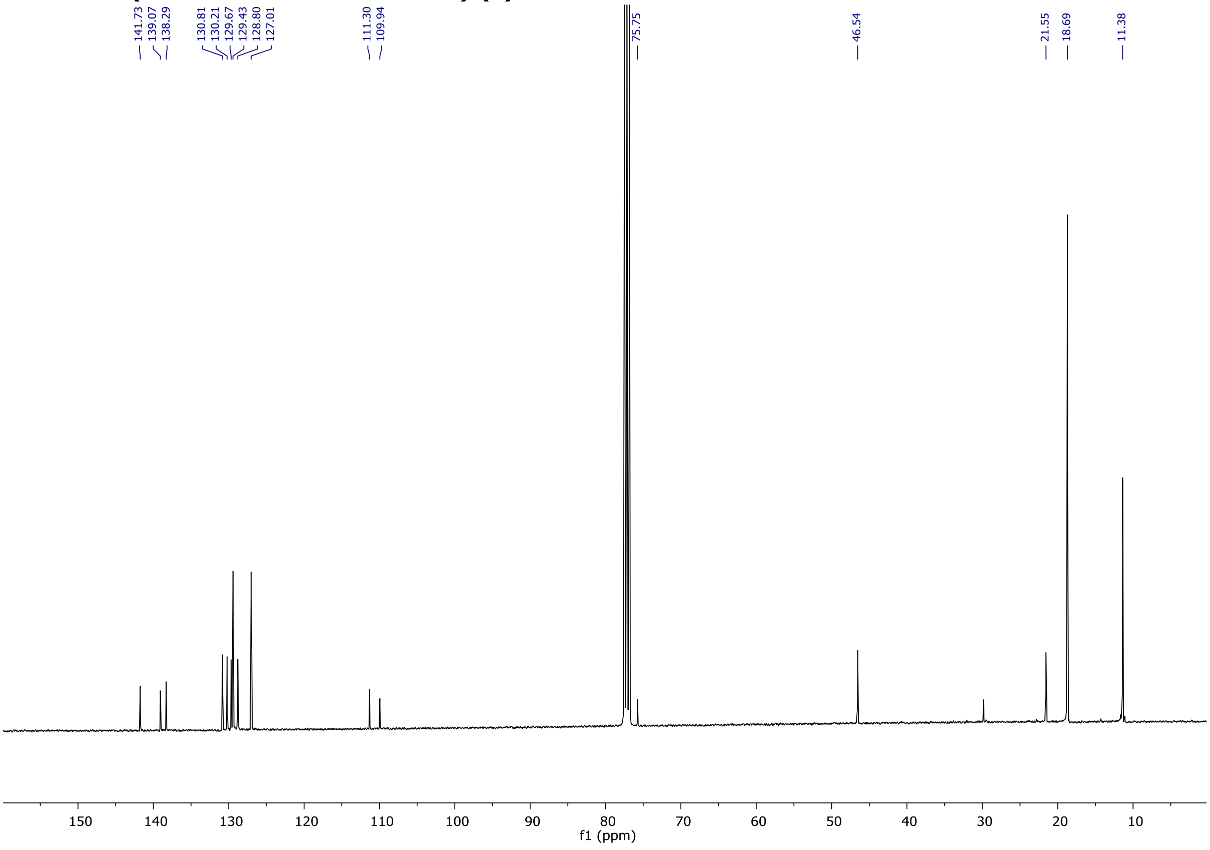

^{1}H-NMR (400 MHz, Chloroform-*d*) (6)

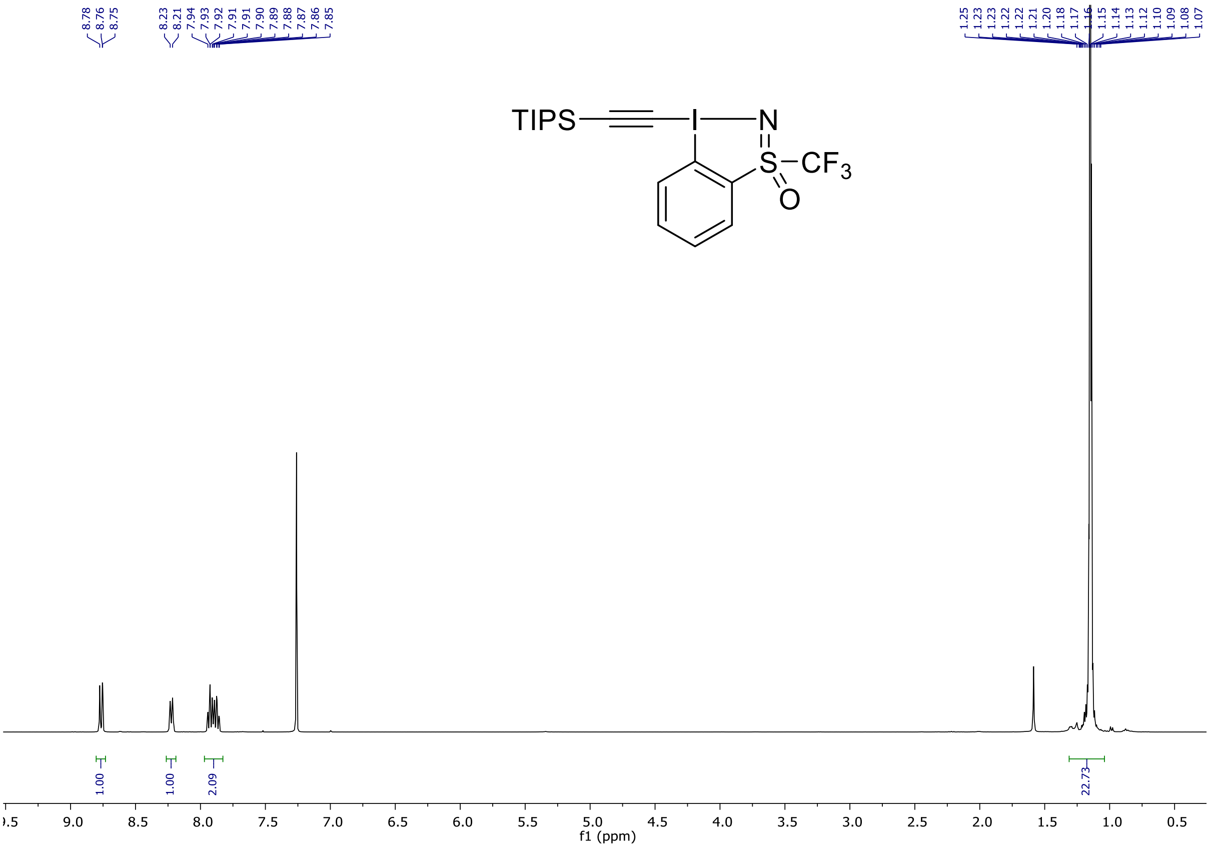

^{13}C-NMR (101 MHz, Chloroform-*d*) (6)

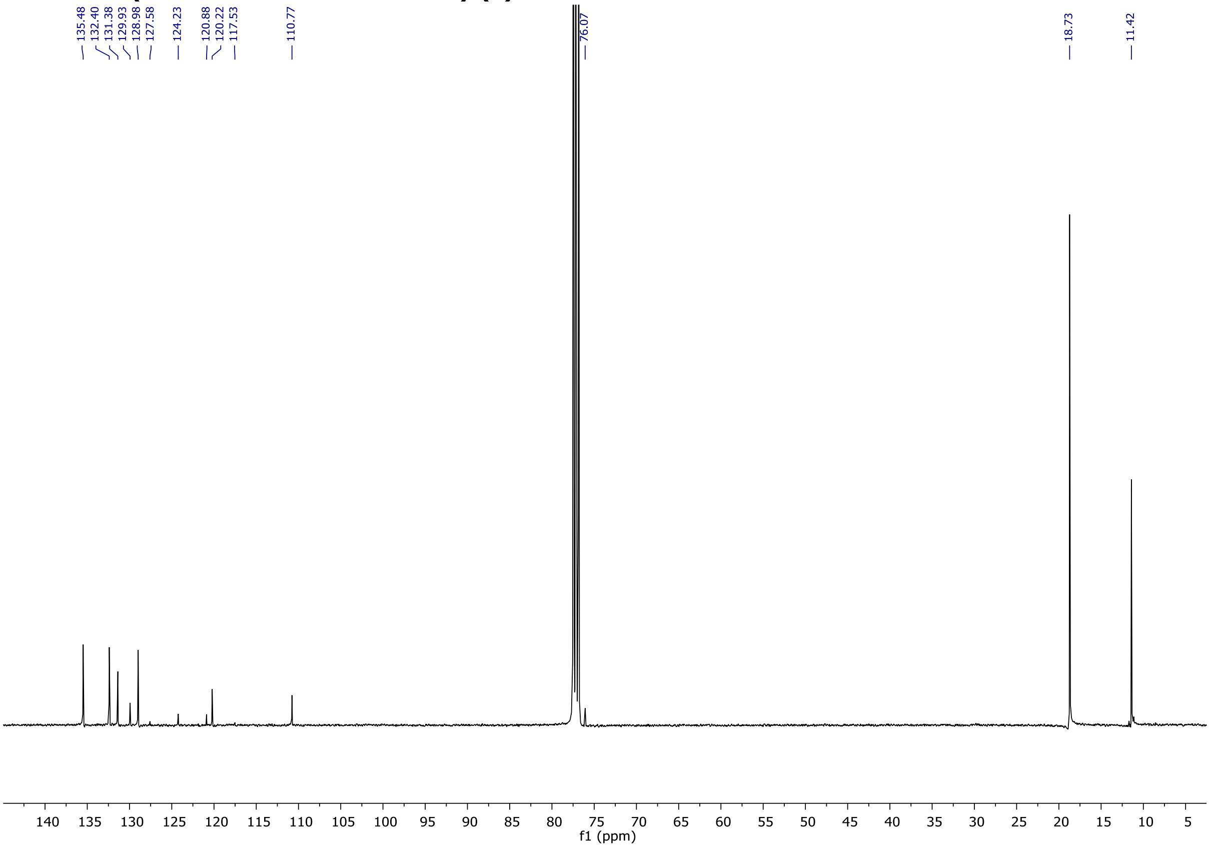

^{19}F-NMR (376 MHz, Chloroform-*d*) (6)

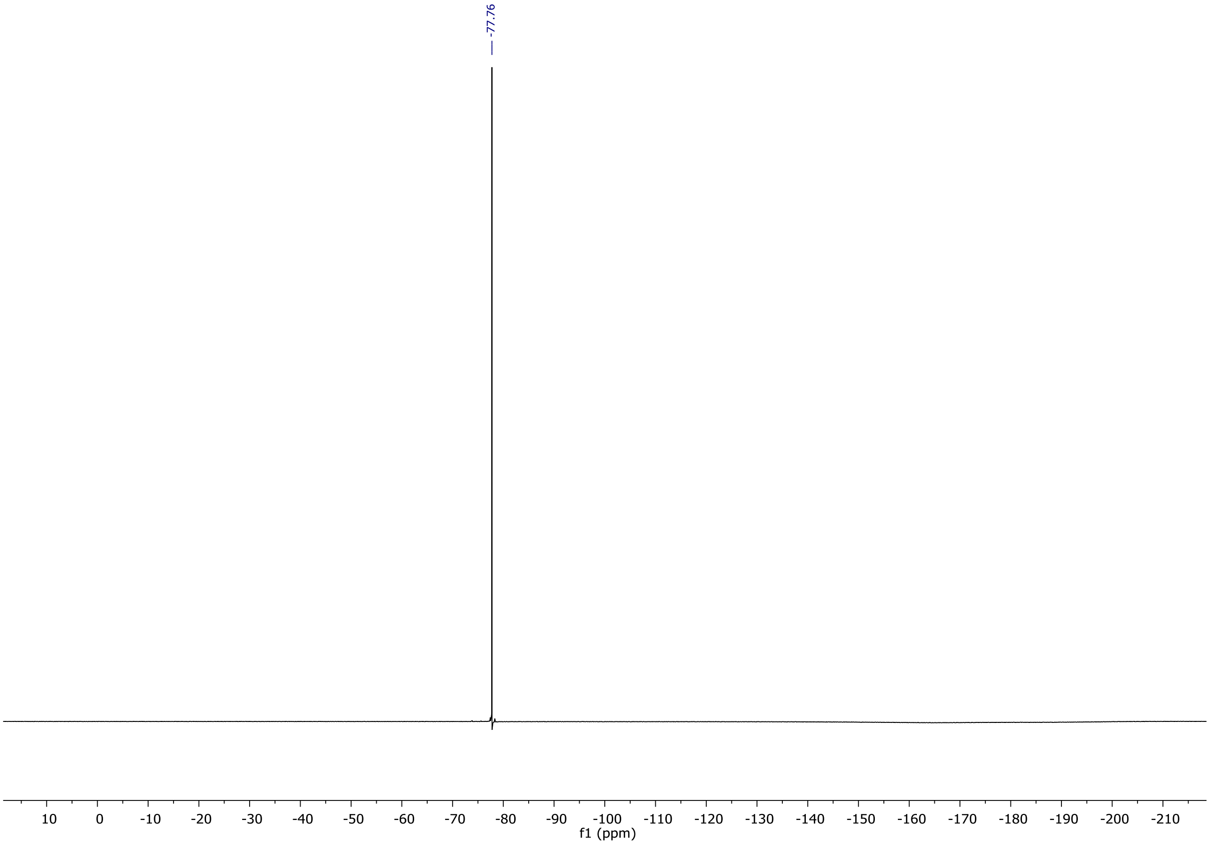

^{1}H-NMR (300 MHz, acetonitrile-d_3) 17a

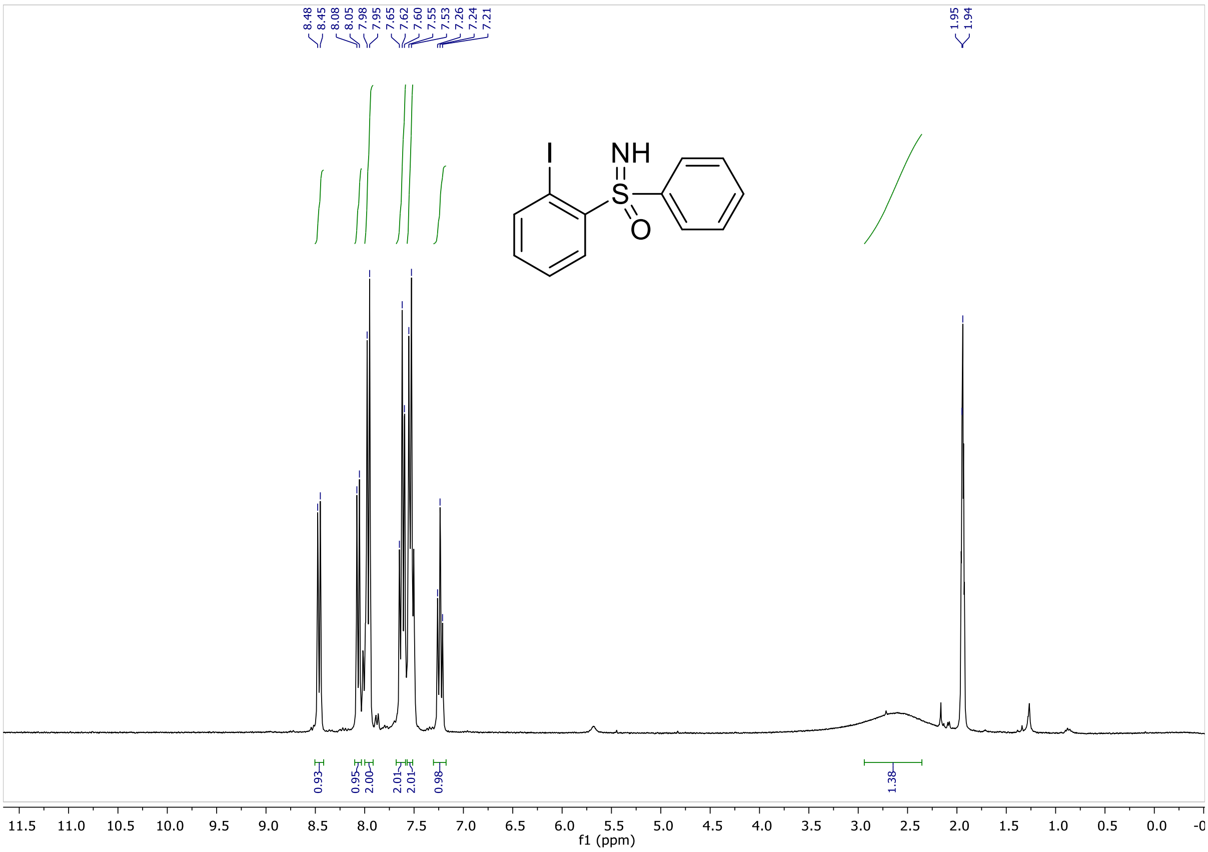

^{13}C-NMR (75 MHz, acetonitrile-d_3) 17a

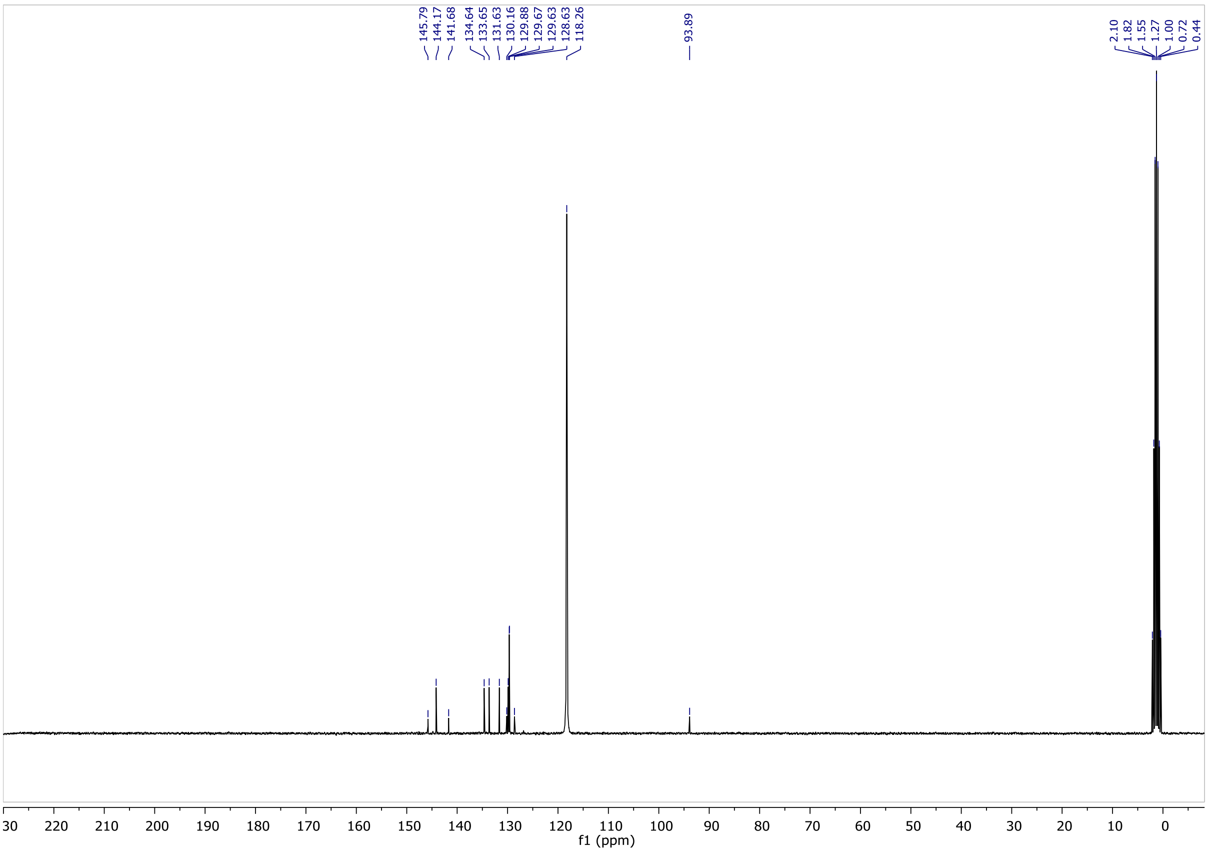

^{1}H-NMR (300 MHz, Chloroform-*d*) 17b

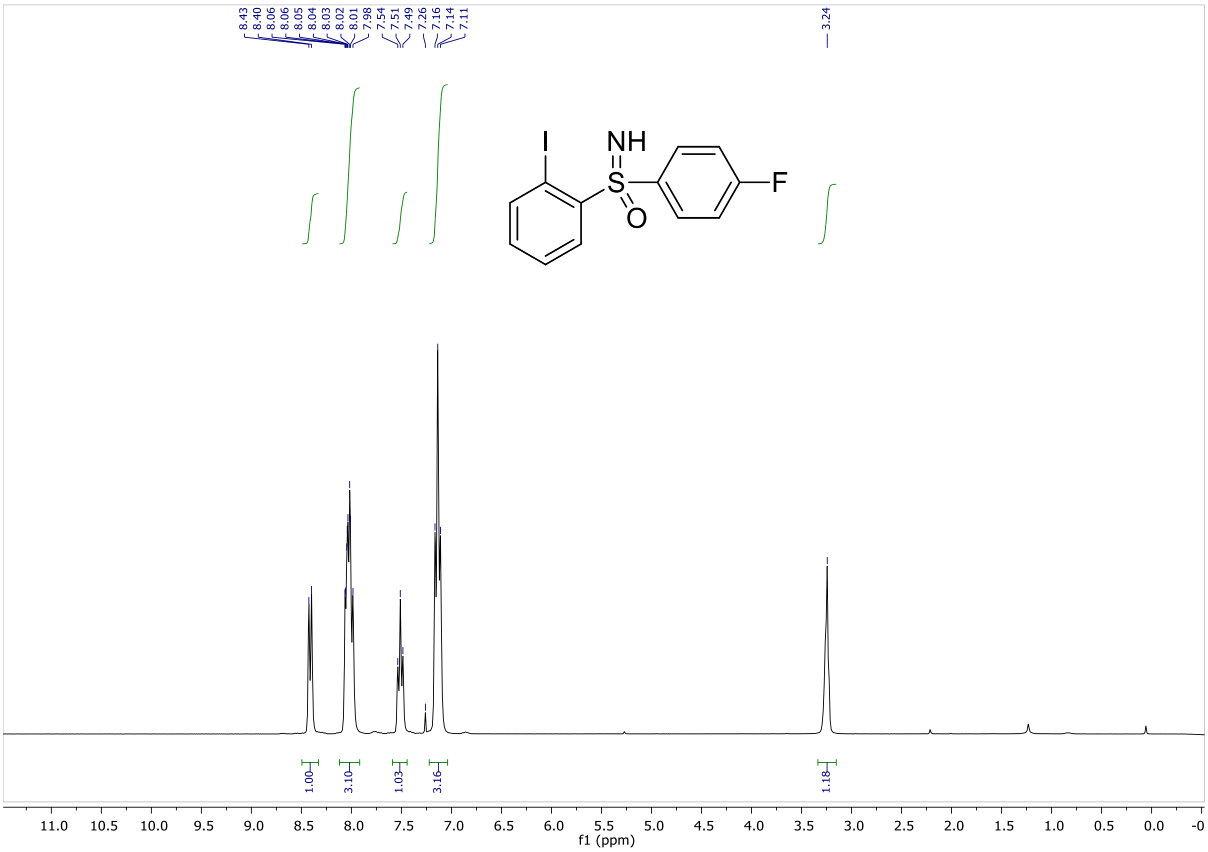

^{13}C-NMR (75 MHz, Chloroform-*d*) 17b

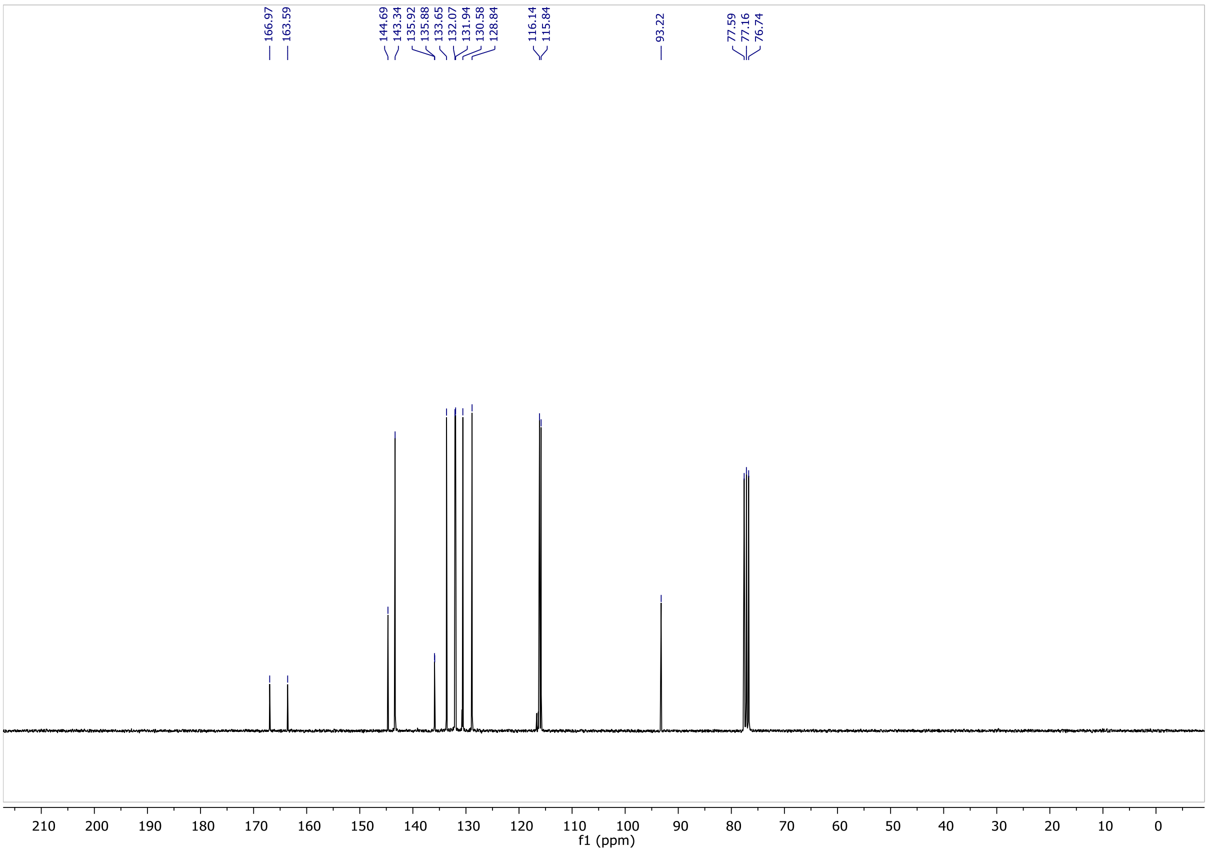

^{19}F-NMR (188 MHz, Chloroform-*d*) 17b

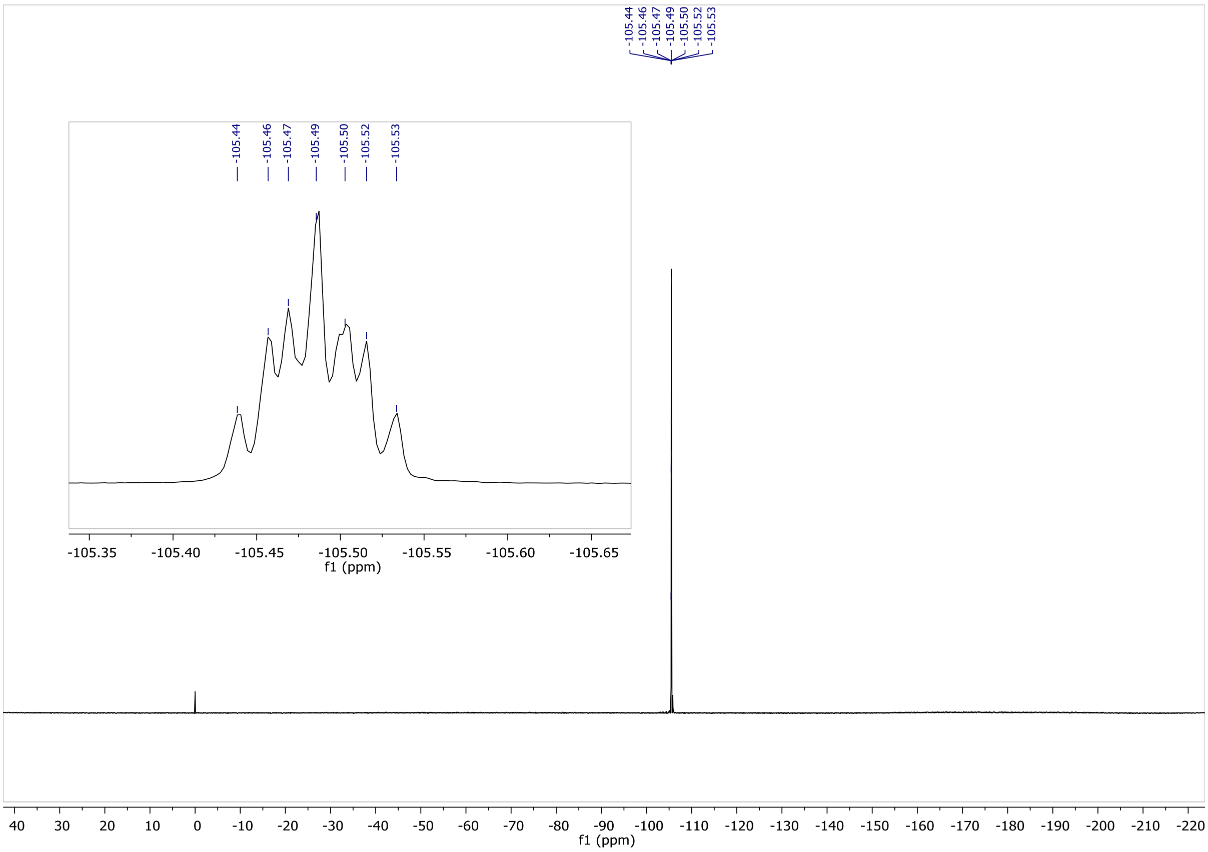

^{1}H-NMR (300 MHz, Chloroform-*d*) 17c

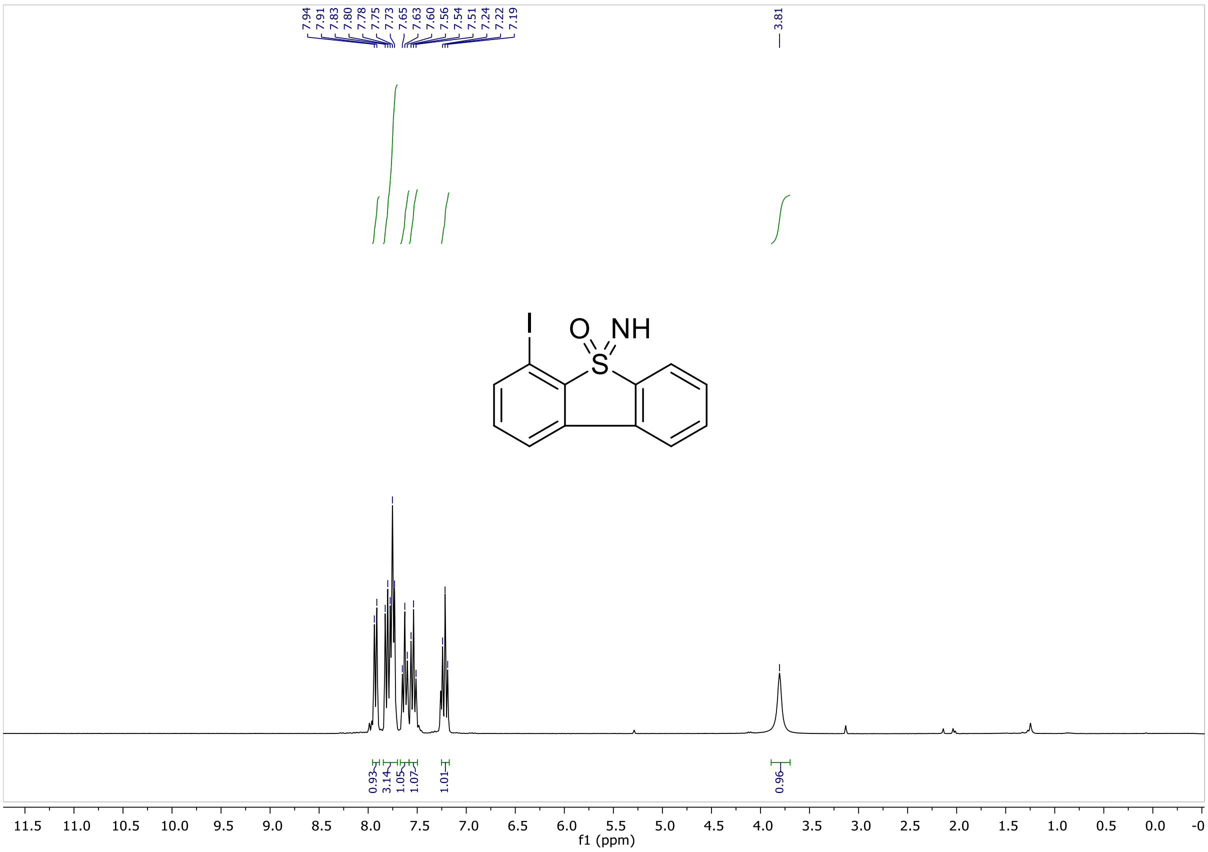

^{13}C-NMR (75 MHz, Chloroform-*d*) 17c

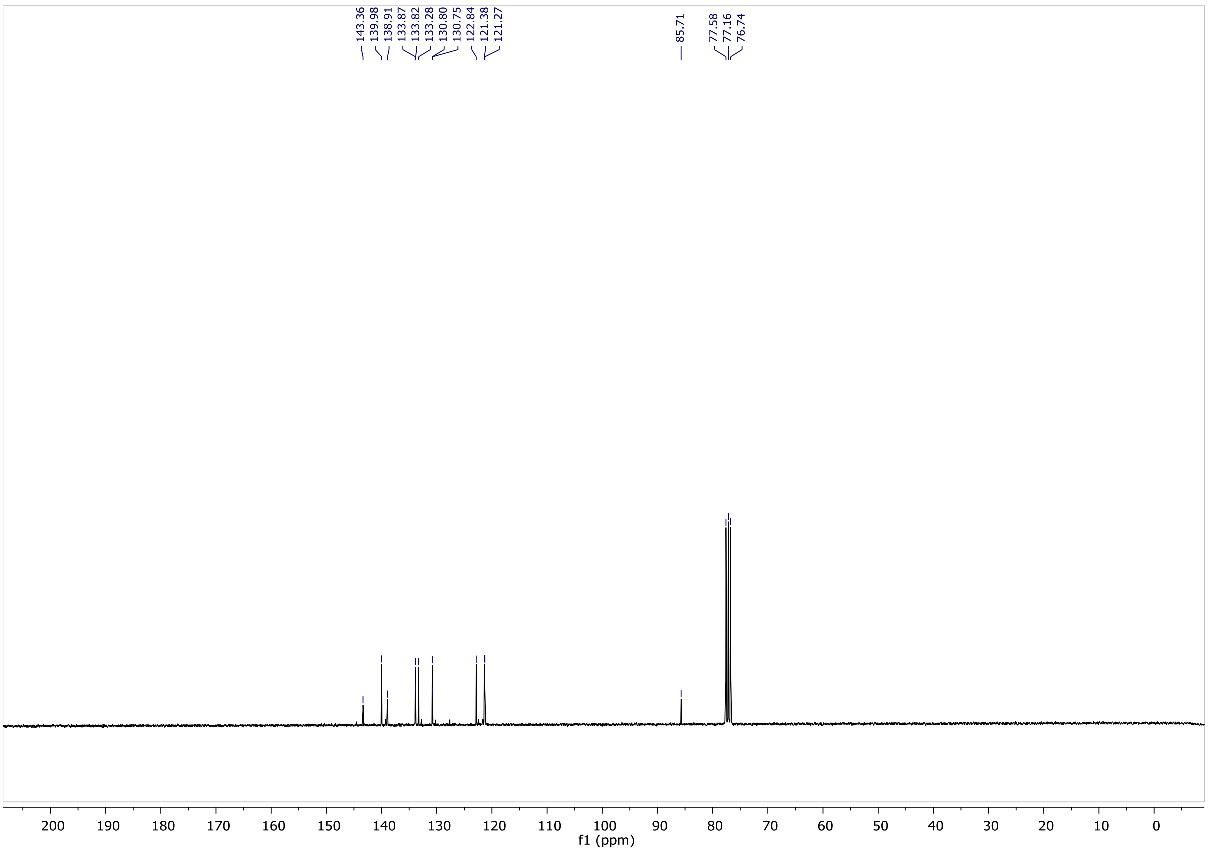

^{1}H-NMR (300 MHz, Chloroform-*d*) 15

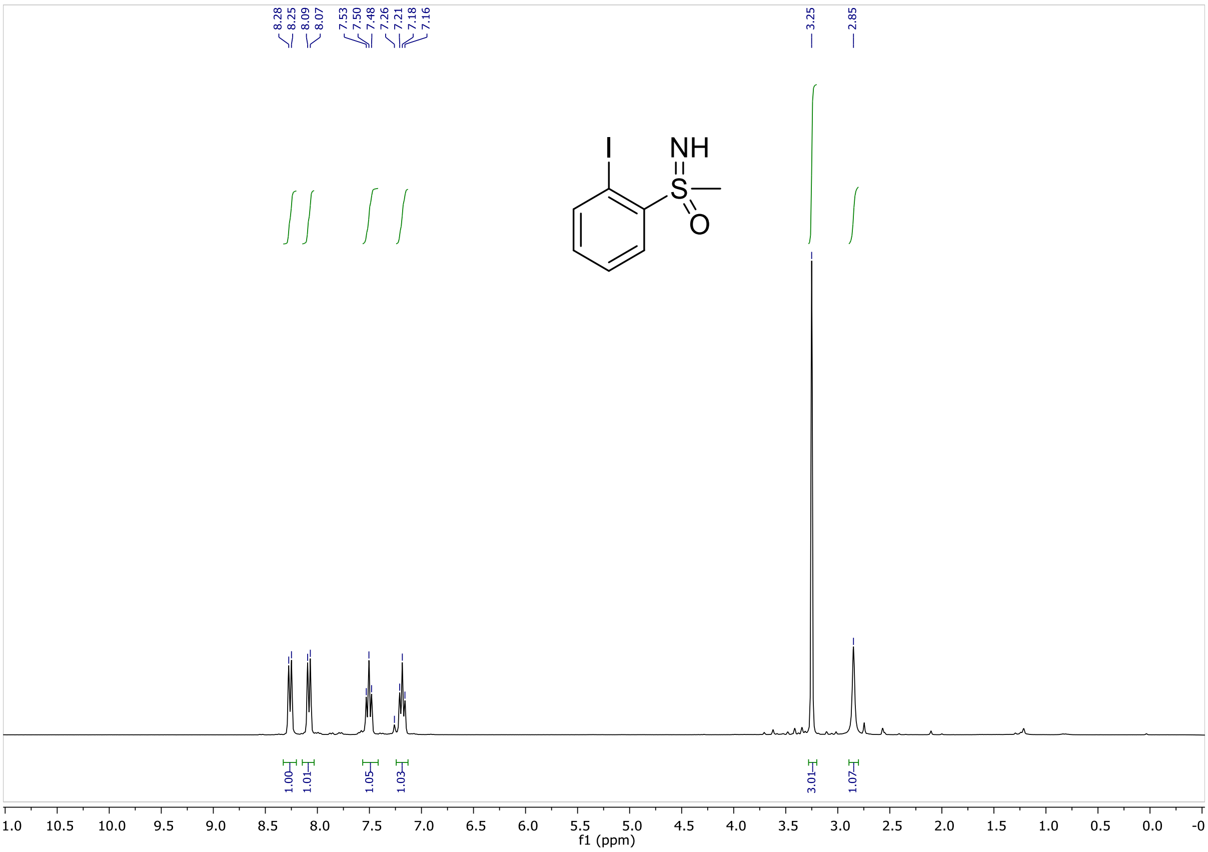

^{13}C-NMR (75 MHz, Chloroform-*d*) 15

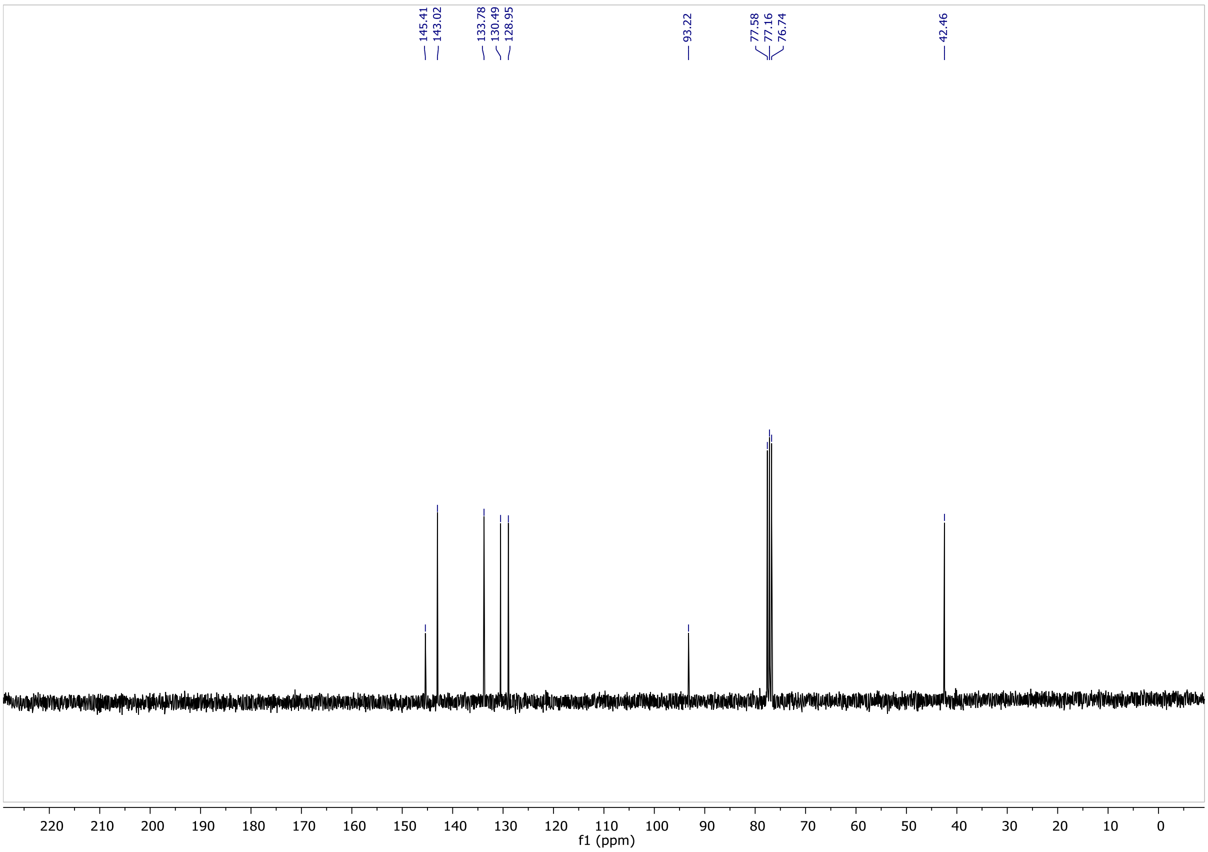

Supplement: Supplementary file 1 — Supporting Information [file CHEM-27-10979-s001.pdf]
